# Radical, Not Ionic: The Mechanochemical Zincke Nitration of Phenols

**DOI:** 10.1002/anie.7996981

**Published:** 2026-10-01

**Authors:** Roberto Scipione, Francesco Basoccu, Pietro Caboni, Francesca Sardu, Enrico Podda, Marco Lucarini, Massimiliano Arca, Andrea Porcheddu

**Affiliations:** ^1^ Dipartimento di Scienze Chimiche e Geologiche Università degli Studi di Cagliari Cagliari Italy; ^2^ Cittadella Universitaria di Monserrato Monserrato Italy; ^3^ Dipartimento di Chimica “Giacomo Ciamician” Alma Mater Studiorum – Università di Bologna Bologna Italy; ^4^ Centro Servizi di Ateneo per la Ricerca (CeSAR) Università degli Studi di Cagliari Cagliari Italy

**Keywords:** mechanochemistry, nitration, phenols, radical reactions, Zincke nitration

## Abstract

Nitrite‐mediated nitration of phenols is synthetically valuable, yet the mechanism of the classical Zincke nitration has remained insufficiently defined. Here, we show that nitrous acid, generated in situ from NaNO_2_ and NaHSO_4_ under liquid‐assisted vibrational mechanochemical conditions, enables both direct nitration of phenols and *ipso* nitration of halogenated phenols. The protocol applies to simple, substituted, electron‐rich, polycyclic and biologically relevant phenols while reproducing the halogen‐displacement reactivity historically associated with the Zincke nitration. Across 50 phenolic substrates, with isolated yields up to 98%, this mechanochemical platform extends Zincke‐type halogen‐displacement chemistry and makes phenoxyl‐radical oxidative pathways experimentally accessible under a solvent‐minimised regime. Atmosphere‐controlled experiments, NO_x_‐transfer studies, anisole controls, EPR detection of phenoxyl‐type radicals and hybrid DFT‐D3 calculations support a pathway involving *O*‐nitrosation, aryl nitrite fragmentation, aerobic NO‐to‐NO_2_ oxidation and radical coupling. These results recast the Zincke nitration as an open‐shell nitration manifold and show that mechanochemistry can render experimentally readable mechanistic features that are difficult to resolve under conventional solution conditions.

## Graphical abstract

Mechanochemistry recasts the classical Zincke nitration as an open‐shell process. Liquid‐assisted vibrational milling of phenols with in situ HNO_2_ delivers direct and *ipso* nitration across 50 substrates, while atmosphere, NO_x_‐transfer, EPR and DFT evidence expose a phenoxyl‐radical/NO_2_ manifold underlying this historically ionic transformation.

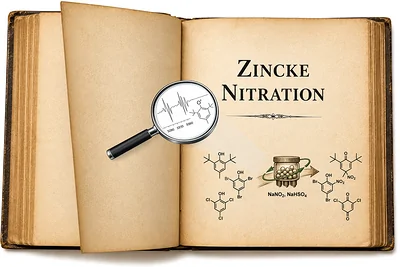

## 1 Introduction

Nitrophenols occupy a central position in synthetic, medicinal, agrochemical and materials chemistry because the nitro and hydroxy groups combine strong electronic differentiation with rich downstream functional‐group reactivity (Scheme 1a) [1, 2, 3, 4, 5, 6, 7, 8, 9, 10, 11]. Despite their broad relevance, their preparation still relies heavily on classical nitration methods, most often framed around electrophilic aromatic substitution. Such descriptions are useful at a formal level. However, they become less definitive when nitration is mediated by nitrite‐derived nitrogen oxides rather than by a preformed nitronium‐ion equivalent (Scheme 1b) [12, 13, 14, 15, 16, 17, 18, 19, 20, 21]. In these systems, strongly acidic media, multiple equilibrating nitrogen‐oxide species and transient nitroso or nitrite intermediates can make formally simple mechanistic assignments experimentally difficult to sustain.

**SCHEME 1:**
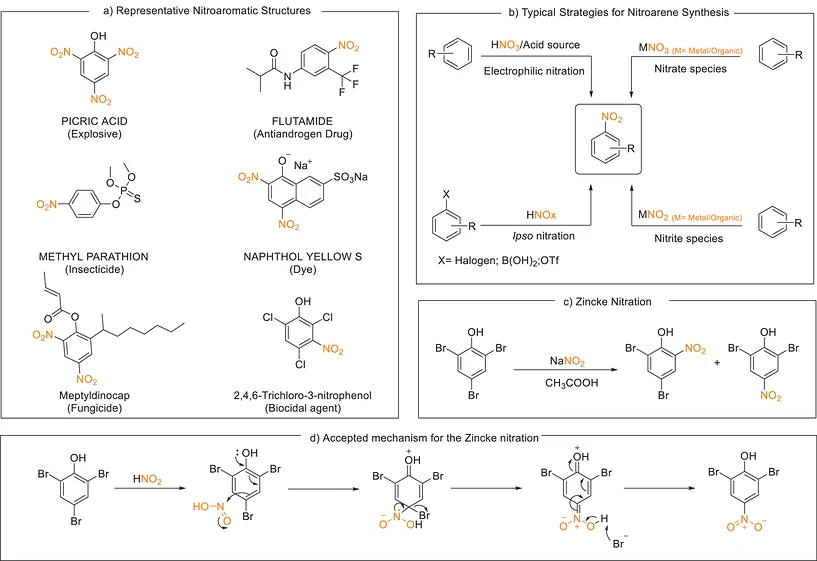
(a) Representative nitroaromatic structures of industrial, pharmaceutical and agrochemical relevance. (b) Typical synthetic approaches to nitroarenes, including electrophilic nitration and *ipso*‐nitration pathways. (c) Representative Zincke nitration of a halogenated phenol with in situ‐generated HNO_2_, leading to both *ortho*‐ and *para*‐nitrated products. (d) Commonly invoked ionic mechanism proposed in the literature for the Zincke nitration.

This ambiguity is especially evident in the Zincke nitration of halogenated phenols (Scheme 1c) [22, 23]. Since the earliest studies, the reaction has been described operationally as the replacement of hydrogen or halogen by a nitro group under acidic nitrite conditions [24, 25, 26, 27, 28, 29, 30]. The most common mechanistic representation remains ionic (Scheme 1d), yet direct experimental evidence for the key closed‐shell intermediates is limited. At the same time, the chemistry of nitrous acid, aryl nitrites, nitric oxide and nitrogen dioxide naturally opens alternative manifolds involving transient nitroso, nitrite and radical species [20, 31, 32, 33, 34, 35, 36, 37, 38, 39, 40]. Thus, the Zincke nitration remains a synthetically familiar but mechanistically underdefined transformation.

Mechanochemistry offers a powerful way to revisit this problem. Beyond reducing solvent use and intensifying mixing, mechanical activation creates highly concentrated, weakly solvated and heterogeneous environments in which reactive nitrogen‐oxide species may be generated, transferred and consumed before the bulk solution averages them out [41, 42, 43, 44, 45, 46, 47, 48, 49, 50]. In phenolic nitration, the same observable product can arise from mechanistically distinct sequences involving nitrosation, oxidation, radical capture and rearomatisation. Under conventional solution conditions, these elementary events can converge into a single apparent transformation, making ionic and radical descriptions difficult to separate experimentally. By contrast, a liquid‐assisted mechanochemical regime can amplify side pathways, trapping events and atmosphere‐dependent effects that are otherwise too transient to be diagnostic. Zincke nitration is therefore an ideal test case for enquiring whether mechanical activation can reveal, rather than merely accelerate, hidden reaction manifolds.

Here, we show that liquid‐assisted vibrational mechanochemistry provides both a synthetic platform and a mechanistic probe for nitrite‐mediated phenol nitration (Scheme 2). The in situ generation of HNO_2_ from NaNO_2_ and NaHSO_4_ enables direct nitration across a broad range of phenols. It extends to *ipso* nitration of halogenated substrates, thereby reproducing the defining reactivity of the Zincke reaction. More importantly, the same reaction environment reveals oxidative divergence, atmosphere dependence and radical signatures that are difficult to reconcile with a purely closed‐shell substitution pathway. Reactivity mapping, NO_x_‐transfer experiments, anisole controls, EPR spectroscopy and hybrid DFT‐D3 calculations collectively support an open‐shell manifold initiated by *O*‐nitrosation and aryl nitrite fragmentation. In this view, mechanochemistry does not simply translate the Zincke nitration into a solvent‐minimised format; it makes the radical chemistry underlying this historically important transformation experimentally readable.

**SCHEME 2:**
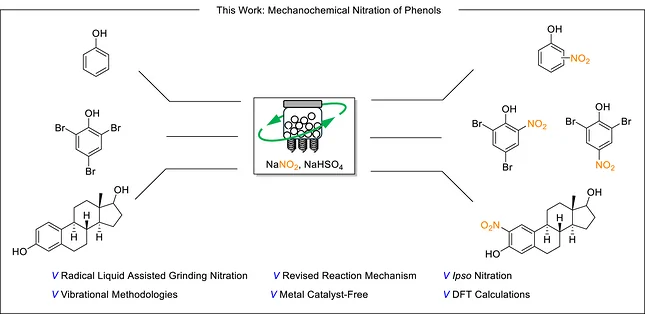
This work: liquid‐assisted vibrational mechanochemical nitration of phenols based on the in situ generation of HNO_2_ from NaNO_2_ and NaHSO_4_, enabling solvent‐minimised heterogeneous nitration, *ipso* nitration of halophenols and mechanistic access to a phenoxyl‐radical manifold.

## 2 Results and Discussion

### 2.1 Development of a Vibrationally Assisted Nitration Manifold

We began by examining whether nitrous acid, generated in situ from NaNO_2_ and NaHSO_4_, could be exploited as a synthetic reagent under vibrational mechanochemical conditions (Table 1). Phenol (**1a**) was selected as the initial model substrate because it provided a direct readout of both reactivity and regioselectivity, affording the corresponding *ortho‐* and *para‐*nitrated products, **2a′** and **2a′′,** respectively. Under neat conditions with 1.0 equiv of each reagent, the transformation proved inefficient, and no conversion was observed after 1 h of treatment (Table 1, entry 1). This outcome suggests that the reaction is controlled not solely by the intrinsic chemical competence of the nitrite/acid pair, but also by the heterogeneous mixture's ability to sustain reactive contact under mechanical agitation.

**TABLE 1:** Optimisation of the nitration protocol.

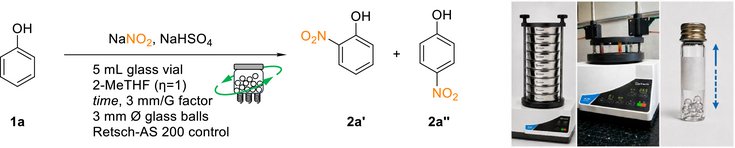							
Entry	NaNO_2_ (equiv.)	NaHSO_4_ (equiv.)	Liquid additive (*η* = 1)	Time	1a %^a^	2a’ %^a^	2a’’ %^a^
1	1	1	/	60 min	100	0	0
2	1	1	2‐MeTHF	60 min	60	19	21
3	2	2	2‐MeTHF	60 min	16	43	41
4	3	3	2‐MeTHF	60 min	6	37	50
5	3	3	2‐MeTHF	120 min	5	47	48
6	3	3	2‐MeTHF	180 min	0	52	48
7	3	3	Heptane	180 min	41	26	33
8	3	3	CHCl_3_	180 min	3	31	57
9	3	3	EtOAc	180 min	24	22	36
10	3	3	MeNO_2_	180 min	0	52	47

^a^ Reactions were performed by combining phenol **1a**, NaNO_2_ and NaHSO_4_ in a 5 mL glass vial equipped with 3 mm glass balls, in the absence or presence of the indicated liquid additive (*η* = 1 µL mg^−1^). Unless otherwise stated, one 3 mm glass ball, 35.6 mg, was used per 32 mg of total mass of the solid reagents and the vibrational amplitude was set to 3 mm (Retsch AS‐200 amplitude setting). Conversions and product distributions were determined by ^1^H NMR analysis. Values are averages of three independent runs. Unassigned mass balance corresponds to minor by‐products formed through collateral pathways.

Under liquid‐assisted grinding (LAG) conditions [51, 52], the transformation accelerated markedly (Table 1, entries 2–10), consistent with a scenario in which a limited amount of liquid enhances mixing, local mobility and interfacial exchange without restoring the reaction environment of a conventional solution process. The liquid additive should therefore not be viewed simply as a solubilizing medium. Rather, at the selected *η* value, it likely promotes transient interfacial mobility while preserving the high local concentrations and heterogeneous contacts required for productive NO_x_ chemistry.

Control experiments performed under otherwise comparable non‐milling conditions yielded substantially lower conversion/product formation, confirming that the observed reactivity is not simply a consequence of reagent combination in the presence of 2‐MeTHF but rather requires a mechanically renewed heterogeneous environment.

A screen of reagent loading showed that the combination of 3.0 equiv. of NaNO_2_ and 3.0 equiv. of NaHSO_4_ provided the most effective balance between conversion and product formation within 1 h (Table 1, entries 2–4). Extending the reaction time to 3 h resulted in complete consumption of phenol and delivered a near‐equimolar mixture of the *ortho‐* and *para‐*nitrated products, as determined by ^1^H NMR analysis (Table 1, entry 6).

The result is mechanistically informative in two respects. First, it establishes that nitrite‐derived phenol nitration can be rendered highly efficient under liquid‐assisted vibrational conditions. Second, the near‐equimolar *ortho*/*para* distribution is not, by itself, mechanistically diagnostic, because both ionic and radical pathways may furnish mixed regioisomers. In combination with the mechanistic evidence described below, however, it is consistent with the competitive capture at both positions of a delocalised radical intermediate. This interpretation is supported by literature EPR data on phenoxyl radicals, which indicate comparable radical character at the reactive ring positions [53]. The liquid additive exerted a substantial influence on performance. Among the media examined, 2‐MeTHF provided the best balance between conversion, product formation and operational sustainability (Table 1, entry 6). Heptane and ethyl acetate gave lower conversions, whereas chloroform and nitromethane were effective but less attractive from practical or environmental standpoints (Table 1, entries 7–10). Overall, the optimisation does more than define convenient reaction parameters: it identifies a physical regime in which reactive NO_x_ species can be generated and consumed under intimate, solvent‐minimised and incompletely solvated conditions.

### 2.2 Scope of Direct Nitration Across Simple and Complex Phenols

With the optimised conditions in hand, we examined the direct nitration of structurally diverse phenols (Scheme 3; see reagents and products in Schemes S1 and S2). A consistent pattern emerged across substrates bearing both available *ortho* and *para* positions: nitration occurred at both sites, with limited regioselectivity. Phenol **1a** afforded **2a′** and **2a′′** in 95% overall yield and an approximately 1:1 ratio.

**SCHEME 3:**
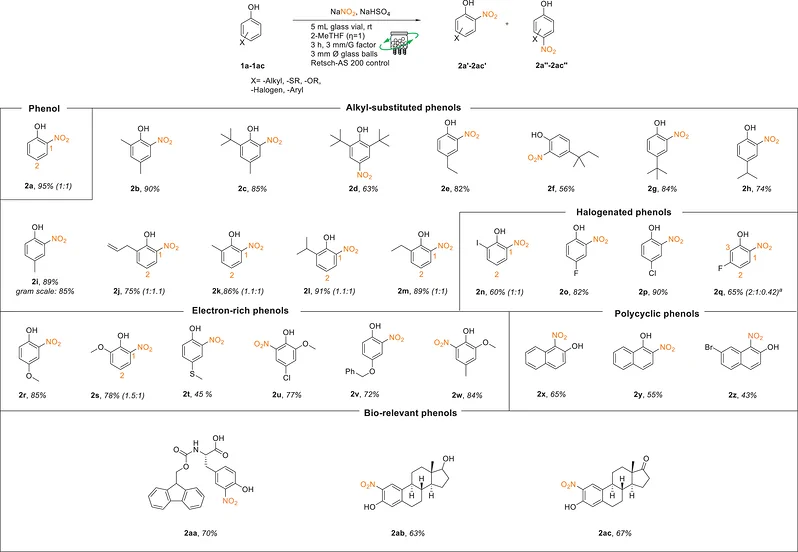
Scope of the liquid‐assisted mechanochemical nitration of phenols. Reactions were performed using phenolic substrates **1a**–**1ac** (1.0 mmol), NaNO_2_ (3.0 equiv.) and NaHSO_4_ (3.0 equiv.) under vibrational conditions [Retsch AS 200 amplitude setting; (3 mm/G)] for 3 h with 2‐MeTHF as liquid additive (*η* = 1). Isolated yields are reported as combined yields of the regioisomeric mixture; where two nitration sites were accessible, the ring positions are labelled **1** and **2** in the structures and the corresponding regioisomeric ratio (**1**:**2**) is given in parentheses, position 1 being *ortho* and position  2 *para* with respect to the phenolic OH group. In the main text and in Table 1, the individual regioisomers are denoted **2′** (position 1) and **2′′** (position 2). For substrate **1q**, three nitration sites were possible, and the corresponding products are denoted **2q′**, **2q′′** and **2q′′′**.

When the substitution pattern of the phenol derivatives provided a single accessible nitration site, clean mononitration was observed. Thus, phenols **1b**–**1d** afforded the corresponding products **2b**–**2d** in good yields. Likewise, substrates **1e**–**1i**, for which nitration was restricted to the position *ortho* to the hydroxy group, furnished nitrophenols **2e**–**2i** in synthetically useful yields. Notably, **2i** was prepared on a gram scale without appreciable loss of efficiency. In contrast, alkyl‐substituted phenols **1j**–**1m**, which contain multiple accessible nitration sites, afforded regioisomeric mixtures with only modest selectivity. The nearly statistical product distributions are not, by themselves, mechanistically diagnostic, since mixed regioisomers can arise from both closed‐shell and open‐shell pathways. Taken together with the mechanistic evidence discussed below, however, they are more readily accommodated by the formation and subsequent evolution of transient open‐shell intermediates than by a strongly directed electrophilic substitution.

Halogenated phenols **1n**–**1q** remained competent substrates. Electron‐rich phenols bearing alkoxy or thioether substituents afforded products **2r**–**2w** in 45%–85% yield, and naphthol derivatives **1x**–**1z** were also converted, although efficiency decreased as steric and electronic complexity increased.

The method further tolerated structurally elaborate phenols. *N*‐(9‐Fluorenylmethyloxycarbonyl)tyrosine (Fmoc‐Tyr), estradiol and estrone (**1aa**–**1ac**, Scheme S1) all underwent productive nitration to give the corresponding products in useful yields. These examples establish that the observed reactivity is not confined to electronically simple phenols but persists across substrates with increased steric demand, conformational complexity and embedded functional groups. The breadth of the scope, therefore, supports the mechanistic analysis developed below: the observed selectivity trends are not artifacts of a narrow model system but features of a coherent phenolic nitration manifold.

### 2.3 *ipso‐*Nitration of Halogenated Phenols and Oxidative Divergence as a Mechanistic Signature

We next examined trisubstituted phenols to test whether the mechanochemical manifold could reproduce the chemistry historically associated with the Zincke nitration (Scheme 4). This substrate class was particularly informative because productive nitration requires not only C–NO_2_ bond formation, but also replacement of a ring‐bound halogen atom at an already substituted position. Under the standard conditions, 2,4,6‐tribromophenol **3a** underwent smooth mononitration with concomitant displacement of a ring‐bound bromine atom, affording the *ortho*‐ and *para*‐substituted regioisomers **4a′** and **4a′′** in excellent combined yield and an approximately 1:1 ratio (Scheme 4). Thus, the mechanochemical protocol captures the defining feature of the classical transformation: formal replacement of a ring‐bound halogen atom by a nitro group under nitrite‐derived acidic conditions.

**SCHEME 4:**
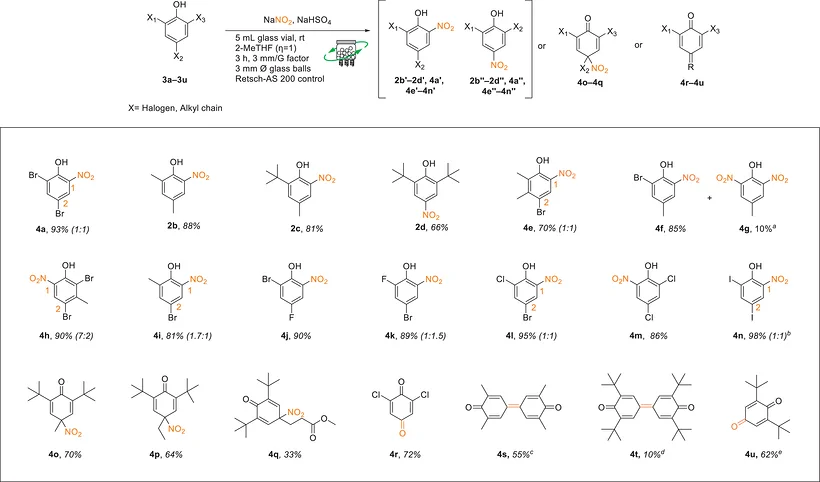
Scope of the mechanochemical nitration of trisubstituted phenols (see reagents and products in Schemes S3 and S4). Reactions were performed using phenolic substrates **3a–3u** (1.0 mmol), NaNO_2_ (3 equiv.) and NaHSO_4_ (3 equiv.) under vibrational conditions (Retsch AS 200, 3 mm/G) for 3 h in the presence of 2‐MeTHF as a liquid‐assisted grinding additive (*η* = 1). Isolated yields are reported. When two regioisomers were obtained, ratios are given as **4′**:**4′′**, where **4′** denotes *ortho* nitration and **4′′** denotes *para* nitration relative to the phenolic OH group. *^a^* Dinitrated product obtained from 4‐methyl‐2,6‐dibromophenol (**3f**). *^b^* Product obtained from 2,4,6‐triiodophenol (**3n**). *^c^* Product obtained from 3,3′,5,5′‐tetramethyl‐[1,1′‐biphenyl]‐4,4′‐diol (**3s**). *^d^* Minor dimeric oxidation by‐product obtained from 2,6‐di‐tert‐butyl‐4‐bromophenol (**3d**). *^e^* Product obtained from 2,4,6‐tri‐*tert*‐butylphenol (**3u**). Products also accessible by direct nitration retain their original numbering from Scheme 3 (**2b**–**2d**); the yields reported here refer to the corresponding halogenated substrates **3b**–**3d** and therefore differ from those obtained by direct nitration.

This behaviour extended to other brominated and iodinated phenols. Monobrominated substrates bearing alkyl substituents, **3b** and **3c**, afforded the corresponding *ipso*‐nitrated products in good to excellent yields. More substituted phenols also reacted efficiently, with mononitration predominating in most cases. Substrate **3f** yielded a minor amount of dinitrated product **4g**, along with mononitrated product **4f**, indicating that over‐functionalisation remains limited under the optimised conditions. Regioselectivity between accessible sites remained modest in most cases, although substrate **3h** showed a stronger preference for one regioisomer. This behaviour again argues against a rigid electrophilic‐directing model and instead points to selectivity controlled by radical formation, radical capture and subsequent rearomatisation or fragmentation.

The halogen effect was particularly revealing. In mixed halogenated substrates **3j**–**3m**, nitration occurred selectively at bromine‐bearing positions, whereas fluorine and chlorine were retained. A conventional, undifferentiated substitution pathway does not readily account for this reactivity trend; rather, it points to a mechanistic scenario in which *ipso* nitration is governed by the interplay among C–X bond strength, radical‐capture efficiency and the propensity of the resulting cyclohexadienone intermediate to undergo productive fragmentation.

Particularly striking was the behaviour of 2,4,6‐triiodophenol **3n**, which underwent the analogous transformation efficiently, showing that iodine can participate in the same productive manifold. Taken together, these results show that halogenated phenols do not merely tolerate the mechanochemical nitration conditions; they enter a regime in which *ipso* substitution becomes a robust expression of the same underlying reactivity landscape.

To assess potential friction‐induced heating during mechanochemical activation, the bulk temperature of the reaction mixture was monitored throughout the milling process. Starting from an initial mixture temperature of 30°C, the measured bulk temperature remained within 30°C–32°C throughout the experiment (see Graph S1). The absence of a sustained increase in bulk temperature indicates that the observed reactivity is not driven by bulk heating, although it does not exclude transient local hot spots at the collision sites.

When productive *ipso* substitution was precluded or sterically disfavoured, the same reaction manifold was diverted towards oxidative dearomatisation, providing an independent mechanistic signature of phenoxyl‐radical intermediacy. Thus, exposure of 2,6‐bis(*tert*‐butyl)‐4‐methylphenol (**3o**, BHT) to the standard conditions afforded the dearomatised nitrocyclohexadienone **4o**, whose quinoid cyclohexa‐2,5‐dien‐1‐one framework was unambiguously confirmed by single‐crystal X‐ray diffraction (Figure 1).

**FIGURE 1:**
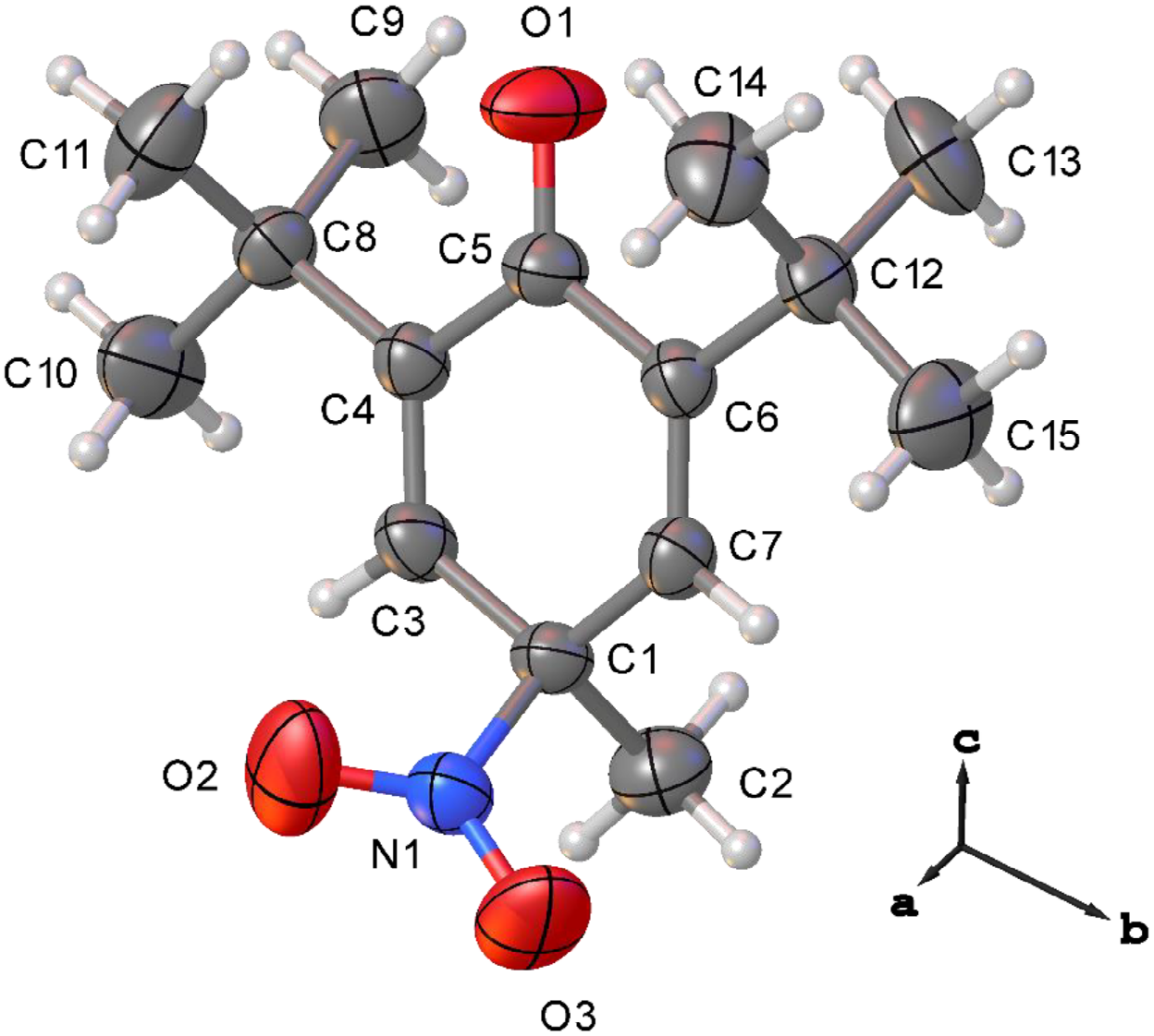
Ellipsoid representation of compound **4o** with the atomic numbering scheme adopted. Displacement ellipsoids are drawn at 50% probability level.

Compound **4o** crystallises in the monoclinic space group *P*2_1_/*n* with one crystallographically independent molecule in the asymmetric unit (*Z*' = 1; *Z* = 4; Tables S3–S5). The crystal packing is mainly governed by van der Waals contacts, with molecules paired into centrosymmetric $R_{2}^{\;2}\left(10\right)$ dimers through weak intermolecular C–H···O contacts involving the nitro group (Figure S1).

Related sterically hindered phenols behaved similarly, affording either analogous nitrocyclohexadienones or genuine quinoid oxidation products, in yields that, although variable, were fully consistent with the intervention of phenoxyl radical chemistry [54]. More complex cases provided even stronger support for this interpretation. A tetramethyl‐substituted biphenolic substrate (**3s**) underwent oxidative conversion to the corresponding 3,3′,5,5′‐tetramethyl‐4,4′‐diphenoquinone **4s**, whereas 2,4,6‐tri‐*tert*‐butylphenol (**3u**) afforded 2,6‐di‐*tert*‐butyl‐1,4‐benzoquinone **4u** through loss of the *para tert*‐butyl group, reproducing the well‐established oxidative behaviour of heavily shielded phenols under radical conditions [55]. These divergent reaction pathways reveal that nitration and oxidation originate from a common mechanistic manifold. The Zincke nitration, therefore, emerges not as an isolated transformation but as one manifestation of a broader open‐shell reactivity landscape unveiled under mechanochemical conditions.

### 2.4 Mechanistic Interrogation of the Nitration Manifold

Because no individual experiment can definitively resolve the interplay of ionic and radical processes in nitrite‐mediated phenol nitration, we combined atmosphere‐controlled reactions, NO_x_‐transfer studies, substrate controls, EPR spectroscopy and DFT calculations to assess whether a single open‐shell mechanistic manifold could consistently explain the collective experimental observations.

Having established both direct nitration and *ipso* substitution, we first asked whether the observed reactivity could be accommodated within the conventional ionic interpretation of the Zincke nitration, or whether the mechanochemical regime was revealing a different manifold. Atmosphere‐dependent experiments provided the first indication of the role of molecular oxygen (Scheme 5a,b). For experiments conducted under nitrogen, the loaded vial was subjected to three consecutive vacuum–nitrogen cycles and was then sealed under nitrogen during milling (see the Supporting Information). Under these conditions, phenol **1a** and 2,4,6‐tribromophenol **3a** showed 20% and 40% conversion, respectively, compared with complete conversion in air. Molecular oxygen is therefore not an absolute requirement for product formation, although it strongly enhances conversion. Residual nitration under nitrogen is consistent with access to NO_2_ through nitrous‐acid decomposition (reaction b in Scheme 7). In contrast, oxygen promotes productive nitration by oxidising NO to NO_2_ (reaction e in Scheme 7) [56]. The nitrogen EPR experiment described below separately demonstrates that formation of the persistent BHT‐derived radical does not require molecular oxygen.

**SCHEME 5:**
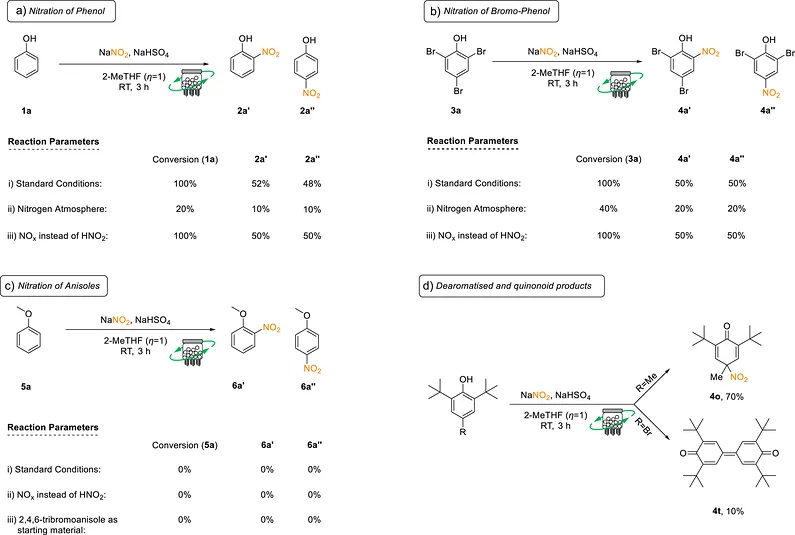
Control experiments and mechanistic probes for the liquid‐assisted mechanochemical nitration of phenols. (a,b) Comparison of phenol **1a** and 2,4,6‐tribromophenol **3a** under standard NaNO_2_/NaHSO_4_ conditions, under nitrogen and under NO_x_‐transfer conditions. (c) Anisole and 2,4,6‐tribromoanisole controls. (d) Oxidative divergence of sterically hindered phenols under standard conditions. Conversions and product distributions were determined by ^1^H NMR analysis unless otherwise stated.

To test whether gas‐phase nitrogen oxides are chemically competent under solvent‐minimised conditions, NO_x_‐transfer experiments were performed in an H‐tube setup. NO_x_ species were generated in one arm by reaction of copper with concentrated nitric acid (HNO_3_) and allowed to diffuse into the second arm, which contained the phenolic substrate under mechanochemical conditions.

Under ambient atmosphere, any NO formed can be oxidised by molecular oxygen to NO_2_, thereby maintaining a reactive NO_x_ mixture enriched in nitrogen dioxide (2 NO + O_2_ → 2 NO_2_) [57, 58]. Under these conditions, both phenol **1a** and 2,4,6‐tribromophenol **3a** underwent efficient nitration, with reactivity comparable to that observed in the direct NaNO_2_/NaHSO_4_ protocol [59]. These experiments show that transferred NO_x_ species, with NO_2_ as a chemically plausible nitrating component under air, can engage phenolic substrates in the mechanochemical environment.

The requirement for a free phenolic OH group was probed using anisole derivatives. Neither anisole **5a** nor 2,4,6‐tribromoanisole underwent productive nitration under the standard conditions, and anisole also remained unreacted in the NO_x_‐transfer experiment (Scheme 5c). *O*‐Methylation, therefore, suppresses the entry point into the productive manifold. This result is consistent with a mechanism in which the phenolic OH group first undergoes *O*‐nitrosation to form an aryl nitrite intermediate, which can then fragment to generate a phenoxyl radical. A methoxy group may reproduce part of the electronic activation of a phenol, but it cannot participate in the same *O*‐nitrosation/fragmentation sequence. Thus, the anisole controls argue against direct nitration of an electronically activated arene as the dominant entry point under these conditions and instead identify the phenolic OH group as a mechanistic handle for aryl nitrite formation.

The formation of dearomatised nitrocyclohexadienones and quinonoid oxidation products from sterically hindered phenols further supports the involvement of phenoxyl‐type intermediates. Cyclohexadienones and oxidative dimers are well precedented outcomes of phenoxyl‐radical chemistry [54], and their formation under the present conditions shows that the reaction environment can sustain open‐shell phenolic intermediates (Scheme 5d).

Although these products alone do not establish a common mechanism for every substrate, their formation, together with the O_2_ effect, NO_x_‐transfer experiments and anisole controls, justifies direct spectroscopic interrogation of radical intermediates. We therefore next examined persistent phenoxyl models by EPR spectroscopy (Scheme 6).

**SCHEME 6:**
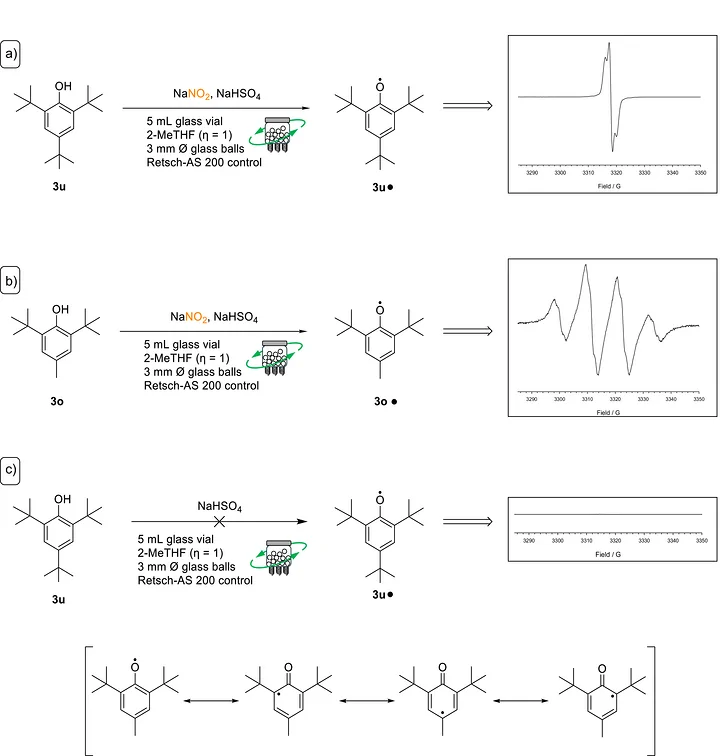
EPR spectra of phenoxyl radicals generated under standard conditions (NaNO_2_, NaHSO_4_, 2‐MeTHF, *η* = 1) in the presence of (a) **3u**, 2,4,6‐tri‐*tert*‐butylphenol and (b) **3o**, BHT. (c) Control experiment performed in the absence of NaNO_2_. Spectra were recorded immediately after transfer of the milled reaction mixture into the EPR cavity. Hyperfine coupling constants are reported in the text. Bottom: limiting resonance contributors of the BHT‐derived radical **3o**•, including the *para*‐carbon‐centred quinonoid form that rationalises the large a_Me_ value.

**SCHEME 7:**
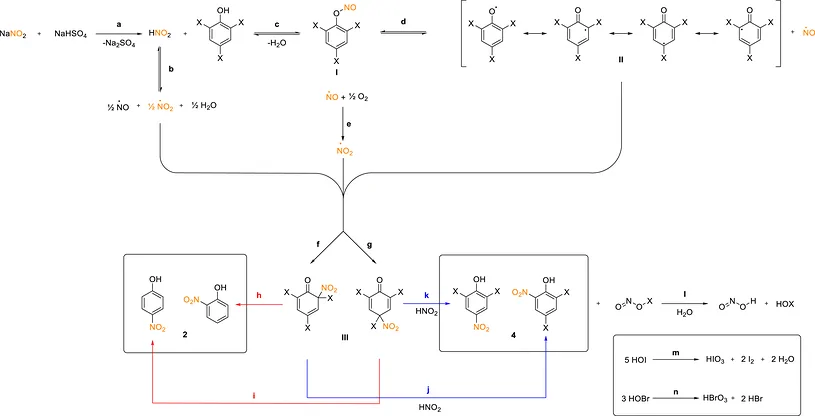
Proposed open‐shell pathway for liquid‐assisted mechanochemical phenol nitration and *ipso* nitration of halogenated phenols.

EPR spectroscopy provided direct evidence for the generation of phenoxyl‐type radicals under the reaction conditions (Scheme 6). For these studies, sterically hindered phenols were deliberately selected because the corresponding phenoxyl radicals are sufficiently persistent to accumulate to spectroscopically observable concentrations. The absence of an observable signal for less persistent halophenol‐derived radicals should therefore not be interpreted as evidence against their transient formation. Accordingly, 2,4,6‐tri‐*tert*‐butylphenol **3u** and BHT **3o** were selected as model substrates and treated with NaNO_2_/NaHSO_4_ under liquid‐assisted vibrational conditions in 2‐MeTHF.

For **3u**, immediate transfer of the reaction mixture to the EPR cavity revealed an intense signal with a poorly resolved triplet pattern, consistent with hyperfine coupling to the two equivalent aromatic hydrogen atoms at the 3‐ and 5‐positions, *a*_2H_ = 1.60 G [60]. The signal grew, reached a maximum, and then decayed, consistent with the progressive accumulation and subsequent consumption of the persistent phenoxyl radical **3u**• (Scheme 6a). No EPR signal was detected in the absence of NaNO_2_, confirming that nitrous acid generation is required for radical formation (Scheme 6c).

BHT **3o** behaved similarly. The corresponding phenoxyl radical **3o**• gave a broad, partially resolved quartet due to coupling with the three equivalent protons of the *para*‐methyl substituent, *a*_Me_ = 11.18 G (Scheme 6b) [60]. The large hyperfine coupling to the three *para*‐methyl protons (*a*_Me_ = 11.18 G) closely matches that reported for the 2,6‐di‐*tert*‐butyl‐4‐methylphenoxyl radical (typically ca. 10–11 G) [61, 62]. According to the McConnell relationship [63], this large coupling indicates substantial spin density at the *para* carbon. It thus provides direct spectroscopic evidence for extensive spin delocalisation over the phenoxyl π system, consistent with its quinonoid resonance contributors.

When the experiment was repeated under nitrogen‐degassed conditions, the EPR signal became more intense and better resolved, allowing additional coupling to the two aromatic protons (*a*_2H_ = 1.65 G) to be distinguished. These observations indicate that molecular oxygen is not required for the initial formation of the phenoxyl radical, although it remains important for productive nitration by oxidising NO to NO_2_.

No detectable EPR signal was observed for 2,6‐di‐*tert*‐butyl‐4‐bromophenol **3d**. Independent photolysis experiments [60] performed in the EPR cavity in the presence of peroxide also failed to generate an observable signal, suggesting that the corresponding phenoxyl radical is too short‐lived for direct detection under the conditions employed. The absence of a signal for **3d**, therefore, does not contradict a radical mechanism, but instead reflects the lifetime of the radical intermediate (Scheme 7). Taken together, the atmosphere dependence, NO_x_‐transfer experiments, anisole controls, oxidative divergence and EPR spectra support the involvement of open‐shell phenolic intermediates.

The mechanistic picture emerging from these experiments is summarised in Scheme 7. HNO_2_, generated in situ from NaNO_2_ and NaHSO_4_ (reaction a in Scheme 7), effects *O*‐nitrosation of the phenolic substrate to afford aryl nitrite **I**. Subsequent homolytic cleavage generates phenoxyl radical **II** together with NO, while aerobic oxidation of NO provides the NO_2_ radical required for productive nitration. Radical coupling between **II** and NO_2_ affords nitrocyclohexadienone intermediates **III**. In the case of simple phenols, **III** readily undergoes rearomatisation to afford the corresponding nitrophenols **2**. In contrast, for 2,4,6‐trihalophenols, direct rearomatisation is not possible because the nitrated carbon already bears a halogen substituent; consequently, the reaction proceeds through halogen elimination, ultimately leading to the *ipso*‐nitration products **4**. The formation of quinonoid oxidation products and the experimentally observed generation of molecular iodine (I_2_) from iodinated substrates (confirmed by Raman analysis, see Supporting Information) are also consistent with this radical manifold. Hybrid DFT‐D3 calculations were performed to evaluate the energetic viability of the pathway proposed in Scheme 7. To treat model open‐shell species [64, 65, 66], calculations were performed at the (U)M06‐2X‐D3/def2TZVP level of theory (see Supporting Information for computational details) [67, 68, 69]. The calculations were not intended to provide a complete kinetic description of the milling process, but to assess whether the elementary steps required by the proposed open‐shell manifold are thermochemically plausible and consistent with the observed substrate‐dependent reactivity.

The optimised geometry of crystallographically characterised compound **4o** agrees well with the experimental X‐ray structure (Figure S2), providing a structural consistency check for the selected computational method. Formation of aryl nitrite **I** by *O*‐nitrosation of the phenolic substrate with HNO_2_ is close to thermoneutral for phenol and mildly endergonic for the substituted models examined (reaction c in Scheme 7; Δ*G* = 1.69–6.03 kcal mol^−1^; Tables S6 and S10). Although aryl nitrite formation is mildly endergonic, the strongly exergonic downstream ArO•/NO_2_ coupling and rearomatisation steps render the overall nitration sequence thermodynamically favourable (Scheme 8). The mildly positive free energies calculated for aryl nitrite formation are consistent with the experimental use of an excess of the nitrite/acid pair, although they do not by themselves predict the required reagent stoichiometry. The following homolytic O–N bond cleavage of aryl nitrite **I** to give phenoxyl radical **II** and NO is calculated to be both endothermic and endergonic (reaction d in Scheme 7; Tables S6 and S10). Substitution at the 2, 4 and 6 positions by halogen atoms lowers the calculated energetic cost of this cleavage relative to the unsubstituted model. Within the 2,4,6‐trihalogenated series, however, the calculated differences among F, Cl, Br and I are comparatively small.

**SCHEME 8:**
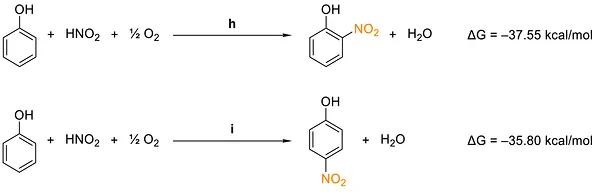
Calculated overall free energies for formation of the *ortho*‐ and *para*‐nitrated products from phenol **1a**, showing that neither pathway is strongly favoured on thermodynamic grounds.

Spin‐density analysis of **II** reveals significant radical character at the oxygen atom and at the *ortho* and *para* carbon positions (Table S7 and Figure S3), consistent with subsequent radical capture at these sites. The spin density at each individual *ortho* carbon is slightly lower than that at the *para* carbon; however, the presence of two equivalent *ortho* sites compensates statistically, so that the summed *ortho* contribution is comparable to the *para* one. This distribution is consistent with the near‐equimolar *ortho*/*para* product ratio, although spin populations alone do not determine the corresponding rate constants.

Aerobic oxidation of NO to NO_2_ is exergonic (reaction e in Scheme 7; Δ*H* = −10.81 and Δ*G* = −6.38 kcal mol^−1^; Tables S6 (reaction e) and S9–S10). Subsequent coupling of phenoxyl radical **II** with NO_2_ to form nitrocyclohexadienone intermediate **III** is also exergonic for all model substrates examined (reactions f and g in Scheme 7; Tables S6 and S10).

For phenol **1a**, rearomatisation of the corresponding nitrocyclohexadienone intermediates **III** affords *ortho*‐ and *para*‐nitrophenol **2a′** and **2a′′** (reactions h and i in Scheme 7, Tables S6 and S10) through overall balanced transformations with Δ*G* values of −37.55 and −35.80 kcal mol^−1^, respectively (reactions h and i; Scheme 8 and Table 2). The small difference between these values is consistent with the experimentally observed formation of both *ortho‐* and *para‐*nitrated products (Table 1). For 2,4,6‐trihalophenols, direct rearomatisation of **III** is not possible because the nitrated carbon already bears a halogen substituent. Therefore, the formation of compounds **4** requires a further reaction with nitrous acid (reactions j and k in Scheme 7; Tables S6 and S10) to achieve the C–X bond dissociation and halogen transfer (where X is a halogen).

**TABLE 2:** Calculated reaction enthalpies (Δ*H*) and free energies (Δ*G*), in kcal mol^−1^, for the overall nitration reactions, including formation of the nitrocyclohexadienone intermediates and the subsequent rearomatisation (X = H, footnote a) or halogen‐displacement step with HNO_2_ (X = F, Cl, Br, I, footnote b), at the (U)M06‐2X‐D3/def2TZVP level of theory.

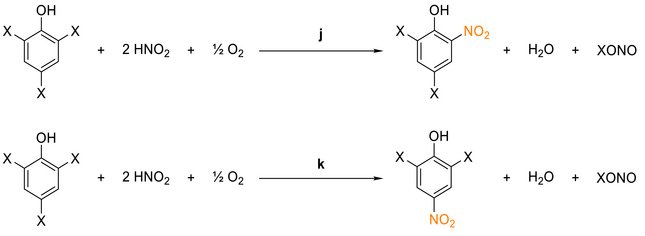						
		*2‐Nitration*		*4‐Nitration*		
	X	Δ*H*	Δ*G*	Δ*H*	Δ*G*	|ΔΔ*G*|
**1a**	H^a^	−45.35	−37.55	−43.06	−35.80	1.75
**3v**	F^b^	37.41	44.63	39.31	46.07	1.44
**3r**	Cl^b^	7.72	14.22	1.35	8.12	6.10
**3a**	Br^b^	−6.52	0.52	−6.30	0.47	0.05
**3n**	I^b^	−18.76	−11.58	−18.42	−11.58	0.01

^a^ For X = H, rearomatisation directly regenerates the aromatic nitrophenol.

^b^ For X = F, Cl, Br, I, productive nitration requires a further reaction with nitrous acid to achieve C─X dissociation and halogen transfer. **3v** (2,4,6‐trifluorophenol) was examined computationally only.

The σ* Kohn–Sham (KS) LUMO of **III** has antibonding character with respect to the C–X bond (illustrated for X = I in Figure S4, left), suggesting that this step could involve an interaction of the lone pair (LP) of electrons on the nitrogen atom of HNO_2_/NO_2_^−^ with the halogen atom of the relevant 2,4,6‐trihalo‐nitrocyclohexadienone. The calculated natural charges *Q* indicate an increasingly positive character at X on moving from F to I (*Q*_X_ = −0.297, 0.036, 0.100 and 0.198 |e| for X = F, Cl, Br and I, respectively, for the corresponding 2,4,6‐trihalo‐4‐nitrocyclohexadienones; *Q*_X_ = −0.284, 0.076, 0.138 and 0.233 |e| for X = F, Cl, Br and I, respectively, for the corresponding 2,4,6‐trihalo‐2‐nitrocyclohexadienones), in agreement with the molecular electrostatic potential (MEP) topology (Figure S4, right). These results are consistent with a possible nucleophilic halogen‐transfer pathway involving HNO_2_/NO_2_^−^.

The overall free energies calculated for the nitration reactions are strongly endergonic for X = F and remain unfavourable for X = Cl. In contrast, the brominated system is nearly thermoneutral, and the iodinated system is exergonic (Table 2). The near‐thermoneutrality calculated for the brominated systems refers only to the overall balanced transformation leading to XONO; the high experimental conversions are consistent with downstream consumption of the halogen‐containing species (reactions l–n) and with the large excess of the nitrite/acid pair, both of which shift the overall sequence towards the products. This trend is consistent with experimental halogen selectivity: bromine and iodine can be displaced readily, whereas fluorine and chlorine are retained in mixed‐halogenated substrates. Importantly, for the productive Br and I cases, the calculated free‐energy differences between the two possible nitration sites are negligible, consistent with the modest regioselectivity observed experimentally. While the identities of the halogen‐containing by‐products remain undetermined, halogen nitrites XONO represent plausible transient species in the proposed halogen‐transfer sequence (Scheme 7 and Table 2) and may subsequently evolve into more stable halogen species through reactions l–n. The detection of molecular iodine (I_2_) in the iodinated system (Figure S5) provides qualitative support for iodine release and transfer within the proposed reaction manifold; however, it does not directly identify XONO as an intermediate or independently demonstrate a radical mechanism.

Calculations on BHT **3o** also support oxidative divergence within the same manifold. Formation of the corresponding nitrocyclohexadienone products is calculated to be overall favoured, with the 4‐nitrocyclohexadienone pathway (Δ*G* = –19.28 kcal mol^−1^) preferred over the alternative 2‐nitration pathway (–14.14 kcal mol^−1^; Table S10). This accounts for the isolation of compound **4o** and reinforces the view that cyclohexadienone formation is not a disconnected side process, but an alternative fate of the same phenoxyl‐radical intermediates.

In summary, the hybrid DFT‐D3 calculations are consistent with the reactivity picture proposed above. Although the detailed energetics will depend on substrate architecture and the precise treatment of the condensed reaction environment, the combined EPR and hybrid‐DFT results are fully consistent with experimental trends and support a mechanistic picture in which radical intermediates are not secondary side‐path species but the organizing core of the transformation.

This mechanistic framework also provides a unified explanation for the behaviour of halogenated substrates in the Zincke nitration. Within a radical manifold, the divergence between direct nitration, *ipso* substitution and oxidation no longer appears anomalous. It emerges naturally from the interplay between radical addition, bond strength, intermediate stability and rearomatisation pathways. Bromine and iodine can be displaced because the corresponding intermediates are well‐positioned to fragment productively after radical capture, whereas more strongly retained substituents or sterically protected systems lacking an efficient substitution pathway are diverted towards oxidation. In this respect, the mechanochemical regime does more than support a useful synthetic method. It allows the Zincke nitration to be read not as an isolated historical curiosity but as a chemically intelligible expression of a broader landscape of radical nitration.

## 3 Conclusion

In summary, liquid‐assisted vibrational mechanochemistry enables efficient generation and use of nitrous acid from NaNO_2_ and NaHSO_4_ for the nitration of phenols. The protocol spans simple, substituted, electron‐rich, polycyclic and biologically relevant phenols and extends to the *ipso* nitration of brominated and iodinated phenols, as in the classical Zincke nitration. In sterically hindered substrates, the same conditions divert the reaction towards cyclohexadienone and dimeric oxidation products, indicating that productive nitration and oxidative divergence arise from a common open‐shell manifold. Atmosphere‐controlled experiments, NO_x_‐transfer studies, anisole controls, oxidative divergence, EPR spectroscopy and hybrid DFT‐D3 calculations converge on a pathway involving *O*‐nitrosation, aryl nitrite fragmentation, NO‐to‐NO_2_ oxidation and phenoxyl‐radical/NO_2_ coupling, rather than on a purely ionic substitution manifold. Within this framework, direct nitration, halogen displacement and oxidation are best understood as related outcomes of the same radical reactivity landscape. More broadly, this study shows that mechanochemistry can do more than improve the operational sustainability of known reactions: it can provide reaction environments in which otherwise obscured mechanistic pathways become experimentally accessible.

## Author Contributions

**Roberto Scipione**: investigation, methodology, data curation, formal analysis, validation. **Francesco Basoccu**: conceptualization, investigation, data curation, writing – original draft, writing – review and editing, methodology, visualization. **Pietro Caboni**: methodology, data curation, investigation. **Francesca Sardu**: investigation, methodology, validation, data curation. **Enrico Podda**: data curation, software, validation, formal analysis, methodology, writing – original draft. **Marco Lucarini**: funding acquisition, validation, methodology, writing – review and editing, formal analysis, data curation. **Massimiliano Arca**: investigation, writing – review and editing, software, formal analysis, data curation, writing – original draft, methodology, funding acquisition. **Andrea Porcheddu**: conceptualization, investigation, funding acquisition, writing – original draft, writing – review and editing, visualization, validation, project administration, data curation, supervision, resources, methodology.

## Conflicts of Interest

The authors declare no conflicts of interest.

## Supporting information



The Supporting Information contains experimental procedures, optimisation studies, substrate and product structures, characterisation data, NMR spectra, EPR experiments, NO_x_‐transfer experiments, X‐ray crystallographic data for **4o**, computational details, optimised geometries, spin‐density analyses and thermochemical tables. Deposition number CCDC 2560685 contains the supplementary crystallographic data for compound **4o**. These data can be obtained free of charge from The Cambridge Crystallographic Data Centre. The authors have cited additional references within the Supporting Information [70, 71, 72, 73, 74, 75, 76, 77, 78, 79, 80, 81, 82, 83, 84, 85, 86, 87, 88, 89, 90, 91, 92, 93, 94, 95, 96, 97, 98, 99, 100, 101, 102, 103, 104, 105, 106, 107, 108, 109, 110, 111, 112, 113, 114, 115, 116, 117, 118].
**Supporting File**: anie74867‐sup‐0001‐SuppMat.docx.

## Data Availability

The data that support the findings of this study are available in the Supporting Information of this article. Crystallographic data have been deposited at the Cambridge Crystallographic Data Centre under deposition number CCDC 2560685.
