# Supplementary material for: Radical, Not Ionic: The Mechanochemical Zincke Nitration of Phenols
Source: Angew Chem Int Ed Engl. 2026 Oct 1;65(46):e7996981. doi: 10.1002/anie.7996981 (PMC13749289; doi:10.1002/anie.7996981)
Supplement: Supplementary file 1 [file ANIE-65-e7996981-s001.docx]

Summary

[General Information	4](#_Toc239264967)

[Reaction Optimisation and Mechanistic Studies	6](#_Toc239264968)

[Optimisation Studies	6](#_Toc239264969)

[Solvent Screening	6](#_Toc239264970)

[Substrate Scope	7](#_Toc239264971)

[Experimental Procedures	9](#_Toc239264972)

[General procedure A for the synthesis of nitrophenols 2a-2ac	9](#_Toc239264973)

[General procedure B for the synthesis of products 2b–2d, 4a, and 4e–4u	9](#_Toc239264974)

[Scale-up procedure C for the synthesis of nitro-p-cresol derivatives from 4-methylphenol	10](#_Toc239264975)

[Mechanistic Studies	10](#_Toc239264976)

[Isolation of cyclohexadienone derivative under Standard Conditions	10](#_Toc239264977)

[Analysis of Molecular Iodine Formed during the Reaction of Iodinated Substrates	11](#_Toc239264978)

[Reaction of phenol under Inert Atmosphere	11](#_Toc239264979)

[Reaction of tribromo-phenol under Inert Atmosphere	12](#_Toc239264980)

[Reaction of Phenols with Externally Generated Nitrogen Dioxide in an H-Tube Setup	12](#_Toc239264981)

[Reaction of Anisole Derivatives under Standard Conditions.	13](#_Toc239264982)

[Reaction of Anisole with Externally Generated Nitrogen Dioxide in an H-Tube Setup.	13](#_Toc239264983)

[Temperature Monitoring during the Reaction of 2,4,6-Tribromophenol	14](#_Toc239264984)

[Control experiment under conventional solution conditions	15](#_Toc239264985)

[Crystallographic data	16](#_Toc239264986)

[DFT calculations	18](#_Toc239264987)

[Raman Analysis	44](#_Toc239264988)

[Characterization Data	45](#_Toc239264989)

[Products 2a–2ac	45](#_Toc239264990)

[2-nitrophenol **2a′**	45](#_Toc239264991)

[4-nitrophenol **2a′′**	45](#_Toc239264992)

[2,4-dimethyl-6-nitrophenol **2b**	45](#_Toc239264993)

[2-(*tert*-butyl)-4-methyl-6-nitrophenol **2c**	46](#_Toc239264994)

[2,6-di-*tert*-butyl-4-nitrophenol **2d**	46](#_Toc239264995)

[4-ethyl-2-nitrophenol **2e**	47](#_Toc239264996)

[2-nitro-4-(*tert*-pentyl)phenol **2f**	47](#_Toc239264997)

[4-(*tert*-butyl)-2-nitrophenol **2g**	47](#_Toc239264998)

[4-isopropyl-2-nitrophenol **2h**	48](#_Toc239264999)

[4-methyl-2-nitrophenol **2i**	48](#_Toc239265000)

[2-allyl-6-nitrophenol **2j′**	48](#_Toc239265001)

[2-allyl-4-nitrophenol **2j′′**	49](#_Toc239265002)

[2-methyl-6-nitrophenol **2k′**	49](#_Toc239265003)

[2-methyl-4-nitrophenol **2k′′**	49](#_Toc239265004)

[2-isopropyl-6-nitrophenol **2l′**	50](#_Toc239265005)

[2-isopropyl-4-nitrophenol **2l′′**	50](#_Toc239265006)

[2-ethyl-6-nitrophenol **2m′**	50](#_Toc239265007)

[2-ethyl-4-nitrophenol **2m′′**	51](#_Toc239265008)

[2-iodo-6-nitrophenol **2n′**	51](#_Toc239265009)

[2-iodo-4-nitrophenol **2n′′**	51](#_Toc239265010)

[4-fluoro-2-nitrophenol **2o**	52](#_Toc239265011)

[4-chloro-2-nitrophenol **2p**	52](#_Toc239265012)

[5-fluoro-2-nitrophenol **2q′**	52](#_Toc239265013)

[3-fluoro-4-nitrophenol **2q′′**	53](#_Toc239265014)

[3-fluoro-2-nitrophenol **2q′′′**	53](#_Toc239265015)

[4-methoxy-2-nitrophenol **2r**	53](#_Toc239265016)

[2-methoxy-6-nitrophenol **2s′**	54](#_Toc239265017)

[2-methoxy-4-nitrophenol **2s′′**	54](#_Toc239265018)

[4-(methylthio)-2-nitrophenol **2t**	54](#_Toc239265019)

[4-chloro-2-methoxy-6-nitrophenol **2u**	55](#_Toc239265020)

[4-(benzyloxy)-2-nitrophenol **2v**	55](#_Toc239265021)

[2-methoxy-4-methyl-6-nitrophenol **2w**	55](#_Toc239265022)

[1-nitronaphthalen-2-ol **2x**	56](#_Toc239265023)

[2-nitronaphthalen-1-ol **2y**	56](#_Toc239265024)

[7-bromo-1-nitronaphthalen-2-ol **2z**	56](#_Toc239265025)

[(((9H-fluoren-9-yl)methoxy)carbonyl)-3-nitro-L-tyrosine **2aa**	57](#_Toc239265026)

[*(*8R,9S,13S,14S)-13-methyl-2-nitro-7,8,9,11,12,13,14,15,16,17-decahydro-6H-cyclopenta[a]phenanthrene-3,17-diol **2ab**	57](#_Toc239265027)

[(8R,9S,13S,14S)-3-hydroxy-13-methyl-2-nitro-6,7,8,9,11,12,13,14,15,16-decahydro-17H-cyclopenta[a]phenanthren-17-one **2ac**	57](#_Toc239265028)

[Products 4a, 4e–4u	58](#_Toc239265029)

[2,4-dibromo-6-nitrophenol **4a′**	58](#_Toc239265030)

[2,6-dibromo-4-nitrophenol **4a′′**	58](#_Toc239265031)

[4-bromo-2,3-dimethyl-6-nitrophenol **4e′**	59](#_Toc239265032)

[6-bromo-2,3-dimethyl-4-nitrophenol **4e′′**	59](#_Toc239265033)

[2-bromo-4-methyl-6-nitrophenol **4f**	59](#_Toc239265034)

[4-methyl-2,6-dinitrophenol **4g**	60](#_Toc239265035)

[4,6-dibromo-3-methyl-2-nitrophenol **4h′**	60](#_Toc239265036)

[2,6-dibromo-3-methyl-4-nitrophenol **4h′′**	60](#_Toc239265037)

[4-bromo-2-methyl-6-nitrophenol **4i′**	61](#_Toc239265038)

[2-bromo-6-methyl-4-nitrophenol **4i′′**	61](#_Toc239265039)

[2-bromo-4-fluoro-6-nitrophenol **4j**	61](#_Toc239265040)

[4-bromo-2-fluoro-6-nitrophenol **4k′**	62](#_Toc239265041)

[2-bromo-6-fluoro-4-nitrophenol **4k′′**	62](#_Toc239265042)

[4-bromo-2-chloro-6-nitrophenol **4l′**	62](#_Toc239265043)

[2-bromo-6-chloro-4-nitrophenol **4l′′**	63](#_Toc239265044)

[2,4-dichloro-6-nitrophenol **4m**	63](#_Toc239265045)

[2,4-diiodo-6-nitrophenol **4n′**	63](#_Toc239265046)

[2,6-diiodo-4-nitrophenol **4n′′**	64](#_Toc239265047)

[2,6-di-*tert*-butyl-4-methyl-4-nitrocyclohexa-2,5-dien-1-one **4o**	64](#_Toc239265048)

[2,6-di-*tert*-butyl-4-ethyl-4-nitrocyclohexa-2,5-dien-1-one **4p**	64](#_Toc239265049)

[Methyl 3-(3,5-di-*tert*-butyl-1-nitro-4-oxocyclohexa-2,5-dien-1-yl)propanoate **4q**	65](#_Toc239265050)

[2,6-dichlorocyclohexa-2,5-diene-1,4-dione **4r**	65](#_Toc239265051)

[3,3′,5,5′-tetramethyl-[1,1′-bi(cyclohexylidene)]-2,2′,5,5′-tetraene-4,4′-dione **4s**	65](#_Toc239265052)

[3,3′,5,5′-tetra-*tert*-butyl-[1,1′-bi(cyclohexylidene)]-2,2′,5,5′-tetraene-4,4′-dione **4t**	66](#_Toc239265053)

[2,6-di-*tert*-butylcyclohexa-2,5-diene-1,4-dione **4u**	66](#_Toc239265054)

[NMR spectra	67](#_Toc239265055)

[References	138](#_Toc239265056)

# General Information

Commercially available reagents were purchased from Acros, Aldrich, Strem Chemicals, Alfa Aesar, and TCI Europe and used as received. All reactions were monitored by thin-layer chromatography (TLC) performed on glass-backed silica gel 60 F254, 0.2 mm plates (Merck), and compounds were visualized under UV light (254 nm) or using cerium ammonium molybdate solution with subsequent heating. Eluents were technical-grade solvents and were used without further purification. Mechanochemical reactions were conducted using a Retsch AS200 apparatus. The reagents were mixed using a glass vial (5 mL) equipped with glass balls (1 ball *ϕ* = 3 mm, ratio number of balls: weight of the whole mixture = 1 glass ball: 32 mg of total mass of the solid reagents, Mass per glass ball = 35.6 mg). For the scale-up experiment, a 25 mL glass vial equipped with 5 mm glass balls was used (1 ball *ϕ* = 5 mm, ratio number of balls: weight of the whole mixture = 1 glass ball: 141 mg of reacting mixture, Mass per glass ball = 164 mg). The vibratory mixing process was performed at an amplitude setting of 3.00 mm/G, as displayed by the AS200 instrument. This parameter defines the intensity of the oscillatory motion applied to the reaction vessel during mixing. These parameters were applied if not stated otherwise. ^1^H and ^13^C NMR spectra were recorded on a Varian 600 MHz and Bruker Avance III HD 600 MHz NMR spectrometer at 298 K and were calibrated using tetramethylsilane (TMS). Proton chemical shifts are expressed in parts per million (ppm, *δ* scale) and refer to the solvent’s residual hydrogen (CHCl_3_ - 7.260 ppm or DMSO - 2.50 ppm). Data are represented as follows: chemical shift, multiplicity (s = singlet, d = doublet, t = triplet, q = quartet, h = heptet, m = multiplet and/or multiple resonances, br s = broad singlet, and combination thereof), coupling constant (*J*) in Hertz (Hz) and integration. Carbon chemical shifts are expressed in parts per million (ppm, *δ* scale) and are referenced to the carbon resonances of the NMR solvent (CDCl₃, *δ* = 77.160 ppm or DMSO-d₆ *δ* = 39.520 ppm). Deuterated NMR solvents were obtained from Aldrich. Yields refer to pure, isolated materials.

Single-crystal X-ray diffraction data for **4o** were collected at 293 K on a Bruker D8 Venture diffractometer equipped with a PHOTON II detector using Mo Kα radiation (*λ* = 0.71073 Å). Data reduction was performed with SAINT,^[1]^ and multi-scan absorption corrections were applied using SADABS-2016/2.^[2]^ The structure was solved by dual-space methods with SHELXT^[3]^ and refined by full-matrix least-squares on *F*² with SHELXL.^[4]^ Olex2 v1.5^[5]^ was used as the graphical user interface. Hydrogen atoms were placed in calculated positions and refined using a riding model. CCDC 2560685 contains the supplementary crystallographic data for this paper. These data can be obtained free of charge from The Cambridge Crystallographic Data Centre via [www.ccdc.cam.ac.uk/structures](http://www.ccdc.cam.ac.uk/structures).

EPR spectra were recorded immediately after mixing the reactants and transferring the reaction mixture into a 2 mm quartz EPR tube. When required, samples were degassed by bubbling nitrogen prior to spectral acquisition.

EPR spectra were recorded on a Bruker ELEXYS spectrometer equipped with a microwave frequency counter for accurate determination of the *g*-factors, which were corrected with respect to the perylene radical cation in concentrated H_2_SO_4_ (*g* = 2.00258). The instrumental settings were as follows: microwave power, 0.79 mW; modulation amplitude, 0.05–0.1 mT; modulation frequency, 100 kHz; scan time, 180 s; and 2K data points.

Photolysis experiments were performed by irradiating samples directly within the EPR cavity using an ER 203UV 100 W UV Irradiation System, which provides illumination over the 200–800 nm wavelength range.

QM calculations were carried out at the Density Functional Theory (DFT) methods,^[6–13]^ which represents a convenient computational tool capable of accurately describing the structural, spectroscopic, and reactivity of organic^[14]^ and inorganic radicals.^[15]^ In a recent benchmarking comparative investigation,^[14]^ the hybrid meta-GGA functional M06-2X-D3,^[8]^ including Grimme’s dispersion corrections,^[9]^ emerged as a successful level of theory, especially when paralleled by triple-ζ split valence basis sets, such as Def2-TZVP.^[10]^ Accordingly, all calculations, performed with the Gaussian 16 computational suite,^[7]^ adopted the functionals M06-2X,^[8]^ complemented with Grimme dispersion corrections^[9]^ and the Def2-TZVP^[10]^ basis sets for all atomic species, including effective core potentials (ECPs) for heavy atomic species. Calculations were carried out on phenol (**1a**), 2,4,6-trihalo-substituted phenols (**3v**, trifluoro; **3r**, trichloro; **3a**, tribromo; and **3n**, triiodo) and 2,6-bis(*tert*-butyl)-4-methylphenol (**3o**, BHT), taken as model compounds. Notably, compound **3v** (2,4,6-trifluorophenol) was considered exclusively as a computational model. The nature of the minima of each optimized geometry was verified by harmonic frequency calculations. Charge distribution and Wiberg bond indexes^[11]^ were evaluated at NBO level.^[12]^ Thermochemical calculations were carried out at *T* = 298.15 K at the optimized geometries as a part of harmonic frequency calculations. Reaction enthalpies ΔH and free energy ΔG were calculated as differences of the unscaled sums of electronic and thermal enthalpies and free energies, corrected for Zero-Point Energies (ZPEs), calculated for the products and the reagents. GaussView 6.0.16^[13]^ was used to investigate the optimized structures, KS-MOs, total electron densities, molecular electrostatic maps, and spin densities.

Reaction Optimisation and Mechanistic Studies

## Optimisation Studies

**Table S1.** Optimisation table for the synthesis of nitrophenols.

| Entry | NaHSO₄ (equiv) | NaNO₂ (equiv) | 2-MeTHF (µL, η=1) | Time (min) | Balls | ^b^ NMR composition (%) | | | |
| --- | --- | --- | --- | --- | --- | --- | --- | --- | --- |
|  |  |  |  |  |  | **1a** | **2a′** | **2a′′** | **Imp** |
| 1 | 1 | 1 | 283 | 60 | 9 | 60 | 19 | 21 | 00 |
| 2 | 2 | 2 | 472 | 60 | 16 | 16 | 43 | 41 | 00 |
| 3 | 3 | 3 | 661 | 60 | 22 | 06 | 37 | 50 | 07 |
| 4 | 1 | 1 | 283 | 120 | 9 | 60 | 20 | 20 | 00 |
| 5 | 2 | 2 | 472 | 120 | 16 | 14 | 41 | 45 | 00 |
| 6 | 3 | 3 | 661 | 120 | 22 | 05 | 47 | 48 | 00 |
| 7 | 1 | 1 | 283 | 180 | 9 | 60 | 20 | 20 | 00 |
| 8 | 2 | 2 | 472 | 180 | 16 | 17 | 43 | 40 | 00 |
| 9 | 3 | 3 | 661 | 180 | 22 | 00 | 52 | 48 | 00 |
| 10 | 1 | 1 | // | 60 | 9 | 100 | 00 | 00 | 00 |

^a^Reactions were performed by combining phenol **1a**, NaNO₂ and NaHSO₄ in a 5 mL glass vial in the absence or presence of the indicated liquid additive (η factor = 1). Unless otherwise stated, one 3 mm glass ball, 35.6 mg, was used per 32 mg of reaction mixture, and the vibrational intensity was set to 3 mm/G. ^b^Conversions and product distributions were determined by ¹H NMR analysis. Values are averages of three independent runs.

## Solvent Screening

**Table S2.** Optimisation table for the liquid additive in the synthesis of nitrophenols.

| Entry | NaHSO₄ (equiv) | NaNO₂ (equiv) | LAG (η=1) | Balls | ^b^ NMR composition (%) | | | |
| --- | --- | --- | --- | --- | --- | --- | --- | --- |
|  |  |  |  |  | **1a** | **2a′** | **2a′′** | **imp** |
| 1 | 3 | 3 | CHCl_3_ | 22 | 03 | 31 | 57 | 9 |
| 2 | 3 | 3 | Hexane | 22 | 03 | 46 | 51 | 0 |
| 3 | 3 | 3 | DCM | 22 | 40 | 30 | 27 | 03 |
| 4 | 3 | 3 | EtOAc | 22 | 24 | 22 | 36 | 18 |
| 5 | 3 | 3 | CH_3_NO_2_ | 22 | 00 | 52 | 47 | 01 |
| 6 | 3 | 3 | THF | 22 | 00 | 48 | 48 | 04 |
| 7 | 3 | 3 | Toluene | 22 | 00 | 47 | 53 | 00 |
| 8 | 3 | 3 | MeCN | 22 | 00 | 42 | 54 | 04 |
| 9 | 3 | 3 | Heptane | 22 | 41 | 26 | 33 | 00 |

^a^Reactions were performed by combining phenol **1a**, NaNO₂ and NaHSO₄ in a 5 mL glass vial in the presence of the indicated liquid additive (η factor = 1). Unless otherwise stated, one 3 mm glass ball, 35.6 mg, was used per 32 mg of reaction mixture, and the vibrational intensity was set to 3 mm/G for 180 min. ^b^Conversions and product distributions were determined by ¹H NMR analysis. Values are averages of three independent runs.

## Substrate Scope

**Scheme S1**. Structures of the phenolic substrates (**1a–1ac**).

**Scheme S2.** Structures of the nitrated products (**2a–2ac**) obtained from the mechanochemical nitration of phenols shown in Scheme S1. Isolated yields are reported. When applicable, regioisomeric ratios are indicated in parentheses. The two possible positions for nitration are denoted as position 1 (*ortho*) and position 2 (*para*), and the corresponding products are indicated as **2xx′** (*ortho*) and **2xx′′** (*para*).*^a^* The three possible positions for nitration are denoted as position 1,3 (*ortho*), position 2 (*para*), and the corresponding products are indicated as **2q′** (*ortho-1*), **2q′′** (*para*-2) and **2q′′′** (*ortho-3)* with their regioisomeric ratio respectively reported in brackets.

**Scheme S3.** Structures of the phenolic substrates (**3a–3v**). The substrate **3v** was considered only as a computational model.

**Scheme S4.** Structures of the nitrated products (**2b–2d, 4a, and 4e–4u**) obtained from the mechanochemical nitration of phenols shown in Scheme S3. Isolated yields are reported. When applicable, regioisomeric ratios are indicated in parentheses. The two possible positions for nitration are denoted as position 1 (*ortho*) and position 2 (*para*), and the corresponding products are indicated as **xx′** (*ortho*) and **xx′′** (*para*). *ᵃ* Dinitrated product obtained from **3f**. *^b^* Product obtained from 2,4,6-triiodophenol. *^c^* Product obtained from 3,3′,5,5′-tetramethyl-[1,1′-biphenyl]-4,4′-diol (**3s**). *^d^* Product obtained from 2,6-di-*tert*-butyl-4-bromophenol (**3d**). *^e^* Product obtained from 2,4,6-tri-*tert*-butylphenol (**3u**). Products identical to those reported in Scheme S2 retain the numbering **2b–2d**.

# Experimental Procedures

## General procedure A for the synthesis of nitrophenols 2a-2ac

A 5 mL glass vial, equipped with glass milling balls of 3 mm in diameter (approximately one ball per 30–32 mg of total mass of the solid reagents, depending on the substrate), was charged with 2-MeTHF (η factor = 1), phenols **1a**–**1ac** (1.00 mmol), NaNO₂ (3.00 mmol), and NaHSO₄ (3.00 mmol). The vial was then closed, and the mechanochemical reaction was performed for 180 min at an amplitude of 3 mm/G. Upon completion of the reaction, the crude mixture was dissolved in ethyl acetate (3 mL) and filtered through a paper filter. The solvent was then removed under reduced pressure, and the resulting crude product was purified by flash column chromatography using a hexane/EtOAc gradient (95:5 to 80:20, v/v) as eluent to afford the pure nitrophenol derivatives **2a′**–**2ac′** and **2a′′**–**2ac′′**.

## General procedure B for the synthesis of products 2b–2d, 4a, and 4e–4u

A 5 mL glass vial, equipped with glass milling balls of 3 mm in diameter (approximately one ball per 30–32 mg of total mass of the solid reagents, depending on the substrate), was charged with 2-MeTHF (η factor = 1), substituted phenols **3a–3u** (1.00 mmol), NaNO₂ (3.00 mmol), and NaHSO₄ (3.00 mmol). The vial was then closed, and the mechanochemical reaction was performed for 180 min at an amplitude of 3 mm/G. Upon completion of the reaction, the crude mixture was dissolved in ethyl acetate (3 mL) and filtered through a paper filter. The solvent was then removed under reduced pressure, and the resulting crude product was purified by flash column chromatography using a gradient of hexane/EtOAc (95:5 to 80:20, v/v) as eluent to afford the pure nitrophenol derivatives **2b–2d, 4a, and 4e–4u**.

## Scale-up procedure C for the synthesis of nitro-p-cresol derivatives from 4-methylphenol

A 25 mL glass vial was charged with 4-methylphenol **1i** (1.08 g, 10.00 mmol), NaNO₂ (2.07 g, 30.00 mmol), and NaHSO₄ (3.60 g, 30.00 mmol). 2-MeTHF was then added as liquid-assisted grinding agent in an amount corresponding to η = 1 μL/mg with respect to the total mass of the solid reagents (6.75 mL). The vial was equipped with glass milling balls of 5 mm in diameter (corresponding to approximately one ball per 141 mg of total reaction mixture, depending on the substrate), and the reaction mixture was subjected to vibrational mixing for 180 min at an amplitude of 3 mm/G. Upon completion of the reaction, the crude mixture was dissolved in ethyl acetate (15 mL) and filtered through paper. The solvent was then removed under reduced pressure, the resulting crude product was purified by flash column chromatography using a gradient of hexane/EtOAc (95:5 to 80:20, v/v) as eluent to afford the corresponding nitrated 4-methylphenol derivatives **2i** (1.30 g, 8.50 mmol, 85%).

# Mechanistic Studies

## Isolation of cyclohexadienone derivative under Standard Conditions

A 5 mL glass vial equipped with 3 mm glass milling balls was charged with 2,6-di-tert-butyl-4-methylphenol **3o** (220 mg, 1.00 mmol), NaNO₂ (207 mg, 3.00 mmol), and NaHSO₄ (360 mg, 3.00 mmol). 2-MeTHF was added as liquid-assisted grinding agent (0.788 mL, η = 1 µL mg⁻¹), and the reaction mixture was subjected to mechanochemical activation in a Retsch AS 200 control apparatus for 180 min at room temperature and 3 mm amplitude. After completion, the crude mixture was dissolved in ethyl acetate (3 mL), filtered through paper, and concentrated under reduced pressure. The residue was purified by flash chromatography on silica gel using hexane/ethyl acetate as eluent to afford 2,6-di-tert-butyl-4-methyl-4-nitrocyclohexa-2,5-dien-1-one **4o** as the isolated product (186 mg, 0.70 mmol, 70%). The crystalline structure of compound **4o** was investigated by single-crystal X-ray diffraction analysis. Suitable crystals were obtained by slow evaporation of a CH₂Cl₂ solution at room temperature (see Figure S1).

## Analysis of Molecular Iodine Formed during the Reaction of Iodinated Substrates

A 5 mL glass vial equipped with 3 mm glass milling balls (approximately one ball per 30–32 mg of total mass of the solid reagents, depending on the substrate) was charged with 2,4,6-triiodophenol **3n** (472 mg, 1.00 mmol), NaNO₂ (207 mg, 3.00 mmol), and NaHSO₄ (360 mg, 3.00 mmol). 2-MeTHF was added as liquid-assisted grinding agent (1.039 mL, η = 1 µL mg⁻¹), and the reaction mixture was subjected to mechanochemical activation in a Retsch AS 200 control apparatus for 180 min at room temperature and 3 mm amplitude. After completion of the reaction, the crude mixture was dissolved in ethyl acetate and filtered through paper. The solvent was removed under reduced pressure, and the resulting crude residue was purified by chromatography/filtration through a short silica gel column using hexane as eluent. A violet-colored solution was collected from the hexane fraction. The solvent was removed under reduced pressure, and the obtained solid was purified by sublimation using a sublimator. The presence of molecular iodine was confirmed by Raman spectroscopy (see Figure S5).

## Reaction of phenol under Inert Atmosphere

A 5 mL glass vial equipped with a septum and 3 mm glass milling balls was charged with phenol **1a** (94 mg, 1.00 mmol), NaNO₂ (207 mg, 3.00 mmol), and NaHSO₄ (360 mg, 3.00 mmol). After addition of 2-MeTHF as liquid-assisted grinding agent (0.661 mL, η = 1 µL mg⁻¹), the vial was sealed and subjected to three vacuum–nitrogen cycles through the septum to establish an inert atmosphere. The reaction mixture was subjected to mechanochemical activation in a Retsch AS 200 control apparatus for 180 min at room temperature and 3 mm amplitude. After completion, the crude mixture was dissolved in ethyl acetate (3 mL), filtered through paper, and concentrated under reduced pressure. The crude reaction mixture composition was determined by ¹H NMR spectroscopy and compared with the corresponding experiment performed under air. Under inert atmosphere, the crude mixture contained nitrophenols **2a′/2a″** and unreacted phenol **1a** in an overall ratio of 20:80.

## Reaction of tribromo-phenol under Inert Atmosphere

A 5 mL glass vial equipped with a septum and 3 mm glass milling balls was charged with 2,4,6-tribromophenol **3a** (331 mg, 1.00 mmol), NaNO₂ (207 mg, 3.00 mmol), and NaHSO₄ (360 mg, 3.00 mmol). After addition of 2-MeTHF as liquid-assisted grinding agent (0.898 mL, η = 1 µL mg⁻¹), the vial was sealed and subjected to three vacuum–nitrogen cycles through the septum to establish an inert atmosphere. The reaction mixture was subjected to mechanochemical activation in a Retsch AS 200 control apparatus for 180 min at room temperature and 3 mm amplitude. After completion, the crude mixture was dissolved in ethyl acetate (3 mL), filtered through paper, and concentrated under reduced pressure. The crude reaction mixture composition was determined by ¹H NMR spectroscopy and compared with the corresponding experiment performed under air. Under inert atmosphere, the crude mixture contained the regioisomeric dibromonitrophenols **4a′/4a″** and unreacted 2,4,6-tribromophenol **3a** in an overall ratio of 40:60.

## Reaction of Phenols with Externally Generated Nitrogen Dioxide in an H-Tube Setup

The appropriate phenolic substrate (1.00 mmol) was placed in the right chamber of an H-tube together with 2-MeTHF corresponding to η = 1 µL mg⁻¹ with respect to the substrate mass. In the left chamber, nitrogen dioxide was generated in situ from copper powder (95 mg, 1.50 mmol) and concentrated nitric acid (ca. 420 µL, 6.00 mmol, 65 wt%) according to the stoichiometry Cu + 4HNO₃ → Cu(NO₃)₂ + 2NO₂ + 2H₂O, corresponding to 3.00 equiv of NO₂ with respect to the phenolic substrate. The H-tube was assembled and sealed, and the substrate-containing chamber was magnetically stirred at room temperature for 180 min, allowing gaseous NO₂ generated in the opposite chamber to diffuse into the stirred substrate-containing chamber. After completion, the reaction mixture was collected and analyzed by ¹H NMR spectroscopy. Complete conversion of the starting phenolic substrate was observed, affording the corresponding nitrated phenol derivative(s) as the only detectable product(s).

## Reaction of Anisole Derivatives under Standard Conditions.

A 5 mL glass vial equipped with 3 mm glass milling balls was charged with the appropriate anisole derivative (1.00 mmol), NaNO₂ (207 mg, 3.00 mmol), and NaHSO₄ (360 mg, 3.00 mmol). After addition of 2-MeTHF as liquid-assisted grinding agent (η = 1 µL mg⁻¹), the vial was sealed and subjected to mechanochemical activation in a Retsch AS 200 control apparatus for 180 min at room temperature and 3 mm amplitude. After completion, the crude mixture was dissolved in ethyl acetate, filtered through paper, and concentrated under reduced pressure. Analysis of the crude reaction mixture by ¹H NMR spectroscopy showed only the recovered anisole derivative, with no detectable formation of nitrated products.

## ***Reaction of Anisole with Externally Generated*** Nitrogen Dioxide ***in an H-Tube Setup.***

Anisole **5a** (108 mg, 1.00 mmol) was placed in the right chamber of an H-tube together with 2-MeTHF (108.1 µL), corresponding to η = 1 µL mg⁻¹ with respect to the substrate mass. In the left chamber, nitrogen dioxide was generated in situ from copper powder (95 mg, 1.50 mmol) and concentrated nitric acid (ca. 420 µL, 6.00 mmol, 65 wt%) according to the stoichiometry Cu + 4HNO₃ → Cu(NO₃)₂ + 2NO₂ + 2H₂O, corresponding to 3.00 equiv of NO₂ with respect to anisole **5a**. The H-tube was assembled and sealed, and the anisole-containing chamber was magnetically stirred at room temperature for 180 min, allowing gaseous NO₂ generated in the opposite chamber to diffuse into the stirred substrate-containing chamber. After completion, the reaction mixture was collected and analyzed by ¹H NMR spectroscopy. Only unreacted anisole **5a** was observed.

## Temperature Monitoring during the Reaction of 2,4,6-Tribromophenol

A series of independent reactions was performed to monitor the temperature of the reaction mixture over time. A 5 mL glass vial equipped with 3 mm glass milling balls was charged with 2,4,6-tribromophenol **3a** (331 mg, 1.00 mmol), NaNO₂ (207 mg, 3.00 mmol), and NaHSO₄ (360 mg, 3.00 mmol). After addition of 2-MeTHF as liquid-assisted grinding agent (0.898 mL, η = 1 μL mg⁻¹), the reaction mixture was subjected to mechanochemical activation in a Retsch AS 200 control apparatus at room temperature and 3 mm amplitude. Independent reactions were stopped after 5, 10, 20, 30, 50, 70, 100, 120, 150, and 180 min. At each time point, the mechanochemical activation was stopped, the vial was immediately opened, and a thermometer was inserted directly into the reaction mixture. The temperature inside the vial was 30 °C before addition of the reagents and remained 30 °C at *t* = 0, immediately before mechanochemical activation. Temperatures measured throughout the reaction ranged from 30 to 32 °C. These results indicate that the gentle mechanochemical mixing does not cause any appreciable heating of the reaction mixture due to friction or other mechanical effects.

**Graph S1.** Temperature profile during the gentle mechanochemical nitration of 2,4,6-tribromophenol **3a**. Independent reactions were stopped at the indicated time points, and the temperature was immediately measured by inserting a thermometer directly into the reaction mixture. The value at *t* = 0 corresponds to the temperature measured before addition of the reagents.

## ***Control experiment under conventional solution conditions***

A 25 mL round-bottom flask equipped with a magnetic stirring bar was charged with phenol **1a** (94.11 mg, 1.00 mmol), NaNO₂ (207.00 mg, 3.00 mmol), NaHSO₄ (360.18 mg, 3.00 mmol), and 2-MeTHF (10 mL). The resulting heterogeneous mixture was magnetically stirred at room temperature for 180 min. After completion, the reaction mixture was collected and analyzed by ¹H NMR spectroscopy. The analysis showed 20% conversion of **1a**, with formation of the corresponding nitrated products **2a** as the only detectable products, while the remaining 80% consisted of unreacted **1a**.

# Crystallographic data

**Figure S1**. Intermolecular C–H···O contacts in the crystal structure of **4o**, showing the centrosymmetric dimeric $R_{2}^{2}(10)$ motif generated by pairs of weak C7–H7···O3^i^ contacts involving the nitro group. C7···O3^i^ = 3.4611(15) Å, H7···O3^i^ = 2.6579(10) Å, C7–H7···O3^i^ = 145.02(2)°. All hydrogen atoms not involved in these contacts are omitted for clarity. Symmetry code: ^i^ = 1−*x*, 1−*y*, 1−*z*.

**Table S3**. Crystal data and structure refinement parameters for **4o**.

| CCDC deposition number | 2560685 |
| --- | --- |
| Empirical formula | C_15_H_23_NO_3_ |
| Formula weight | 265.34 |
| Temperature/K | 293(2) |
| Crystal system | Monoclinic |
| Space group | *P*2_1_/*n* |
| *a*/Å | 6.113(3) |
| *b*/Å | 22.318(11) |
| *c*/Å | 11.517(7) |
| *α*/° | 90 |
| *β*/° | 95.63(2) |
| *γ*/° | 90 |
| Volume/Å^3^ | 1563.7(14) |
| Z | 4 |
| ρ_calc_g/cm^3^ | 1.127 |
| μ/mm^−1^ | 0.078 |
| *F*(000) | 576.0 |
| Crystal size/mm^3^ | 0.36 × 0.07 × 0.06 |
| Radiation | MoKα (λ = 0.71073 Å) |
| 2θ range for data collection/° | 6.53 to 56.638 |
| Index ranges | −7 ≤ h ≤ 8, −29 ≤ k ≤ 29, −15 ≤ l ≤ 15 |
| Reflections collected | 22929 |
| Independent reflections | 3865 [R_int_ = 0.0661, R_sigma_ = 0.0427] |
| Data/restraints/parameters | 3865/0/179 |
| Goodness-of-fit on *F*^2^ | 1.058 |
| Final R indexes [I ≥ 2σ (I)] | R_1_ = 0.0620, wR_2_ = 0.1506 |
| Final R indexes [all data] | R_1_ = 0.1012, wR_2_ = 0.1744 |
| Largest diff. peak/hole / e Å^−3^ | 0.18/−0.22 |

**Table S4**. Bond lengths (Å) for **4o**.

| N1–O3 | 1.209(2) | C7–C6 | 1.330(3) |
| --- | --- | --- | --- |
| N1–O2 | 1.194(2) | C6–C5 | 1.497(3) |
| N1–C1 | 1.547(3) | C6–C12 | 1.542(3) |
| O1–C5 | 1.217(2) | C8–C9 | 1.529(3) |
| C1–C3 | 1.489(3) | C8–C10 | 1.533(3) |
| C1–C7 | 1.501(3) | C8–C11 | 1.532(3) |
| C1–C2 | 1.537(3) | C12–C14 | 1.539(3) |
| C3–C4 | 1.332(3) | C12–C13 | 1.531(3) |
| C4–C8 | 1.533(3) | C12–C15 | 1.533(3) |
| C4–C5 | 1.501(3) |  |  |

**Table S5**. Bond angles (°) for **4o**.

| O3–N1–C1 | 118.44(17) | C5–C6–C12 | 118.31(16) |
| --- | --- | --- | --- |
| O2–N1–O3 | 122.40(19) | C9–C8–C4 | 110.40(16) |
| O2–N1–C1 | 119.10(17) | C9–C8–C10 | 107.49(19) |
| C3–C1–N1 | 107.99(15) | C9–C8–C11 | 110.19(19) |
| C3–C1–C7 | 114.32(15) | C10–C8–C4 | 110.93(17) |
| C3–C1–C2 | 111.33(16) | C11–C8–C4 | 109.90(17) |
| C7–C1–N1 | 105.49(15) | C11–C8–C10 | 107.9(2) |
| C7–C1–C2 | 110.53(16) | O1–C5–C4 | 120.24(18) |
| C2–C1–N1 | 106.69(15) | O1–C5–C6 | 120.61(17) |
| C4–C3–C1 | 124.41(17) | C6–C5–C4 | 119.15(16) |
| C3–C4–C8 | 122.64(16) | C14–C12–C6 | 110.10(17) |
| C3–C4–C5 | 118.77(16) | C13–C12–C6 | 109.58(17) |
| C5–C4–C8 | 118.59(16) | C13–C12–C14 | 110.2(2) |
| C6–C7–C1 | 124.20(17) | C13–C12–C15 | 108.3(2) |
| C7–C6–C5 | 118.92(16) | C15–C12–C6 | 110.94(17) |
| C7–C6–C12 | 122.76(17) | C15–C12–C14 | 107.7(2) |

# DFT calculations

**Table S6.** Balanced chemical reactions c–k involved in Scheme 7 were adopted for thermochemical calculations. The corresponding *Δ*H and *Δ*G values are summarized in Table S10.

| **c** |  |
| --- | --- |
| **d** |  |
| **e** |  |
| **f** |  |
| **g** |  |
| **h** |  |
| **i** |  |
| **j** |  |
| **k** |  |

**Table S7.** Mulliken spin density calculated for the radical **II** at hybrid DFT-D3 level (M06-2X-D3/Def2-TZVP).

| **X** | **O** | **C(2)** | **C(4)** |
| --- | --- | --- | --- |
| H | 0.411 | 0.342 | 0.459 |
| F | 0.403 | 0.267 | 0.357 |
| Cl | 0.346 | 0.287 | 0.383 |
| Br | 0.342 | 0.290 | 0.380 |
| I | 0.336 | 0.291 | 0.383 |

**Table S8.** Sum of electronic and thermal energy E = E_0_ + E_tot_, sum of electronic and thermal enthalpies H = E_0_ + H_corr_, sum of electronic and thermal free energy G = E_0_ + G_corr_ calculated for 2,4,6-trisubstituted phenols (X = substituent) and related compounds at hybrid-DFT-D3 level (M06-2X-D3/Def2-TZVP). E_0_ = total electronic energy, E_tot_ = internal thermal energy contributions. All energies are given in Hartree. Optimized geometries for all compounds are reported in Tables S11–S52.

| **X** | **Species** | **E** | **H** | **G** |
| --- | --- | --- | --- | --- |
| **H** | Phenol | -307.3465 | -307.3456 | -307.3810 |
|  | phenyl nitrite | -436.6291 | -436.6282 | -436.6669 |
|  | phenoxyl radical | -306.7092 | -306.7082 | -306.7433 |
|  | 2-nitrocyclohexadienone | -511.8116 | -511.8107 | -511.8546 |
|  | 4-nitrocyclohexadienone | -511.8171 | -511.8161 | -511.8594 |
|  | 2-nitrophenol | -511.8627 | -511.8618 | -511.9029 |
|  | 4-nitrophenol | -511.8591 | -511.8581 | -511.9001 |
| **F** | 2,4,6-trifluorophenol | -605.1181 | -605.1171 | -605.1587 |
|  | 2,4,6-trifluorophenoxyl radical | -604.4856 | -604.4847 | -604.5259 |
|  | 2,4,6-trifluoro-2-nitrocyclohexadienone | -809.5767 | -809.5758 | -809.6249 |
|  | 2,4,6-trifluoro-4-nitrocyclohexadienone | -809.5797 | -809.5788 | -809.6286 |
|  | 2,4,6-trifluorophenyl nitrite | -734.3992 | -734.3982 | -734.4431 |
|  | 2-nitro-4,6-difluorophenol | -710.3718 | -710.3708 | -710.4161 |
|  | 4-nitro-2,6-difluorophenol | -710.3687 | -710.3678 | -710.4138 |
| **Cl** | 2,4,6-trichlorophenol | -1686.1788 | -1686.1778 | -1686.2229 |
|  | 2,4,6-trichlorophenoxyl radical | -1685.5433 | -1685.5424 | -1685.5874 |
|  | 2,4,6-trichloro-2-nitrocyclohexadienone | -1890.6300 | -1890.6291 | -1890.6824 |
|  | 2,4,6-trichloro-4-nitrocyclohexadienone | -1890.6341 | -1890.6332 | -1890.6868 |
|  | 2,4,6-trichlorophenyl nitrite | -1815.4568 | -1815.4558 | -1815.5043 |
|  | 2-nitro-4,6-dichlorophenol | -1431.0675 | -1431.0665 | -1431.1152 |
|  | 4-nitro-2,6-dichlorophenol | -1431.0772 | -1431.0762 | -1431.1245 |
| **Br** | 2,4,6-tribromophenol | -8028.1609 | -8028.1600 | -8028.2089 |
|  | 2,4,6-tribromophenoxyl radical | -8027.5243 | -8027.5233 | -8027.5723 |
|  | 2,4,6-tribromo-2-nitrocyclohexadienone | -8232.6130 | -8232.6120 | -8232.6690 |
|  | 2,4,6-tribromo-4-nitrocyclohexadienone | -8232.6182 | -8232.6173 | -8232.6743 |
|  | 2,4,6-tribromophenyl nitrite | -8157.4385 | -8157.4375 | -8157.4899 |
|  | 2-nitro-4,6-dibromophenol | -5659.0657 | -5659.0648 | -5659.1152 |
|  | 4-nitro-2,6-dibromophenol | -5659.0653 | -5659.0644 | -5659.1153 |
| **I** | 2,4,6-triiodophenol | -1198.4679 | -1198.4669 | -1198.5189 |
|  | 2,4,6-triiodophenoxyl radical | -1197.8318 | -1197.8309 | -1197.8829 |
|  | 2,4,6-triiodo-2-nitrocyclohexadienone | -1402.9236 | -1402.9227 | -1402.9824 |
|  | 2,4,6-triiodo-4-nitrocyclohexadienone | -1402.9300 | -1402.9290 | -1402.9887 |
|  | 2,4,6-triiodophenyl nitrite | -1327.7459 | -1327.7449 | -1327.8003 |
|  | 2-nitro-4,6-diiodophenol | -1105.9376 | -1105.9366 | -1105.9890 |
|  | 4-nitro-2,6-diiodophenol | -1105.9370 | -1105.9361 | -1105.9890 |
| ***^t^*Bu, Me** | bis(2,6-*tert*-butyl)-4-methylphenol | -660.8492 | -660.8483 | -660.9120 |
|  | bis(2,6-*tert*-butyl)-4-methylphenoxyl radical | -660.2279 | -660.2269 | -660.2908 |
|  | bis(2,6-*tert*-butyl)-4-methyl-2-nitrocyclohexadienone | -865.3297 | -865.3288 | -865.3962 |
|  | bis(2,6-*tert*-butyl)-4-methyl-4-nitrocyclohexadienone | -865.3360 | -865.3351 | -865.4044 |
|  | bis(2,6-*tert*-butyl)-4-methylphenyl nitrite | -790.1311 | -790.1301 | -790.1950 |
|  | 2-nitro- 4-methyl-6-*tert*-butylphenol | -708.2577 | -708.2568 | -708.3134 |
|  | 2,6-bis(*tert*-butyl)-4-nitrophenol | -826.0840 | -826.0830 | -826.1482 |

**Table S9.** Total electronic energy E_0_, sum of electronic and thermal enthalpies H = E_0_ + H_corr_, sum of electronic and thermal free energy G = E_0_ + G_corr_ calculated for inorganic species involved in the reactions summarized in Scheme 7 at hybrid-DFT-D3 level (M06-2X-D3/Def2-TZVP). All energies are given in Hartree.

| **Species** | **E_0_** | **H** | **G** |
| --- | --- | --- | --- |
| H_2_O | -76.4017 | -76.4008 | -76.4222 |
| NO_2_ | -205.0654 | -205.0644 | -205.0923 |
| NO | -129.8882 | -129.8873 | -129.9106 |
| HNO_2_ | -205.6857 | -205.6848 | -205.7128 |
| ^3^O_2_ | -150.3208 | -150.3199 | -150.3431 |
| F-O-N=O | -304.8164 | -304.8154 | -304.8464 |
| Cl-O-N=O | -665.2287 | -665.2277 | -665.2599 |
| Br-O-N=O | -2779.2353 | -2779.2343 | -2779.2678 |
| I-O-N=O | -502.6899 | -502.6889 | -502.7233 |

**Table S10**. Reaction enthalpies (ΔH, kcal/mol) and free energies (ΔG, kcal/mol) calculated for the nitration of 2,4,6-trisubstituted phenols (substituent = X; reactions c–k in Scheme 7 and Table S6) at hybrid DFT-D3 level (M06-2X-D3/Def2-TZVP).^a^

| **Reaction / X** | | **H** | **F** | **Cl** | **Br** | **I** | ***^t^*Bu** |
| --- | --- | --- | --- | --- | --- | --- | --- |
| **c** | ΔH | 0.88 | 2.36 | 4.36 | 4.06 | 4.37 | 1.07 |
|  | ΔG | 1.69 | 2.81 | 4.57 | 6.03 | 3.82 | 4.50 |
| **d** | ΔH | 20.50 | 15.95 | 15.84 | 16.87 | 16.20 | 9.99 |
|  | ΔG | 9.40 | 5.21 | 5.14 | 4.37 | 6.26 | –4.02 |
| **f** | ΔH | –23.84 | –16.73 | –13.98 | –15.22 | –17.16 | –23.46 |
|  | ΔG | –11.94 | –4.22 | –1.70 | –2.74 | –4.54 | –8.23 |
| **g** | ΔH | –27.26 | –18.63 | –16.57 | –18.51 | –21.15 | –27.44 |
|  | ΔG | –14.95 | –6.51 | –4.48 | –6.06 | –8.53 | –13.38 |
| **h** | ΔH | –32.07 | – | – | – | – | – |
|  | ΔG | –30.31 | – | – | – | – | – |
| **i** | ΔH | –26.37 | – | – | – | – | – |
|  | ΔG | –25.56 | – | – | – | – | – |
| **j** | ΔH | – | 46.64 | 12.31 | –1.42 | –11.36 | – |
|  | ΔG | – | 47.21 | 12.59 | –0.77 | –10.74 | – |
| **k** | ΔH | – | 50.43 | 8.82 | 1.80 | –7.03 | – |
|  | ΔG | – | 50.95 | 9.49 | 2.28 | –6.76 | – |

^a^ For reaction e, ΔH = –10.81 and ΔG = –6.38 kcal/mol.

**Table S11**. Molecular geometry optimized for phenol at DFT-D3 level in orthogonal Cartesian coordinate (Å) format. Most negative imaginary frequency: –9.67 cm^–1^.

| Atom | X | Y | Z |
| --- | --- | --- | --- |
| C | 0.01342 | -0.00054 | 0.00775 |
| C | 0.01689 | -0.00289 | 1.39608 |
| C | 1.21684 | -0.01323 | 2.09312 |
| C | 2.41604 | -0.02384 | 1.39490 |
| C | 2.42111 | -0.01323 | 0.00726 |
| C | 1.21749 | -0.00289 | -0.68341 |
| H | -0.92352 | 0.00944 | -0.53320 |
| H | -0.91843 | 0.00620 | 1.94092 |
| H | 1.24101 | -0.00305 | 3.17505 |
| H | 3.37018 | -0.00305 | -0.51278 |
| H | 1.22167 | 0.00620 | -1.76584 |
| O | 3.61160 | 0.01604 | 2.08516 |
| H | 3.91406 | -0.88024 | 2.25978 |

**Table S12**. Molecular geometry optimized for phenoxyl radical at DFT-D3 level in orthogonal Cartesian coordinate (Å) format. Most negative imaginary frequency: –13.78 cm^–1^.

| Atom | X | Y | Z |
| --- | --- | --- | --- |
| C | 0.02556 | 0.00000 | 0.01476 |
| C | 0.01470 | 0.00000 | 1.41838 |
| C | 1.19153 | 0.00000 | 2.11503 |
| C | 2.46364 | 0.00000 | 1.42238 |
| C | 2.42744 | 0.00000 | -0.02562 |
| C | 1.23570 | 0.00000 | -0.69646 |
| H | -0.91151 | 0.00000 | -0.52626 |
| H | -0.93194 | 0.00000 | 1.94301 |
| H | 1.22368 | 0.00000 | 3.19672 |
| H | 3.38028 | 0.00000 | -0.53863 |
| H | 1.21672 | 0.00000 | -1.77859 |
| O | 3.53974 | 0.00000 | 2.04367 |

**Table S13**. Molecular geometry optimized for phenyl nitrite at DFT-D3 level in orthogonal Cartesian coordinate (Å) format. Most negative imaginary frequency: –10.86 cm^–1^.

| Atom | X | Y | Z |
| --- | --- | --- | --- |
| C | 0.01749 | 0.01779 | 0.01010 |
| C | 0.01660 | 0.00377 | 1.39863 |
| C | 1.21527 | -0.02444 | 2.09666 |
| C | 2.40529 | -0.03715 | 1.38869 |
| C | 2.42339 | -0.02444 | 0.00412 |
| C | 1.21955 | 0.00377 | -0.68494 |
| H | -0.91919 | 0.04089 | -0.53069 |
| H | -0.91951 | 0.01438 | 1.94104 |
| H | 1.24228 | -0.03095 | 3.17824 |
| H | 3.37357 | -0.03095 | -0.51328 |
| H | 1.22123 | 0.01438 | -1.76684 |
| O | 3.60656 | -0.03260 | 2.08224 |
| N | 4.05467 | -1.35767 | 2.34095 |
| O | 5.05400 | -1.33992 | 2.91793 |

**Table S14**. Molecular geometry optimized for 2-nitrophenol at DFT-D3 level in orthogonal Cartesian coordinate (Å) format. Most negative imaginary frequency: –5.98 cm^–1^.

| Atom | X | Y | Z |
| --- | --- | --- | --- |
| C | -0.20877 | -1.90201 | 0.04264 |
| C | -0.03524 | -1.18692 | 1.23084 |
| C | 0.16930 | 0.17401 | 1.21732 |
| C | 0.20924 | 0.88094 | 0.01064 |
| C | 0.03330 | 0.14854 | -1.17389 |
| C | -0.17359 | -1.22990 | -1.15591 |
| H | -0.36885 | -2.97030 | 0.06215 |
| H | -0.06131 | -1.70740 | 2.17950 |
| H | 0.30497 | 0.73932 | 2.12904 |
| H | -0.30269 | -1.74051 | -2.09887 |
| O | 0.41109 | 2.19573 | 0.07358 |
| H | 0.41295 | 2.54255 | -0.83880 |
| N | 0.06146 | 0.80967 | -2.46913 |
| O | 0.24321 | 2.02437 | -2.49198 |
| O | -0.09291 | 0.14859 | -3.46305 |

**Table S15**. Molecular geometry optimized for 4-nitrophenol at DFT-D3 level in orthogonal Cartesian coordinate (Å) format. Most negative imaginary frequency: –10.79 cm^–1^.

| Atom | X | Y | Z |
| --- | --- | --- | --- |
| C | -0.01724 | -1.81282 | 0.12455 |
| C | 0.21667 | -1.05878 | 1.26020 |
| C | 0.27904 | 0.31775 | 1.14737 |
| C | 0.10671 | 0.92054 | -0.09668 |
| C | -0.12849 | 0.14535 | -1.23106 |
| C | -0.19078 | -1.22830 | -1.12077 |
| H | 0.34622 | -1.55278 | 2.21190 |
| H | 0.46137 | 0.92719 | 2.02442 |
| H | -0.25870 | 0.63970 | -2.18352 |
| H | -0.37123 | -1.85498 | -1.98183 |
| O | 0.15698 | 2.25994 | -0.26559 |
| H | 0.32436 | 2.69762 | 0.57530 |
| N | -0.08346 | -3.27334 | 0.24253 |
| O | -0.28929 | -3.90880 | -0.76718 |
| O | 0.07142 | -3.75573 | 1.34254 |

**Table S16.** Molecular geometry optimized for 2-nitrocyclohexadienone at DFT-D3 level in orthogonal Cartesian coordinate (Å) format. Most negative imaginary frequency: –12.51 cm^–1^.

| Atom | X | Y | Z |
| --- | --- | --- | --- |
| C | -1.78649 | -1.39415 | -0.12891 |
| C | -2.43833 | -0.09230 | -0.27457 |
| C | -1.81445 | 1.05922 | -0.00439 |
| C | -0.42575 | 1.06695 | 0.46739 |
| C | -0.52193 | -1.50349 | 0.26632 |
| H | -2.36211 | -2.28166 | -0.35615 |
| H | -3.46493 | -0.08016 | -0.61983 |
| H | -2.29097 | 2.02393 | -0.11374 |
| H | -0.02863 | -2.46222 | 0.36603 |
| O | 0.16573 | 2.06875 | 0.77628 |
| C | 0.28612 | -0.28749 | 0.59696 |
| H | 0.71576 | -0.35810 | 1.59632 |
| N | 1.48627 | -0.29235 | -0.33295 |
| O | 2.45337 | -0.90591 | 0.04276 |
| O | 1.36079 | 0.26844 | -1.39107 |

**Table S17**. Molecular geometry optimized for 4-nitrocyclohexadienone at DFT-D3 level in orthogonal Cartesian coordinate (Å) format. Most negative imaginary frequency: –9.40 cm^–1^.

| Atom | X | Y | Z |
| --- | --- | --- | --- |
| C | 1.58154 | -1.62251 | -0.01626 |
| C | 2.44324 | -0.45642 | 0.28815 |
| C | 1.80262 | 0.87743 | 0.22043 |
| C | 0.53373 | 1.02834 | -0.13991 |
| C | 0.31610 | -1.47924 | -0.39022 |
| H | 2.04232 | -2.59658 | 0.08551 |
| H | 2.42662 | 1.71894 | 0.49199 |
| H | -0.32115 | -2.32645 | -0.61016 |
| C | -0.30326 | -0.12824 | -0.56824 |
| H | -0.59753 | -0.01309 | -1.61734 |
| N | -1.65576 | -0.12515 | 0.14612 |
| O | -2.44093 | -0.96583 | -0.21629 |
| O | -1.84410 | 0.70236 | 0.99924 |
| O | 3.60574 | -0.58859 | 0.59205 |
| H | 0.06704 | 2.00433 | -0.16878 |

**Table S18**. Molecular geometry optimized for 2,4,6-trifluorophenol at DFT-D3 level in orthogonal Cartesian coordinate (Å) format. Most negative imaginary frequency: –3.69 cm^–1^.

| Atom | X | Y | Z |
| --- | --- | --- | --- |
| C | 0.03763 | 0.00987 | 0.02172 |
| C | 0.00349 | -0.00097 | 1.40229 |
| C | 1.21427 | -0.02040 | 2.06738 |
| C | 2.43737 | -0.03097 | 1.40722 |
| C | 2.39754 | -0.02040 | 0.01790 |
| C | 1.21616 | -0.00097 | -0.69812 |
| F | -1.11935 | 0.02918 | -0.64626 |
| H | -0.92990 | 0.00690 | 1.94536 |
| F | 1.22158 | -0.03533 | 3.39898 |
| F | 3.55439 | -0.03533 | -0.64157 |
| H | 1.21978 | 0.00690 | -1.77799 |
| O | 3.61738 | 0.03534 | 2.08849 |
| H | 3.94806 | -0.84892 | 2.27941 |

**Table S19**. Molecular geometry optimized for 2,4,6-trifluorophenoxyl radical at DFT-D3 level in orthogonal Cartesian coordinate (Å) format. Most negative imaginary frequency: –9.10 cm^–1^.

| Atom | X | Y | Z |
| --- | --- | --- | --- |
| C | 0.05203 | 0.00000 | 0.03004 |
| C | 0.00375 | 0.00000 | 1.42346 |
| C | 1.19232 | 0.00000 | 2.09452 |
| C | 2.48817 | 0.00000 | 1.43655 |
| C | 2.41007 | 0.00000 | -0.01468 |
| C | 1.23463 | 0.00000 | -0.70848 |
| F | -1.09567 | 0.00000 | -0.63258 |
| H | -0.94224 | 0.00000 | 1.94533 |
| F | 1.20619 | 0.00000 | 3.41185 |
| F | 3.55784 | 0.00000 | -0.66134 |
| H | 1.21358 | 0.00000 | -1.78867 |
| O | 3.55487 | 0.00000 | 2.05241 |

**Table S20**. Molecular geometry optimized for 2,4,6-trifluorophenyl nitrite at DFT-D3 level in orthogonal Cartesian coordinate (Å) format. Most negative imaginary frequency: –8.40 cm^–1^.

| Atom | X | Y | Z |
| --- | --- | --- | --- |
| C | 0.04061 | 0.02173 | 0.02345 |
| C | 0.00285 | 0.00616 | 1.40478 |
| C | 1.21170 | -0.02350 | 2.07074 |
| C | 2.43014 | -0.02835 | 1.40304 |
| C | 2.39916 | -0.02350 | 0.01399 |
| C | 1.21800 | 0.00616 | -0.69993 |
| F | -1.11479 | 0.04914 | -0.64362 |
| H | -0.93210 | 0.01365 | 1.94506 |
| F | 1.22576 | -0.04664 | 3.39962 |
| F | 3.55703 | -0.04664 | -0.63828 |
| H | 1.21842 | 0.01365 | -1.77975 |
| O | 3.61183 | -0.01110 | 2.08529 |
| N | 4.07624 | -1.37277 | 2.35341 |
| O | 5.06634 | -1.34111 | 2.92505 |

**Table S21**. Molecular geometry optimized for 2,4,6-trifluoro-2-nitrocyclohexadienone at DFT-D3 level in orthogonal Cartesian coordinate (Å) format. Most negative imaginary frequency: –9.69 cm^–1^.

| Atom | X | Y | Z |
| --- | --- | --- | --- |
| C | -1.73733 | -1.36488 | -0.13348 |
| C | -2.38984 | -0.08021 | -0.36267 |
| C | -1.75450 | 1.04489 | -0.05091 |
| C | -0.39784 | 1.04539 | 0.54469 |
| C | -0.51426 | -1.53271 | 0.34951 |
| F | -2.49469 | -2.41177 | -0.44208 |
| H | -3.39277 | -0.07151 | -0.76738 |
| F | -2.30463 | 2.23364 | -0.19157 |
| H | -0.07976 | -2.50911 | 0.51112 |
| O | 0.14504 | 2.02623 | 0.95148 |
| C | 0.30928 | -0.33279 | 0.64827 |
| F | 0.91567 | -0.42732 | 1.83956 |
| N | 1.43371 | -0.27167 | -0.41500 |
| O | 2.51687 | -0.66077 | -0.09847 |
| O | 1.07952 | 0.14203 | -1.49261 |

**Table S22**. Molecular geometry optimized for 2,4,6-trifluoro-4-nitrocyclohexadienone at DFT-D3 level in orthogonal Cartesian coordinate (Å) format. Most negative imaginary frequency: –8.14 cm^–1^.

| Atom | X | Y | Z |
| --- | --- | --- | --- |
| C | 1.53969 | -1.59451 | 0.04090 |
| C | 2.33928 | -0.40901 | 0.46624 |
| C | 1.69968 | 0.90595 | 0.17035 |
| C | 0.50041 | 1.02757 | -0.37347 |
| C | 0.33832 | -1.50606 | -0.50469 |
| F | 2.13836 | -2.75173 | 0.23673 |
| F | 2.43932 | 1.95131 | 0.48030 |
| H | -0.21670 | -2.38056 | -0.81921 |
| C | -0.31551 | -0.17973 | -0.68045 |
| F | -0.85646 | -0.08137 | -1.91164 |
| N | -1.54136 | -0.15254 | 0.30836 |
| O | -2.63027 | -0.05799 | -0.17726 |
| O | -1.25822 | -0.23082 | 1.47456 |
| O | 3.41652 | -0.50442 | 0.97827 |
| H | 0.06316 | 1.99320 | -0.59270 |

**Table S23**. Molecular geometry optimized for 2-nitro-4,6-difluorophenol at DFT-D3 level in orthogonal Cartesian coordinate (Å) format. Most negative imaginary frequency: –2.73 cm^–1^.

| Atom | X | Y | Z |
| --- | --- | --- | --- |
| C | -0.01185 | 0.17715 | 0.02040 |
| C | -0.02042 | 0.01535 | 1.40200 |
| C | 1.17420 | -0.17880 | 2.04383 |
| C | 2.40524 | -0.22188 | 1.36914 |
| C | 2.34575 | -0.05249 | -0.01752 |
| C | 1.14727 | 0.14742 | -0.70378 |
| F | -1.17736 | 0.36638 | -0.60347 |
| H | -0.94453 | 0.04115 | 1.96243 |
| F | 1.19656 | -0.33623 | 3.36286 |
| H | 1.14822 | 0.27352 | -1.77562 |
| O | 3.49760 | -0.41678 | 2.10135 |
| H | 4.26211 | -0.42169 | 1.49561 |
| N | 3.56954 | -0.08062 | -0.80927 |
| O | 4.62897 | -0.25603 | -0.21740 |
| O | 3.48845 | 0.06823 | -1.99924 |

**Table S24**. Molecular geometry optimized for 4-nitro-2,6-difluorophenol at DFT-D3 level in orthogonal Cartesian coordinate (Å) format. Most negative imaginary frequency: –8.25 cm^–1^.

| Atom | X | Y | Z |
| --- | --- | --- | --- |
| C | 0.09726 | -0.00067 | -0.08764 |
| C | -0.03088 | 0.16665 | 1.27996 |
| C | 1.11023 | 0.10202 | 2.04725 |
| C | 2.36434 | -0.12385 | 1.48470 |
| C | 2.42191 | -0.28338 | 0.10404 |
| C | 1.31351 | -0.22771 | -0.70590 |
| H | -0.99420 | 0.34265 | 1.73490 |
| F | 1.03766 | 0.25678 | 3.36538 |
| F | 3.63973 | -0.49984 | -0.41701 |
| H | 1.38954 | -0.35534 | -1.77523 |
| O | 3.44983 | -0.17924 | 2.26969 |
| H | 4.23201 | -0.34118 | 1.72737 |
| N | -1.11176 | 0.06556 | -0.92164 |
| O | -0.97321 | -0.08303 | -2.11387 |
| O | -2.16421 | 0.26412 | -0.36095 |

**Table S25**. Molecular geometry optimized for 2,4,6-trichlorophenol at DFT-D3 level in orthogonal Cartesian coordinate (Å) format. Most negative imaginary frequency: –8.26 cm^–1^.

| Atom | X | Y | Z |
| --- | --- | --- | --- |
| C | 0.02632 | 0.00918 | 0.01520 |
| C | 0.00945 | -0.00179 | 1.39932 |
| C | 1.21563 | -0.01901 | 2.08062 |
| C | 2.43605 | -0.02622 | 1.40645 |
| C | 2.40968 | -0.01901 | 0.01245 |
| C | 1.21657 | -0.00179 | -0.69148 |
| Cl | -1.47089 | 0.03382 | -0.84922 |
| H | -0.92354 | 0.00339 | 1.94328 |
| Cl | 1.21487 | -0.04490 | 3.80278 |
| Cl | 3.90074 | -0.04490 | -0.84928 |
| H | 1.22116 | 0.00339 | -1.77145 |
| O | 3.61244 | 0.04231 | 2.08565 |
| H | 3.94992 | -0.83955 | 2.28048 |

**Table S26**. Molecular geometry optimized for 2,4,6-trichlorophenoxyl radical at DFT-D3 level in orthogonal Cartesian coordinate (Å) format. Most negative imaginary frequency: –9.10 cm^–1^.

| Atom | X | Y | Z |
| --- | --- | --- | --- |
| C | 0.03838 | 0.00000 | 0.02216 |
| C | 0.00915 | 0.00000 | 1.42210 |
| C | 1.18904 | 0.00000 | 2.11107 |
| C | 2.49169 | 0.00000 | 1.43858 |
| C | 2.42276 | 0.00000 | -0.02579 |
| C | 1.23615 | 0.00000 | -0.70312 |
| Cl | -1.44475 | 0.00000 | -0.83413 |
| H | -0.93699 | 0.00000 | 1.94373 |
| Cl | 1.20038 | 0.00000 | 3.81469 |
| Cl | 3.90381 | 0.00000 | -0.86778 |
| H | 1.21482 | 0.00000 | -1.78333 |
| O | 3.55108 | 0.00000 | 2.05022 |

**Table S27**. Molecular geometry optimized for 2,4,6-trichlorophenyl nitrite at DFT-D3 level in orthogonal Cartesian coordinate (Å) format. Most negative imaginary frequency: –8.40 cm^–1^.

| Atom | X | Y | Z |
| --- | --- | --- | --- |
| C | 0.02782 | 0.02337 | 0.01606 |
| C | 0.00873 | 0.00535 | 1.40081 |
| C | 1.21293 | -0.02552 | 2.08331 |
| C | 2.42844 | -0.02936 | 1.40206 |
| C | 2.41066 | -0.02552 | 0.00877 |
| C | 1.21750 | 0.00535 | -0.69285 |
| Cl | -1.46668 | 0.06504 | -0.84679 |
| H | -0.92534 | 0.01252 | 1.94299 |
| Cl | 1.21672 | -0.05837 | 3.80396 |
| Cl | 3.90269 | -0.05837 | -0.84828 |
| H | 1.22001 | 0.01252 | -1.77286 |
| O | 3.60699 | -0.00871 | 2.08249 |
| N | 4.08096 | -1.37120 | 2.35614 |
| O | 5.06975 | -1.33024 | 2.92702 |

**Table S28**. Molecular geometry optimized for 2,4,6-trichloro-2-nitrocyclohexadienone at DFT-D3 level in orthogonal Cartesian coordinate (Å) format. Most negative imaginary frequency: –9.69 cm^–1^.

| Atom | X | Y | Z |
| --- | --- | --- | --- |
| C | -1.74963 | -1.38055 | -0.17584 |
| C | -2.43713 | -0.09352 | -0.23650 |
| C | -1.79985 | 1.05140 | 0.02369 |
| C | -0.35685 | 1.06881 | 0.35856 |
| C | -0.47737 | -1.51436 | 0.18136 |
| Cl | -2.70219 | -2.75610 | -0.58169 |
| H | -3.48474 | -0.09018 | -0.50568 |
| Cl | -2.57511 | 2.57069 | -0.04973 |
| H | 0.01353 | -2.47608 | 0.24494 |
| O | 0.28999 | 2.06300 | 0.48796 |
| C | 0.31276 | -0.31300 | 0.56943 |
| Cl | 0.78365 | -0.42664 | 2.25893 |
| N | 1.56872 | -0.28969 | -0.33437 |
| O | 2.63103 | -0.57511 | 0.12952 |
| O | 1.31763 | -0.00921 | -1.48013 |

**Table S29**. Molecular geometry optimized for 2,4,6-trichloro-4-nitrocyclohexadienone at DFT-D3 level in orthogonal Cartesian coordinate (Å) format. Most negative imaginary frequency: –8.14 cm^–1^.

| Atom | X | Y | Z |
| --- | --- | --- | --- |
| C | 1.53757 | -1.63909 | -0.03830 |
| C | 2.41250 | -0.48983 | 0.35462 |
| C | 1.76563 | 0.85766 | 0.27214 |
| C | 0.51071 | 1.02539 | -0.12714 |
| C | 0.28215 | -1.47846 | -0.43869 |
| Cl | 2.25988 | -3.18539 | 0.05962 |
| Cl | 2.74930 | 2.17847 | 0.72643 |
| H | -0.34106 | -2.31579 | -0.71794 |
| C | -0.32700 | -0.12698 | -0.55227 |
| Cl | -0.85953 | 0.13195 | -2.24250 |
| N | -1.66544 | -0.10456 | 0.24825 |
| O | -2.23090 | -1.15614 | 0.37669 |
| O | -2.03544 | 0.96699 | 0.64427 |
| O | 3.54480 | -0.63764 | 0.71169 |
| H | 0.05304 | 2.00271 | -0.18059 |

**Table S30**. Molecular geometry optimized for 2-nitro-4,6-dichlorophenol at DFT-D3 level in orthogonal Cartesian coordinate (Å) format. Most negative imaginary frequency: –2.73 cm^–1^.

| Atom | X | Y | Z |
| --- | --- | --- | --- |
| C | 0.01889 | 0.11390 | -0.00228 |
| C | 0.09130 | -0.04955 | 1.37246 |
| C | 1.32661 | -0.19361 | 1.97177 |
| C | 2.52026 | -0.19567 | 1.23863 |
| C | 2.39430 | -0.03299 | -0.14214 |
| C | 1.17002 | 0.13432 | -0.76404 |
| Cl | -1.52163 | 0.29531 | -0.76062 |
| H | -0.80567 | -0.05948 | 1.97528 |
| Cl | 1.43245 | -0.37057 | 3.68913 |
| H | 1.13695 | 0.27659 | -1.83421 |
| O | 3.71752 | -0.30603 | 1.81751 |
| H | 3.60622 | -0.35922 | 2.77660 |
| N | 3.58352 | -0.03776 | -1.01068 |
| O | 4.51789 | -0.72150 | -0.67803 |
| O | 3.52114 | 0.63096 | -2.01803 |

**Table S31**. Molecular geometry optimized for 4-nitro-2,6-dichlorophenol at DFT-D3 level in orthogonal Cartesian coordinate (Å) format. Most negative imaginary frequency: –11.32 cm^–1^.

| Atom | X | Y | Z |
| --- | --- | --- | --- |
| C | 0.09256 | -0.00773 | -0.12901 |
| C | 0.03955 | 0.06188 | 1.24929 |
| C | 1.22900 | -0.00707 | 1.94451 |
| C | 2.45823 | -0.14330 | 1.29104 |
| C | 2.46047 | -0.20958 | -0.10712 |
| C | 1.28183 | -0.14239 | -0.82197 |
| H | -0.90598 | 0.16697 | 1.75980 |
| Cl | 1.23447 | 0.07420 | 3.67300 |
| Cl | 3.96156 | -0.37696 | -0.92542 |
| H | 1.28359 | -0.19348 | -1.90049 |
| O | 3.62309 | -0.21242 | 1.93950 |
| H | 3.47089 | -0.15427 | 2.89272 |
| N | -1.16513 | 0.06432 | -0.88723 |
| O | -1.09272 | 0.00145 | -2.09237 |
| O | -2.18966 | 0.18189 | -0.25519 |

**Table S32**. Molecular geometry optimized for 2,4,6-tribromophenol at DFT-D3 level in orthogonal Cartesian coordinate (Å) format. Most negative imaginary frequency: –0.01 cm^–1^.

| Atom | X | Y | Z |
| --- | --- | --- | --- |
| C | 0.02597 | 0.00956 | 0.01499 |
| C | 0.00883 | -0.00164 | 1.39947 |
| C | 1.21573 | -0.01858 | 2.07948 |
| C | 2.43699 | -0.02500 | 1.40700 |
| C | 2.40875 | -0.01858 | 0.01312 |
| C | 1.21639 | -0.00164 | -0.69209 |
| Br | -1.60934 | 0.03720 | -0.92916 |
| H | -0.92392 | 0.00302 | 1.94392 |
| Br | 1.21244 | -0.04870 | 3.96578 |
| Br | 4.04068 | -0.04870 | -0.93289 |
| H | 1.22152 | 0.00302 | -1.77209 |
| O | 3.61269 | 0.04319 | 2.08579 |
| H | 3.95165 | -0.83824 | 2.28149 |

**Table S33**. Molecular geometry optimized for 2,4,6-tribromophenoxyl radical at DFT-D3 level in orthogonal Cartesian coordinate (Å) format. Most negative imaginary frequency: –7.41 cm^–1^.

| Atom | X | Y | Z |
| --- | --- | --- | --- |
| C | 0.03774 | 0.00000 | 0.02179 |
| C | 0.00937 | 0.00000 | 1.42212 |
| C | 1.18947 | 0.00000 | 2.11016 |
| C | 2.49197 | 0.00000 | 1.43874 |
| C | 2.42219 | 0.00000 | -0.02497 |
| C | 1.23628 | 0.00000 | -0.70295 |
| Br | -1.58317 | 0.00000 | -0.91404 |
| H | -0.93680 | 0.00000 | 1.94401 |
| Br | 1.19825 | 0.00000 | 3.97738 |
| Br | 4.04364 | 0.00000 | -0.95098 |
| H | 1.21516 | 0.00000 | -1.78330 |
| O | 3.55145 | 0.00000 | 2.05043 |

**Table S34**. Molecular geometry optimized for 2,4,6-tribromophenyl nitrite at DFT-D3 level in orthogonal Cartesian coordinate (Å) format. Most negative imaginary frequency: –17.12 cm^–1^, corresponding to the rotation of the nitrite moiety.

| Atom | X | Y | Z |
| --- | --- | --- | --- |
| C | 0.02771 | 0.02370 | 0.01600 |
| C | 0.00763 | 0.00587 | 1.40124 |
| C | 1.21316 | -0.02395 | 2.08255 |
| C | 2.42970 | -0.02621 | 1.40279 |
| C | 2.41012 | -0.02395 | 0.00935 |
| C | 1.21732 | 0.00587 | -0.69402 |
| Br | -1.60628 | 0.06578 | -0.92738 |
| H | -0.92629 | 0.01219 | 1.94337 |
| Br | 1.21285 | -0.06616 | 3.96705 |
| Br | 4.04199 | -0.06616 | -0.93317 |
| H | 1.21986 | 0.01219 | -1.77388 |
| O | 3.60766 | -0.00376 | 2.08288 |
| N | 4.08397 | -1.37063 | 2.35787 |
| O | 5.07180 | -1.32790 | 2.92820 |

**Table S35**. Molecular geometry optimized for 2,4,6-tribromo-2-nitrocyclohexadienone at DFT-D3 level in orthogonal Cartesian coordinate (Å) format. Most negative imaginary frequency: –5.40 cm^–1^.

| Atom | X | Y | Z |
| --- | --- | --- | --- |
| C | -1.76275 | -1.37131 | -0.18921 |
| C | -2.45608 | -0.08739 | -0.15020 |
| C | -1.79724 | 1.05347 | 0.07784 |
| C | -0.32642 | 1.06546 | 0.25023 |
| C | -0.47004 | -1.50723 | 0.08766 |
| Br | -2.81329 | -2.86893 | -0.63774 |
| H | -3.52312 | -0.07969 | -0.32593 |
| Br | -2.65014 | 2.71757 | 0.08922 |
| H | 0.03465 | -2.46385 | 0.08085 |
| O | 0.35215 | 2.04742 | 0.22851 |
| C | 0.31840 | -0.31527 | 0.49701 |
| Br | 0.66034 | -0.40835 | 2.40057 |
| N | 1.63480 | -0.31393 | -0.29919 |
| O | 2.67761 | -0.45479 | 0.26573 |
| O | 1.45559 | -0.18374 | -1.48489 |

**Table S36**. Molecular geometry optimized for 2,4,6-tribromo-4-nitrocyclohexadienone at DFT-D3 level in orthogonal Cartesian coordinate (Å) format. Most negative imaginary frequency: –2.79 cm^–1^.

| Atom | X | Y | Z |
| --- | --- | --- | --- |
| C | 1.54356 | -1.63237 | -0.03144 |
| C | 2.42533 | -0.47604 | 0.31870 |
| C | 1.75936 | 0.86230 | 0.26928 |
| C | 0.49089 | 1.02005 | -0.09163 |
| C | 0.27434 | -1.48341 | -0.39350 |
| Br | 2.33703 | -3.32543 | 0.07061 |
| Br | 2.82589 | 2.32417 | 0.75154 |
| H | -0.35724 | -2.32535 | -0.63793 |
| C | -0.33769 | -0.13693 | -0.51161 |
| Br | -0.86932 | 0.13551 | -2.38832 |
| N | -1.69259 | -0.11077 | 0.24302 |
| O | -2.25929 | -1.16264 | 0.36687 |
| O | -2.07501 | 0.96223 | 0.62422 |
| O | 3.57488 | -0.61232 | 0.62409 |
| H | 0.01609 | 1.99029 | -0.11762 |

**Table S37**. Molecular geometry optimized for 2-nitro-4,6-dibromophenol at DFT-D3 level in orthogonal Cartesian coordinate (Å) format. Most negative imaginary frequency: –8.90 cm^–1^.

| Atom | X | Y | Z |
| --- | --- | --- | --- |
| C | 0.00598 | 0.15423 | -0.00557 |
| C | 0.01518 | 0.01117 | 1.38090 |
| C | 1.20705 | -0.16440 | 2.04756 |
| C | 2.43510 | -0.20529 | 1.36438 |
| C | 2.37677 | -0.05686 | -0.02963 |
| C | 1.17984 | 0.12169 | -0.71694 |
| Br | -1.63809 | 0.39366 | -0.90063 |
| H | -0.91068 | 0.03731 | 1.93813 |
| Br | 1.21598 | -0.35623 | 3.91759 |
| H | 1.20093 | 0.23010 | -1.79072 |
| O | 3.54493 | -0.37757 | 2.06673 |
| H | 4.29670 | -0.38144 | 1.44149 |
| N | 3.60020 | -0.08514 | -0.82637 |
| O | 4.66523 | -0.24212 | -0.23789 |
| O | 3.51463 | 0.04556 | -2.01771 |

**Table S38**. Molecular geometry optimized for 4-nitro-2,6-dibromophenol at DFT-D3 level in orthogonal Cartesian coordinate (Å) format. Most negative imaginary frequency: –0.01 cm^–1^.

| Atom | X | Y | Z |
| --- | --- | --- | --- |
| C | 0.06465 | -0.00160 | -0.10475 |
| C | -0.05655 | 0.12498 | 1.26742 |
| C | 1.08450 | 0.06321 | 2.04037 |
| C | 2.34370 | -0.12291 | 1.45682 |
| C | 2.41153 | -0.24632 | 0.06433 |
| C | 1.28351 | -0.18709 | -0.72751 |
| H | -1.02849 | 0.26881 | 1.71522 |
| Br | 0.96468 | 0.22939 | 3.90993 |
| Br | 4.10895 | -0.50033 | -0.73260 |
| H | 1.33805 | -0.28231 | -1.80162 |
| O | 3.41460 | -0.17477 | 2.25076 |
| H | 4.21411 | -0.30665 | 1.72182 |
| N | -1.14813 | 0.06335 | -0.93371 |
| O | -1.01077 | -0.05053 | -2.12997 |
| O | -2.20258 | 0.22629 | -0.36545 |

**Table S39**. Molecular geometry optimized for 2,4,6-triiodophenol at DFT-D3 level in orthogonal Cartesian coordinate (Å) format. Most negative imaginary frequency: –7.25 cm^–1^.

| Atom | X | Y | Z |
| --- | --- | --- | --- |
| C | 0.02301 | 0.00786 | 0.01328 |
| C | 0.01014 | -0.00205 | 1.39926 |
| C | 1.21669 | -0.01687 | 2.08293 |
| C | 2.43570 | -0.02424 | 1.40625 |
| C | 2.41221 | -0.01687 | 0.01222 |
| C | 1.21686 | -0.00205 | -0.69085 |
| I | -1.78286 | 0.03563 | -1.02934 |
| H | -0.92289 | 0.00279 | 1.94351 |
| I | 1.21030 | -0.04435 | 4.16779 |
| I | 4.21456 | -0.04435 | -1.03574 |
| H | 1.22168 | 0.00279 | -1.77100 |
| O | 3.61364 | 0.04000 | 2.08634 |
| H | 3.94935 | -0.84338 | 2.28016 |

**Table S40**. Molecular geometry optimized for 2,4,6-triiodophenoxyl radical at DFT-D3 level in orthogonal Cartesian coordinate (Å) format. Most negative imaginary frequency: –8.05 cm^–1^.

| Atom | X | Y | Z |
| --- | --- | --- | --- |
| C | 0.03321 | 0.00000 | 0.01917 |
| C | 0.00937 | 0.00000 | 1.42171 |
| C | 1.18904 | 0.00000 | 2.11304 |
| C | 2.48997 | 0.00000 | 1.43758 |
| C | 2.42452 | 0.00000 | -0.02670 |
| C | 1.23592 | 0.00000 | -0.70275 |
| I | -1.76081 | 0.00000 | -1.01660 |
| H | -0.93603 | 0.00000 | 1.94511 |
| I | 1.20270 | 0.00000 | 4.17879 |
| I | 4.22029 | 0.00000 | -1.04783 |
| H | 1.21650 | 0.00000 | -1.78318 |
| O | 3.55078 | 0.00000 | 2.05004 |

**Table S41**. Molecular geometry optimized for 2,4,6-triiodophenyl nitrite at DFT-D3 level in orthogonal Cartesian coordinate (Å) format. Most negative imaginary frequency: –6.01 cm^–1^.

| Atom | X | Y | Z |
| --- | --- | --- | --- |
| C | 0.02502 | 0.01972 | 0.01445 |
| C | 0.01007 | 0.00488 | 1.40102 |
| C | 1.21496 | -0.02101 | 2.08665 |
| C | 2.42898 | -0.02544 | 1.40237 |
| C | 2.41457 | -0.02101 | 0.00886 |
| C | 1.21835 | 0.00488 | -0.69179 |
| I | -1.77977 | 0.05927 | -1.02755 |
| H | -0.92385 | 0.01153 | 1.94360 |
| I | 1.20600 | -0.05390 | 4.17069 |
| I | 4.21493 | -0.05390 | -1.04093 |
| H | 1.22128 | 0.01153 | -1.77188 |
| O | 3.60955 | -0.00651 | 2.08397 |
| N | 4.08145 | -1.37544 | 2.35642 |
| O | 5.06965 | -1.33774 | 2.92696 |

**Table S42**. Molecular geometry optimized for 2,4,6-triiodo-2-nitrocyclohexadienone at DFT-D3 level in orthogonal Cartesian coordinate (Å) format. Most negative imaginary frequency: –2.57 cm^–1^.

| Atom | X | Y | Z |
| --- | --- | --- | --- |
| C | -1.76929 | -1.37108 | -0.20664 |
| C | -2.45664 | -0.08731 | -0.09636 |
| C | -1.78336 | 1.05181 | 0.11142 |
| C | -0.30255 | 1.05286 | 0.16528 |
| C | -0.46388 | -1.50178 | 0.02237 |
| I | -2.92965 | -3.02358 | -0.71214 |
| H | -3.53291 | -0.07492 | -0.20567 |
| I | -2.71325 | 2.89972 | 0.19925 |
| H | 0.05121 | -2.45097 | -0.03711 |
| O | 0.38409 | 2.02099 | 0.02033 |
| C | 0.32808 | -0.32033 | 0.44299 |
| I | 0.57549 | -0.38166 | 2.58253 |
| N | 1.68034 | -0.32830 | -0.27409 |
| O | 2.69624 | -0.24707 | 0.35159 |
| O | 1.57051 | -0.40894 | -1.47331 |

**Table S43**. Molecular geometry optimized for 2,4,6-triiodo-4-nitrocyclohexadienone at DFT-D3 level in orthogonal Cartesian coordinate (Å) format. Most negative imaginary frequency: –7.38 cm^–1^.

| Atom | X | Y | Z |
| --- | --- | --- | --- |
| C | 1.54196 | -1.63257 | -0.01832 |
| C | 2.42939 | -0.46951 | 0.28391 |
| C | 1.75502 | 0.86334 | 0.27848 |
| C | 0.47278 | 1.01252 | -0.04402 |
| C | 0.25940 | -1.48580 | -0.34074 |
| I | 2.42275 | -3.50745 | 0.08661 |
| I | 2.93494 | 2.48832 | 0.80044 |
| H | -0.38454 | -2.32859 | -0.54771 |
| C | -0.35067 | -0.14259 | -0.46883 |
| I | -0.85388 | 0.15005 | -2.57767 |
| N | -1.72638 | -0.10757 | 0.22574 |
| O | -2.30374 | -1.15635 | 0.33596 |
| O | -2.12128 | 0.96787 | 0.59030 |
| O | 3.59710 | -0.59832 | 0.52836 |
| H | -0.01664 | 1.97594 | -0.03624 |

**Table S44**. Molecular geometry optimized for 2-nitro-4,6-diiodophenol at DFT-D3 level in orthogonal Cartesian coordinate (Å) format. Most negative imaginary frequency: –5.28 cm^–1^.

| Atom | X | Y | Z |
| --- | --- | --- | --- |
| C | 0.01341 | 0.14918 | -0.01621 |
| C | 0.02654 | 0.01264 | 1.37290 |
| C | 1.21782 | -0.15894 | 2.04561 |
| C | 2.44391 | -0.20018 | 1.36045 |
| C | 2.38861 | -0.05863 | -0.03507 |
| C | 1.19135 | 0.11427 | -0.72345 |
| I | -1.80065 | 0.40605 | -1.01101 |
| H | -0.90000 | 0.04044 | 1.92969 |
| I | 1.22314 | -0.36246 | 4.11471 |
| H | 1.22076 | 0.21644 | -1.79779 |
| O | 3.55812 | -0.36711 | 2.05875 |
| H | 4.30797 | -0.37202 | 1.43037 |
| N | 3.61250 | -0.08908 | -0.83142 |
| O | 4.67843 | -0.24097 | -0.24251 |
| O | 3.52785 | 0.03506 | -2.02368 |

**Table S45**. Molecular geometry optimized for 4-nitro-2,6-diiodophenol at DFT-D3 level in orthogonal Cartesian coordinate (Å) format. Most negative imaginary frequency: –7.02 cm^–1^.

| Atom | X | Y | Z |
| --- | --- | --- | --- |
| C | 0.07415 | -0.00687 | -0.14233 |
| C | 0.01957 | 0.05059 | 1.23670 |
| C | 1.20534 | -0.01460 | 1.94231 |
| C | 2.43741 | -0.14110 | 1.28574 |
| C | 2.44470 | -0.19736 | -0.11430 |
| C | 1.26713 | -0.12888 | -0.83147 |
| H | -0.93506 | 0.14649 | 1.73189 |
| I | 1.16144 | 0.07904 | 4.02727 |
| I | 4.25877 | -0.38889 | -1.11678 |
| H | 1.26006 | -0.16989 | -1.91053 |
| O | 3.60914 | -0.21092 | 1.92367 |
| H | 3.47865 | -0.16060 | 2.88136 |
| N | -1.18245 | 0.06421 | -0.90242 |
| O | -1.10733 | 0.01418 | -2.10811 |
| O | -2.20975 | 0.16811 | -0.27195 |

**Table S46**. Molecular geometry optimized for bis(2,6-tert-butyl)-4-methylphenol at DFT-D3 level in orthogonal Cartesian coordinate (Å) format. Most negative imaginary frequency: –5.31 cm^–1^.

| Atom | X | Y | Z |
| --- | --- | --- | --- |
| C | 0.01554 | 0.02528 | 0.00011 |
| C | 0.04675 | 0.00160 | 1.38289 |
| C | 1.24006 | -0.02773 | 2.10578 |
| C | 2.43512 | -0.03261 | 1.37095 |
| C | 2.45280 | -0.00927 | -0.03594 |
| C | 1.22511 | 0.01931 | -0.68393 |
| H | -0.89721 | 0.00636 | 1.90884 |
| H | 1.19267 | 0.03789 | -1.76471 |
| O | 3.65634 | -0.06059 | 1.99038 |
| H | 3.54038 | -0.07486 | 2.94109 |
| C | 1.22760 | -0.05337 | 3.64306 |
| C | 1.87509 | -1.34628 | 4.17762 |
| H | 2.90915 | -1.50573 | 3.87124 |
| H | 1.85306 | -1.34557 | 5.26931 |
| H | 1.31025 | -2.21067 | 3.82563 |
| C | 1.91175 | 1.20170 | 4.22041 |
| H | 1.88917 | 1.16497 | 5.31146 |
| H | 2.95012 | 1.34152 | 3.91907 |
| H | 1.37219 | 2.09331 | 3.89790 |
| C | -0.20108 | -0.04216 | 4.19915 |
| H | -0.74214 | 0.85697 | 3.90199 |
| H | -0.76765 | -0.91484 | 3.87220 |
| H | -0.15746 | -0.06111 | 5.28919 |
| C | 3.77044 | -0.01510 | -0.82167 |
| C | 4.56940 | -1.29324 | -0.51924 |
| H | 5.48454 | -1.30057 | -1.11586 |
| H | 4.84271 | -1.36242 | 0.53011 |
| H | 3.98495 | -2.17628 | -0.78575 |
| C | 4.60573 | 1.22867 | -0.47702 |
| H | 4.88042 | 1.25479 | 0.57393 |
| H | 5.52099 | 1.22959 | -1.07350 |
| H | 4.04709 | 2.13659 | -0.71355 |
| C | 3.52451 | 0.01375 | -2.33337 |
| H | 2.96307 | -0.85937 | -2.67068 |
| H | 2.98865 | 0.91351 | -2.64100 |
| H | 4.48724 | 0.00845 | -2.84653 |
| C | -1.28598 | 0.05687 | -0.75464 |
| H | -2.13608 | 0.05671 | -0.07326 |
| H | -1.35657 | 0.94865 | -1.38021 |
| H | -1.38120 | -0.80982 | -1.41142 |

**Table S47**. Molecular geometry optimized for bis(2,6-tert-butyl)-4-methylphenoxyl radical at DFT-D3 level in orthogonal Cartesian coordinate (Å) format. Most negative imaginary frequency: –4.69 cm^–1^.

| Atom | X | Y | Z |
| --- | --- | --- | --- |
| C | -0.00577 | 2.26912 | 0.00007 |
| C | 1.20930 | 1.57142 | 0.00004 |
| C | 1.26734 | 0.19964 | 0.00000 |
| C | 0.00448 | -0.55258 | -0.00001 |
| C | -1.26691 | 0.18942 | 0.00005 |
| C | -1.21937 | 1.55683 | 0.00008 |
| H | 2.11971 | 2.15504 | 0.00004 |
| H | -2.13391 | 2.13542 | 0.00013 |
| O | 0.00992 | -1.79336 | -0.00006 |
| C | 2.58788 | -0.55898 | -0.00005 |
| C | 2.68738 | -1.43772 | 1.25915 |
| H | 1.90000 | -2.18622 | 1.28172 |
| H | 3.65500 | -1.94413 | 1.26818 |
| H | 2.61894 | -0.82442 | 2.16025 |
| C | 2.68757 | -1.43727 | -1.25955 |
| H | 3.65521 | -1.94364 | -1.26864 |
| H | 1.90022 | -2.18579 | -1.28248 |
| H | 2.61921 | -0.82365 | -2.16043 |
| C | 3.78666 | 0.39277 | 0.00021 |
| H | 3.80064 | 1.02932 | -0.88677 |
| H | 3.80057 | 1.02893 | 0.88747 |
| H | 4.70468 | -0.19609 | 0.00012 |
| C | -2.58044 | -0.58193 | 0.00006 |
| C | -2.67219 | -1.46093 | 1.25942 |
| H | -3.63503 | -1.97636 | 1.26865 |
| H | -1.87804 | -2.20223 | 1.28267 |
| H | -2.60955 | -0.84658 | 2.16023 |
| C | -2.67206 | -1.46115 | -1.25915 |
| H | -1.87787 | -2.20242 | -1.28222 |
| H | -3.63488 | -1.97663 | -1.26836 |
| H | -2.60938 | -0.84696 | -2.16007 |
| C | -3.78736 | 0.35927 | -0.00008 |
| H | -3.80660 | 0.99566 | 0.88694 |
| H | -3.80656 | 0.99544 | -0.88725 |
| H | -4.70042 | -0.23729 | -0.00003 |
| C | -0.03199 | 3.76570 | -0.00003 |
| H | 0.97208 | 4.18556 | 0.00095 |
| H | -0.56249 | 4.14074 | -0.87834 |
| H | -0.56433 | 4.14087 | 0.87710 |

**Table S48**. Molecular geometry optimized for bis(2,6-tert-butyl)-4-methylphenyl nitrite at DFT-D3 level in orthogonal Cartesian coordinate (Å) format. Most negative imaginary frequency: –28.14 cm^–1^, corresponding to the rotation of the methyl in the 4-position.

| Atom | X | Y | Z |
| --- | --- | --- | --- |
| C | 0.02190 | 0.09989 | 0.01158 |
| C | 0.03449 | 0.04852 | 1.39755 |
| C | 1.21195 | -0.10634 | 2.12535 |
| C | 2.39153 | -0.24073 | 1.38124 |
| C | 2.44734 | -0.10844 | -0.01256 |
| C | 1.22897 | 0.04641 | -0.66952 |
| H | -0.91096 | 0.13034 | 1.91378 |
| H | 1.20420 | 0.12671 | -1.74656 |
| O | 3.57900 | -0.49988 | 2.06777 |
| N | 3.79033 | -1.90026 | 2.19043 |
| O | 4.77447 | -2.11203 | 2.75900 |
| C | 1.20440 | -0.03254 | 3.66111 |
| C | 2.02933 | 1.18897 | 4.09802 |
| H | 2.03022 | 1.26046 | 5.18812 |
| H | 3.06153 | 1.11379 | 3.75979 |
| H | 1.59714 | 2.10693 | 3.69548 |
| C | 1.77526 | -1.29722 | 4.32759 |
| H | 2.86188 | -1.29578 | 4.34090 |
| H | 1.44546 | -1.33591 | 5.36740 |
| H | 1.42894 | -2.20464 | 3.82987 |
| C | -0.21813 | 0.15718 | 4.19978 |
| H | -0.68554 | 1.06187 | 3.80921 |
| H | -0.85728 | -0.69678 | 3.96663 |
| H | -0.17124 | 0.25195 | 5.28505 |
| C | 3.77414 | -0.03698 | -0.78623 |
| C | 4.63534 | -1.30236 | -0.62291 |
| H | 5.37106 | -1.34313 | -1.42823 |
| H | 5.19003 | -1.30010 | 0.31155 |
| H | 4.03016 | -2.20920 | -0.67263 |
| C | 4.56600 | 1.18446 | -0.29170 |
| H | 4.78880 | 1.11062 | 0.77150 |
| H | 5.51074 | 1.25419 | -0.83579 |
| H | 4.00226 | 2.10271 | -0.46627 |
| C | 3.53029 | 0.15085 | -2.28790 |
| H | 3.00772 | -0.70298 | -2.72380 |
| H | 2.95979 | 1.05608 | -2.49904 |
| H | 4.49397 | 0.24343 | -2.78965 |
| C | -1.27187 | 0.27335 | -0.73631 |
| H | -2.10316 | -0.17672 | -0.19434 |
| H | -1.50081 | 1.33265 | -0.87258 |
| H | -1.21929 | -0.18249 | -1.72464 |

**Table S49**. Molecular geometry optimized for 2,6-bis(tert-butyl)-4-nitrophenol at DFT-D3 level in orthogonal Cartesian coordinate (Å) format. Most negative imaginary frequency: –3.60 cm^–1^.

| Atom | X | Y | Z |
| --- | --- | --- | --- |
| C | 0.09736 | 0.03139 | -0.03095 |
| C | 0.06008 | -0.00183 | 1.34640 |
| C | 1.24175 | -0.03419 | 2.07336 |
| C | 2.44836 | -0.03121 | 1.34291 |
| C | 2.49498 | 0.00264 | -0.06635 |
| C | 1.28320 | 0.03402 | -0.73799 |
| H | -0.90455 | -0.00166 | 1.82667 |
| H | 1.23275 | 0.06094 | -1.81426 |
| O | 3.64830 | -0.06149 | 1.97576 |
| H | 3.52313 | -0.08305 | 2.92655 |
| N | -1.17024 | 0.06513 | -0.77053 |
| O | -2.19869 | 0.06213 | -0.12910 |
| O | -1.11710 | 0.09406 | -1.98029 |
| C | 1.21289 | -0.07142 | 3.60945 |
| C | 3.82372 | 0.00495 | -0.83165 |
| C | 4.61927 | -1.27471 | -0.52709 |
| H | 5.53825 | -1.27701 | -1.11687 |
| H | 4.88752 | -1.34960 | 0.52324 |
| H | 4.03929 | -2.15748 | -0.80328 |
| C | 4.65035 | 1.24823 | -0.46547 |
| H | 4.91966 | 1.26517 | 0.58712 |
| H | 5.56951 | 1.25667 | -1.05492 |
| H | 4.09248 | 2.15745 | -0.69797 |
| C | 3.58967 | 0.04477 | -2.34470 |
| H | 3.03303 | -0.82668 | -2.69392 |
| H | 3.05487 | 0.94567 | -2.65066 |
| H | 4.55632 | 0.04519 | -2.84949 |
| C | 1.85681 | -1.36722 | 4.14083 |
| H | 2.90074 | -1.51546 | 3.86232 |
| H | 1.80744 | -1.37814 | 5.23104 |
| H | 1.30761 | -2.23209 | 3.76646 |
| C | 1.88826 | 1.18057 | 4.20321 |
| H | 1.83838 | 1.13942 | 5.29268 |
| H | 2.93575 | 1.31642 | 3.93183 |
| H | 1.36087 | 2.07600 | 3.87181 |
| C | -0.22574 | -0.06660 | 4.13841 |
| H | -0.76330 | 0.83365 | 3.83830 |
| H | -0.78513 | -0.93761 | 3.79503 |
| H | -0.20220 | -0.09352 | 5.22841 |

**Table S50**. Molecular geometry optimized for 2-nitro-4-methyl-6-tert-butylphenol at DFT-D3 level in orthogonal Cartesian coordinate (Å) format. Most negative imaginary frequency: –1.12 cm^–1^.

| Atom | X | Y | Z |
| --- | --- | --- | --- |
| C | -0.00513 | 0.05210 | -0.05323 |
| C | 0.03288 | 0.03388 | 1.34976 |
| C | 1.19402 | -0.03183 | 2.09630 |
| C | 2.42041 | -0.08381 | 1.38861 |
| C | 2.38367 | -0.06557 | -0.01323 |
| C | 1.18750 | 0.00148 | -0.72817 |
| H | -0.91692 | 0.07447 | 1.86628 |
| H | 1.23072 | 0.01206 | -1.80763 |
| O | 3.56112 | -0.14762 | 2.08004 |
| H | 4.29485 | -0.17588 | 1.43011 |
| N | 3.61166 | -0.11645 | -0.79129 |
| O | 4.68573 | -0.17545 | -0.19159 |
| O | 3.54035 | -0.09950 | -1.99282 |
| C | 1.17621 | -0.04874 | 3.62789 |
| C | 1.80942 | -1.34867 | 4.15015 |
| H | 2.85113 | -1.44090 | 3.85474 |
| H | 1.75899 | -1.36208 | 5.24082 |
| H | 1.26167 | -2.21593 | 3.77604 |
| C | 1.93283 | 1.17070 | 4.17930 |
| H | 1.88240 | 1.16396 | 5.27003 |
| H | 2.97898 | 1.16730 | 3.88517 |
| H | 1.47310 | 2.09574 | 3.82565 |
| C | -0.25337 | 0.01500 | 4.17160 |
| H | -0.76357 | 0.93168 | 3.86974 |
| H | -0.85003 | -0.84087 | 3.85037 |
| H | -0.21483 | 0.00116 | 5.26134 |
| C | -1.32271 | 0.12528 | -0.77259 |
| H | -1.95443 | -0.72713 | -0.51706 |
| H | -1.86723 | 1.03027 | -0.49789 |
| H | -1.18058 | 0.13002 | -1.85190 |

**Table S51**. Molecular geometry optimized for bis(2,6-tert-butyl)-4-methyl-2-nitrocyclohexadienone at DFT-D3 level in orthogonal Cartesian coordinate (Å) format. Most negative imaginary frequency: –2.21 cm^–1^.

| Atom | X | Y | Z |
| --- | --- | --- | --- |
| C | -1.65360 | -1.43101 | -0.23191 |
| C | -2.34375 | -0.13856 | -0.22559 |
| C | -1.75130 | 1.04843 | -0.02252 |
| C | -0.27990 | 1.05070 | 0.19585 |
| C | -0.36271 | -1.50435 | 0.07932 |
| H | -3.40849 | -0.17752 | -0.42286 |
| H | 0.16761 | -2.44903 | 0.06889 |
| O | 0.37801 | 2.06104 | 0.21115 |
| C | 0.43180 | -0.29403 | 0.46841 |
| N | 1.61866 | -0.33028 | -0.50929 |
| O | 2.61966 | -0.91542 | -0.17374 |
| O | 1.43003 | 0.15639 | -1.59495 |
| C | -2.48651 | 2.38180 | -0.05783 |
| C | -2.25431 | 3.13805 | 1.26009 |
| H | -1.19923 | 3.35077 | 1.42007 |
| H | -2.79324 | 4.08701 | 1.23190 |
| H | -2.63371 | 2.56101 | 2.10667 |
| C | -1.98523 | 3.22523 | -1.24223 |
| H | -2.13709 | 2.69148 | -2.18229 |
| H | -2.55310 | 4.15706 | -1.28477 |
| H | -0.92978 | 3.46490 | -1.14465 |
| C | -3.99294 | 2.17907 | -0.22570 |
| H | -4.23393 | 1.70085 | -1.17706 |
| H | -4.41366 | 1.57941 | 0.58438 |
| H | -4.48537 | 3.15199 | -0.21178 |
| C | -2.46771 | -2.63437 | -0.60070 |
| H | -3.30618 | -2.75613 | 0.08867 |
| H | -2.88762 | -2.51560 | -1.60182 |
| H | -1.86729 | -3.54164 | -0.58171 |
| C | 0.87313 | -0.34985 | 1.98225 |
| C | -0.35512 | 0.04119 | 2.82053 |
| H | -0.13316 | -0.14486 | 3.87214 |
| H | -0.59935 | 1.09964 | 2.71548 |
| H | -1.23213 | -0.55096 | 2.55057 |
| C | 2.01037 | 0.62720 | 2.29985 |
| H | 1.77998 | 1.64104 | 1.97946 |
| H | 2.15215 | 0.63594 | 3.38233 |
| H | 2.94544 | 0.31595 | 1.83915 |
| C | 1.29119 | -1.76438 | 2.40425 |
| H | 1.65553 | -1.71807 | 3.43168 |
| H | 0.44595 | -2.45375 | 2.38642 |
| H | 2.08918 | -2.15887 | 1.77940 |

**Table S52**. Molecular geometry optimized for bis(2,6-tert-butyl)-4-methyl-4-nitrocyclohexadienone at DFT-D3 level in orthogonal Cartesian coordinate (Å) format. Most negative imaginary frequency: –4.49 cm^–1^.

| Atom | X | Y | Z |
| --- | --- | --- | --- |
| C | 1.57440 | -1.62657 | -0.00071 |
| C | 2.41186 | -0.40891 | 0.27183 |
| C | 1.76949 | 0.94644 | 0.20147 |
| C | 0.51056 | 1.03634 | -0.22526 |
| C | 0.32423 | -1.45953 | -0.42948 |
| H | -0.32241 | -2.29830 | -0.64991 |
| C | -0.28914 | -0.12526 | -0.69713 |
| N | -1.65473 | -0.12073 | 0.03110 |
| O | -2.41895 | -1.00791 | -0.26160 |
| O | -1.87579 | 0.75977 | 0.82313 |
| O | 3.58546 | -0.52107 | 0.55165 |
| H | 0.00910 | 1.99144 | -0.29725 |
| C | 2.58804 | 2.16452 | 0.61959 |
| C | 3.79816 | 2.33536 | -0.31269 |
| H | 4.34431 | 3.23758 | -0.02972 |
| H | 4.47545 | 1.48780 | -0.25161 |
| H | 3.47008 | 2.45214 | -1.34794 |
| C | 3.05980 | 1.99997 | 2.07363 |
| H | 3.72679 | 1.14931 | 2.18674 |
| H | 3.59464 | 2.90060 | 2.38121 |
| H | 2.20515 | 1.87133 | 2.74111 |
| C | 1.75130 | 3.44315 | 0.54320 |
| H | 1.42324 | 3.65346 | -0.47685 |
| H | 0.87298 | 3.39131 | 1.18976 |
| H | 2.36097 | 4.28482 | 0.87332 |
| C | 2.19391 | -3.00426 | 0.21545 |
| C | 2.66971 | -3.13959 | 1.67105 |
| H | 3.06089 | -4.14663 | 1.82797 |
| H | 3.45488 | -2.42574 | 1.90569 |
| H | 1.83747 | -2.99038 | 2.36221 |
| C | 3.37470 | -3.20810 | -0.74759 |
| H | 4.17033 | -2.49113 | -0.56445 |
| H | 3.77621 | -4.21474 | -0.61419 |
| H | 3.04378 | -3.11296 | -1.78407 |
| C | 1.17535 | -4.11486 | -0.04858 |
| H | 0.30774 | -4.03452 | 0.60935 |
| H | 0.82965 | -4.11173 | -1.08422 |
| H | 1.64805 | -5.07950 | 0.13864 |
| C | -0.62557 | 0.02151 | -2.18799 |
| H | -1.25511 | -0.80472 | -2.51216 |
| H | -1.14349 | 0.96410 | -2.36872 |
| H | 0.30605 | 0.01452 | -2.75146 |

**Figure S2.** Correlation between structural C–C, C–O, C–N, and N–O bond distances determined by single crystal X-ray diffraction (SC-XRD) and the corresponding metric parameters optimized at hybrid DFT-D3 level for compound **4o**. The dashed line represents the least-squares linear correlation (slope 0.990, intercept 0.014 Å, dispersion coefficient R^2^ = 0.998).

**Figure S3.** Electron spin density for radical II (X = I; <S^2^> = 0.75) calculated at the UM06-2X-D3/Def2-TZVP level. Contour value = 0.005 a.u.

**Figure S4.** Kohn-Sham LUMO (left; cutoff value = 0.05 |e|) and molecular electrostatic potential (MEP) map (right; red = –0.430, blue = +0.430 a.u.) mapped onto the 4·10^–4^ a.u. isodensity surface calculated for 2,4,6-triiodo-4-nitrocyclohexadienone at the optimized geometry at M06-2X-D3/Def2-TZVP level.

# Raman Analysis

**Isolation of Molecular Iodine Formed during the Nitration of Triiodophenol**

A 5 mL glass vial equipped with glass milling balls (3 mm diameter) was charged with 2,4,6-triiodophenol (**3n**, 1.00 mmol), NaNO₂ (3.00 mmol), and NaHSO₄ (3.00 mmol). 2-MeTHF was added as liquid-assisted grinding agent (η = 1), and the vial was subjected to mechanochemical activation in a Retsch AS200 apparatus for 180 min at room temperature and an amplitude of 3 mm/G. After completion of the reaction, the crude mixture was dissolved in ethyl acetate and filtered through paper. The solvent was removed under reduced pressure, and the resulting crude residue was purified by chromatography/filtration through a short silica gel column using hexane as eluent. A violet-colored solution was collected from the hexane fraction. The solvent was removed under reduced pressure, and the obtained solid was purified by sublimation using a sublimator. The Raman spectrum of the isolated byproduct shows a single intense band which matches the characteristic I–I stretching vibration of molecular iodine (I₂). The absence of additional peaks in both the low-frequency (50–250 cm⁻¹) and fingerprint (300–1800 cm⁻¹) regions confirms that the byproduct is pure I₂ without detectable organic contaminants.

**Figure S5.** Raman spectra of the byproduct recovered after the nitration of 2,4,6-triiodophenol (**3n**) (black) compared with pure commercial iodine from Sigma-Aldrich (red). The spectra were normalized for clarity.

# Characterization Data

## Products 2a–2ac

### 2-nitrophenol **2a′**

The title compound was synthesized according to the general procedure A stated above. Phenol (94.11 mg, 1.00 mmol), 2-MeTHF (661 µL), NaNO₂ (207.00 mg, 3.00 mmol), and NaHSO₄ (360.18 mg, 3.00 mmol) were used, to afford **2a′** as a yellow solid (66.77 mg, 0.48 mmol, 48%).

**^1^H NMR (600 MHz, CDCl₃):** *δ* 8.11 (dt, *J =* 8.5, 1.6 Hz, 1H), 7.58 (ddd, *J =* 8.5, 7.2, 1.6 Hz, 1H), 7.16 (dt, *J =* 8.5, 1.1 Hz, 1H), 6.99 (ddt, *J =* 8.5, 7.2, 1.1 Hz, 1H).

**^13^C NMR (151 MHz, CDCl₃):** *δ* 155.3, 137.7, 133.9, 125.2, 120.4, 120.1.

The spectroscopic data closely match the ones previously reported in the literature.^[16]^

### 4-nitrophenol **2a′′**

The title compound was synthesized according to the general procedure A stated above. Phenol (94.11 mg, 1.00 mmol), 2-MeTHF (661 µL), NaNO₂ (207.00 mg, 3.00 mmol), and NaHSO₄ (360.18 mg, 3.00 mmol) were used, to afford **2a′′** as a white solid (65.38 mg, 0.47 mmol, 47%).

**^1^H NMR (600 MHz, CDCl₃):** *δ* 10.11 (s, 1H), 8.10 (dd, *J =* 9.0, 1.5 Hz, 2H), 6.92 (dd, *J =* 9.0, 1.5 Hz, 2H).

**^13^C NMR (151 MHz, CDCl₃):** *δ* 163.9, 139.6, 126.2, 115.8.

The spectroscopic data closely match the ones previously reported in the literature.^[17]^

### 2,4-dimethyl-6-nitrophenol **2b**

The title compound was independently synthesized according to General Procedures A and B.

General Procedure A: 2,4-Dimethylphenol (122.17 mg, 1.00 mmol), 2-MeTHF (690 μL), NaNO₂ (207.00 mg, 3.00 mmol), and NaHSO₄ (360.18 mg, 3.00 mmol) were used, affording **2b** as a yellow solid (150.44 mg, 0.90 mmol, 90%).

General Procedure B: 2,4-Dimethyl-6-bromophenol (201.06 mg, 1.00 mmol), 2-MeTHF (768 μL), NaNO₂ (207.00 mg, 3.00 mmol), and NaHSO₄ (360.18 mg, 3.00 mmol) were used, affording **2b** as a yellow solid (147.10 mg, 0.88 mmol, 88%).

**^1^H NMR (600 MHz, CDCl₃):** *δ* 10.74 (s, 1H), 7.72 (s, 1H), 7.26 (s, 1H), 2.29 (s, 6H).

**^13^C NMR (151 MHz, CDCl₃):** *δ* 151.9, 142.2, 139.6, 133.1, 129.1, 121.9, 20.4, 15.8.

The spectroscopic data closely match the ones previously reported in the literature.^[24]^

### 2-(*tert*-butyl)-4-methyl-6-nitrophenol **2c**

The title compound was independently synthesized according to General Procedures A and B.

General Procedure A: 2-(*tert*-Butyl)-4-methylphenol (164.25 mg, 1.00 mmol), 2-MeTHF (731 μL), NaNO₂ (207.00 mg, 3.00 mmol), and NaHSO₄ (360.18 mg, 3.00 mmol) were used, affording **2c** as a yellow solid (177.86 mg, 0.85 mmol, 85%).

General Procedure B: 6-Bromo-2-(*tert-*butyl)-4-methylphenol (243.14 mg, 1.00 mmol), 2-MeTHF (811 μL), NaNO₂ (207.00 mg, 3.00 mmol), and NaHSO₄ (360.18 mg, 3.00 mmol) were used, affording **2c** as a yellow solid (169.49 mg, 0.81 mmol, 81%).

**^1^H NMR (600 MHz, CDCl₃):** *δ* 11.41 (s, 1H), 7.80 – 7.77 (m, 1H), 7.38 (m, 1H), 2.32 (s, 3H), 1.43 (s, 9H).

**^13^C NMR (151 MHz, CDCl₃):** *δ* 153.2, 140.3, 136.2, 134.0, 128.7, 122.3, 35.6, 29.5, 20.8.

The spectroscopic data closely match the ones previously reported in the literature.^[25]^

### 2,6-di-*tert*-butyl-4-nitrophenol **2d**

The title compound was independently synthesized according to General Procedures A and B.

General Procedure A: 2,6-Di-tert-butylphenol (206.33 mg, 1.00 mmol), 2-MeTHF (774 μL), NaNO₂ (207.00 mg, 3.00 mmol), and NaHSO₄ (360.18 mg, 3.00 mmol) were used, affording **2d** as a yellow solid (158.88 mg, 0.63 mmol, 63%).

General Procedure B: 4-Bromo-2,6-di-tert-butylphenol (285.23 mg, 1.00 mmol), 2-MeTHF (853 μL), NaNO₂ (207.00 mg, 3.00 mmol), and NaHSO₄ (360.18 mg, 3.00 mmol) were used, affording **2d** as a yellow solid (164.98 mg, 0.66 mmol, 66%).

**^1^H NMR (600 MHz, CDCl₃):** *δ* 8.12 (s, 2H), 5.92 (s, 1H), 1.48 (s, 18H).

**^13^C NMR (151 MHz, CDCl₃):** *δ* 159.7, 140.9, 136.8, 121.5, 34.7, 30.1.

The spectroscopic data closely match the ones previously reported in the literature.^[26]^

### 4-ethyl-2-nitrophenol **2e**

The title compound was synthesized according to the general procedure A stated above. 4-ethyl-phenol (122.17 mg, 1.00 mmol), 2-MeTHF (689 µL), NaNO₂ (207.00 mg, 3.00 mmol), and NaHSO₄ (360.18 mg, 3.00 mmol) were used, to afford **2e** as a yellow solid (137.07 mg, 0.82 mmol, 82%).

**^1^H NMR (600 MHz, CDCl₃):** *δ* 10.43 (s, 1H), 7.88 (d, *J =* 2.3 Hz, 1H), 7.41 (dd, *J =* 8.6, 2.3 Hz, 1H), 7.05 (d, *J =* 8.6 Hz, 1H), 2.63 (q, *J =* 7.6 Hz, 2H), 1.23 (t, *J =* 7.6 Hz, 3H).

**^13^C NMR (151 MHz, CDCl₃):** *δ* 153.4, 137.8, 136.6, 133.4, 123.3, 119.8, 27.7, 15.3.

The spectroscopic data closely match the ones previously reported in the literature.^[19]^

### 2-nitro-4-(*tert*-pentyl)phenol **2f**

The title compound was synthesized according to the general procedure A stated above. 4-(*tert-*pentyl)phenol (164.25 mg, 1.00 mmol), 2-MeTHF (732 µL), NaNO₂ (207.00 mg, 3.00 mmol), and NaHSO₄ (360.18 mg, 3.00 mmol) were used, to afford **2f** as a yellow solid (117.18 mg, 0.56 mmol, 56%).

**^1^H NMR (600 MHz, CDCl₃):** *δ* 10.46 (s, 1H), 8.01 (d, *J =* 2.4 Hz, 1H), 7.57 (dd, *J =* 8.9, 2.4 Hz, 1H), 7.09 (d, *J =* 8.9 Hz, 1H), 1.63 (q, *J =* 7.4 Hz, 2H), 1.31 – 1.26 (m, 6H), 0.68 (t, *J =* 7.4 Hz, 3H).

**^13^C NMR (151 MHz, CDCl₃):** *δ* 153.1, 142.2, 136.0, 133.2, 121.8, 119.6, 37.7, 36.6, 28.3, 9.1.

**HRMS:** calculated for C₁₁H₁₄NO₃⁻: 208.0974 [M−H]⁻; found: 208.0964.

### 4-(*tert*-butyl)-2-nitrophenol **2g**

The title compound was synthesized according to the general procedure A stated above. 4-(*tert*-butyl)-phenol (150.22 mg, 1.00 mmol), 2-MeTHF (717 µL), NaNO₂ (207.00 mg, 3.00 mmol), and NaHSO₄ (360.18 mg, 3.00 mmol) were used, to afford **2g** as a yellow solid (163.98 mg, 0.84 mmol, 84%).

**^1^H NMR (600 MHz, CDCl₃):** *δ* 10.45 (s, 1H), 8.06 (d, *J =* 2.5 Hz, 1H), 7.63 (dd, *J =* 8.8, 2.5 Hz, 1H), 7.09 (d, *J =* 8.8 Hz, 1H), 1.32 (s, 9H).

**^13^C NMR (151 MHz, CDCl₃):** *δ* 153.2, 143.9, 135.6, 133.2, 121.0, 119.7, 34.5, 31.1.

The spectroscopic data closely match the ones previously reported in the literature.^[19]^

### 4-isopropyl-2-nitrophenol **2h**

The title compound was synthesized according to the general procedure A stated above. 4-(isopropyl)-phenol (136.19 mg, 1.00 mmol), 2-MeTHF (703 µL), NaNO₂ (207.00 mg, 3.00 mmol), and NaHSO₄ (360.18 mg, 3.00 mmol) were used, to afford **2h** as a yellow solid (134.08 mg, 0.74 mmol, 74%).

**^1^H NMR (600 MHz, CDCl₃):** *δ* 10.44 (s, 1H), 7.91 (d, *J =* 2.4 Hz, 1H), 7.46 (dd, *J =* 8.7, 2.4 Hz, 1H), 7.07 (d, *J =* 8.7 Hz, 1H), 2.90 (hept, *J =* 6.9 Hz, 1H), 1.24 (d, *J =* 6.9 Hz, 6H).

**^13^C NMR (151 MHz, CDCl₃):** *δ* 153.4, 141.1, 136.5, 133.4, 122.0, 119.6, 33.2, 23.8.

The spectroscopic data closely match the ones previously reported in the literature.^[19]^

### 4-methyl-2-nitrophenol **2i**

The title compound was synthesized according to the general procedure A stated above *4-methylphenol* (108.14 mg, 1.00 mmol), 2-MeTHF (675 µL), NaNO₂ (207.00 mg, 3.00 mmol), and NaHSO₄ (360.18 mg, 3.00 mmol) were used, to afford **2i** as a yellow solid (136.29 mg, 0.89 mmol, 89%).

**^1^H NMR (600 MHz, CDCl₃):** *δ* 10.42 (s, 1H), 7.88 (d, *J =* 2.2 Hz, 1H), 7.39 (dd, *J =* 8.6, 2.2 Hz, 1H), 7.05 (d, *J =* 8.6 Hz, 1H), 2.34 (s, 3H).

**^13^C NMR (151 MHz, CDCl₃):** *δ* 153.3, 138.9, 133.4, 130.2, 124.5, 119.8, 20.4.

The spectroscopic data closely match the ones previously reported in the literature.^[19]^

### 2-allyl-6-nitrophenol **2j′**

The title compound was synthesized according to the general procedure A stated above 2-allylphenol (134.18 mg, 1.00 mmol), 2-MeTHF (701 µL), NaNO₂ (207.00 mg, 3.00 mmol), and NaHSO₄ (360.18 mg, 3.00 mmol) were used, to afford **2j′** as a yellow solid (66.30 mg, 0.37 mmol, 37%).

**^1^H NMR (600 MHz, CDCl₃):** *δ* 10.94 (m, 1H), 8.00 (m, 1H), 7.46 (m, 1H), 6.93 (m, 1H), 5.99 (m, 1H), 5.12 (m, 2H), 3.49 (d, *J =* 6.7 Hz, 2H).

**^13^C NMR (151 MHz, CDCl₃):** *δ* 153.4, 137.7, 135.3, 131.6, 123.3, 119.6, 117.0, 33.8.

The spectroscopic data closely match the ones previously reported in the literature.^[22]^

### 2-allyl-4-nitrophenol **2j′′**

The title compound was synthesized according to the general procedure A stated above 2-allylphenol (134.18 mg, 1.00 mmol), 2-MeTHF (701 µL), NaNO₂ (207.00 mg, 3.00 mmol), and NaHSO₄ (360.18 mg, 3.00 mmol) were used, to afford **2j′′** as a yellow solid (68.09 mg, 0.38 mmol, 38%).

**^1^H NMR (600 MHz, CDCl₃):** *δ* 8.13 – 8.01 (m, 2H), 6.89 (d, *J =* 8.5 Hz, 1H), 6.08 (s, 1H), 6.00 (ddt, *J =* 16.7, 10.1, 6.5 Hz, 1H), 5.28 – 5.18 (m, 2H), 3.47 (d, *J =* 6.5 Hz, 2H).

**^13^C NMR (151 MHz, CDCl₃):** *δ* 159.9, 141.7, 134.7, 126.7, 126.5, 124.4, 118.1, 115.9, 34.7.

The spectroscopic data closely match the ones previously reported in the literature.^[23]^

### 2-methyl-6-nitrophenol **2k′**

The title compound was synthesized according to the general procedure A stated above. 2-methyl-phenol (108.14 mg, 1.00 mmol), 2-MeTHF (675 µL), NaNO₂ (207.00 mg, 3.00 mmol), and NaHSO₄ (360.18 mg, 3.00 mmol) were used, to afford **2k′** as a yellow solid (70.44 mg, 0.46 mmol, 46%).

**^1^H NMR (600 MHz, CDCl₃):** *δ* 10.90 (s, 1H), 7.96 (d, *J =* 8.6 Hz, 1H), 7.48 – 7.40 (m, 1H), 6.87 (d, *J =* 8.6 Hz, 1H), 2.34 (s, 3H).

**^13^C NMR (151 MHz, CDCl₃):** *δ* 153.8, 138.1, 133.5, 129.5, 122.6, 119.3, 15.8.

The spectroscopic data closely match the ones previously reported in the literature.^[18]^

### 2-methyl-4-nitrophenol **2k′′**

The title compound was synthesized according to the general procedure A stated above. 2-methyl-phenol (108.14 mg, 1.00 mmol), 2-MeTHF (675 µL), NaNO₂ (207.00 mg, 3.00 mmol), and NaHSO₄ (360.18 mg, 3.00 mmol) were used, to afford **2k′′** as a yellow solid (61.26 mg, 0.40 mmol, 40%).

**^1^H NMR (600 MHz, CDCl₃):** *δ* 8.06 (d, *J =* 2.7 Hz, 1H), 8.01 (dd, *J =* 8.8, 2.7 Hz, 1H), 6.85 (d, *J =* 8.8 Hz, 1H), 5.90 (s, 1H), 2.32 (s, 3H).

**^13^C NMR (151 MHz, CDCl₃):** *δ* 159.8, 141.5, 127.0, 125.2, 123.8, 115.0, 15.9.

The spectroscopic data closely match the ones previously reported in the literature.^[18]^

### 2-isopropyl-6-nitrophenol **2l′**

The title compound was synthesized according to the general procedure A stated above 2-isopropylphenol (136.19 mg, 1.00 mmol), 2-MeTHF (703 µL), NaNO₂ (207.00 mg, 3.00 mmol), and NaHSO₄ (360.18 mg, 3.00 mmol) were used, to afford **2l′** as a yellow solid (86.97 mg, 0.48 mmol, 48%).

**^1^H NMR (600 MHz, CDCl₃):** *δ* 11.06 (s, 1H), 7.96 (dd, *J =* 8.5, 1.7 Hz, 1H), 7.51 (dd, *J =* 7.4, 1.7 Hz, 1H), 6.93 (dd, *J =* 8.5, 7.4 Hz, 1H), 3.43 (hept, *J =* 7.0 Hz, 1H), 1.27 (d, *J =* 7.0 Hz, 6H).

**^13^C NMR (151 MHz, CDCl₃):** *δ* 153.2, 139.8, 134.2, 133.7, 122.6, 119.7, 27.1, 22.4.

**HRMS:** calculated for C₉H₁₀NO₃⁻: 180.0661 [M−H]⁻; found: 180.0659.

### 2-isopropyl-4-nitrophenol **2l′′**

The title compound was synthesized according to the general procedure A stated above 2-isopropylphenol (136.19 mg, 1.00 mmol), 2-MeTHF (703 µL), NaNO₂ (207.00 mg, 3.00 mmol), and NaHSO₄ (360.18 mg, 3.00 mmol) were used, to afford **2l′′** as a yellow solid (77.91 mg, 0.43 mmol, 43%).

**^1^H NMR (600 MHz, CDCl₃):** *δ* 8.13 (d, *J =* 2.7 Hz, 1H), 8.00 (dd, *J =* 8.8, 2.7 Hz, 1H), 6.82 (d, *J =* 8.8 Hz, 1H), 5.89 (s, 1H), 3.26 (h, *J =* 6.9 Hz, 1H), 1.29 (d, *J =* 6.9 Hz, 6H).

**^13^C NMR (151 MHz, CDCl₃):** *δ* 158.7, 142.0, 135.8, 123.4, 123.1, 115.4, 27.3, 22.3.

The spectroscopic data closely match the ones previously reported in the literature.^[21]^

### 2-ethyl-6-nitrophenol **2m′**

The title compound was synthesized according to the general procedure A stated above 2-ethylphenol (122.17 mg, 1.00 mmol), 2-MeTHF (689 µL), NaNO₂ (207.00 mg, 3.00 mmol), and NaHSO₄ (360.18 mg, 3.00 mmol) were used, to afford **2m′** as a yellow solid (75.22 mg, 0.45 mmol, 45%).

**^1^H NMR (600 MHz, CDCl₃):** *δ* 10.94 (s, 1H), 7.96 (dd, *J =* 8.5, 1.7 Hz, 1H), 7.46 (td, *J =* 7.4, 1.7 Hz, 1H), 6.91 (dd, *J =* 8.5, 7.5 Hz, 1H), 2.76 (q, *J =* 7.5 Hz, 2H), 1.25 (t, *J =* 7.5 Hz, 3H).

**^13^C NMR (151 MHz, CDCl₃):** *δ* 153.6, 136.7, 135.4, 133.7, 122.7, 119.5, 23.0, 13.7.

HRMS: calculated for C₈H₈NO₃⁻: 166.0510 [M−H]⁻; found: 166.0504.

### 2-ethyl-4-nitrophenol **2m′′**

The title compound was synthesized according to the general procedure A stated above 2-ethylphenol (122.17 mg, 1.00 mmol), 2-MeTHF (689 µL), NaNO₂ (207.00 mg, 3.00 mmol), and NaHSO₄ (360.18 mg, 3.00 mmol) were used, to afford **2m′′** as a yellow solid (73.55 mg, 0.44 mmol, 44%).

**^1^H NMR (600 MHz, CDCl₃):** *δ* 8.08 (d, *J =* 2.7 Hz, 1H), 8.01 (dd, *J =* 8.8, 2.7 Hz, 1H), 6.84 (d, *J =* 8.8 Hz, 1H), 5.96 (s, 1H), 2.70 (q, *J =* 7.6 Hz, 2H), 1.28 (t, *J =* 7.6 Hz, 3H).

**^13^C NMR (151 MHz, CDCl₃):** *δ* 159.3, 141.6, 131.1, 125.2, 123.5, 115.1, 22.7, 13.3.

The spectroscopic data closely match the ones previously reported in the literature.^[20]^

### 2-iodo-6-nitrophenol **2n′**

The title compound was synthesized according to the general procedure A stated above 2-iodophenol (220.01 mg, 1.00 mmol), 2-MeTHF (787 µL), NaNO₂ (207.00 mg, 3.00 mmol), and NaHSO₄ (360.18 mg, 3.00 mmol) were used, to afford **2n′** as a yellow solid (79.50 mg, 0.30 mmol, 30%).

**^1^H NMR (600 MHz, CDCl₃):** *δ* 11.35 (s, 1H), 8.13 (dd, *J =* 8.5, 1.6 Hz, 1H), 8.09 (dd, *J =* 7.8, 1.6 Hz, 1H), 6.80 (dd, *J =* 8.5, 7.8 Hz, 1H).

**^13^C NMR (151 MHz, CDCl₃):** *δ* 154.2, 147.3, 133.5, 125.6, 121.8, 87.1.

HRMS: calculated for C₆H₃INO₃⁻: 263.9163 [M−H]⁻; found: 263.9152.

### 2-iodo-4-nitrophenol **2n′′**

The title compound was synthesized according to the general procedure A stated above 2-iodophenol (220.01 mg, 1.00 mmol), 2-MeTHF (787 µL), NaNO₂ (207.00 mg, 3.00 mmol), and NaHSO₄ (360.18 mg, 3.00 mmol) were used, to afford **2n′′** as a yellow solid (76.85 mg, 0.29 mmol, 29%).

**^1^H NMR (600 MHz, CDCl₃):** *δ* 8.60 (d, *J =* 2.6 Hz, 1H), 8.17 (dd, *J =* 9.0, 2.6 Hz, 1H), 7.06 (d, *J =* 9.0 Hz, 1H), 6.10 (s, 1H).

**^13^C NMR (151 MHz, CDCl₃):** *δ* 160.4, 142.1, 134.4, 126.3, 114.8, 84.7.

The spectroscopic data closely match the ones previously reported in the literature.^[27]^

### 4-fluoro-2-nitrophenol **2o**

The title compound was synthesized according to the general procedure A stated above. 4-fluoro-phenol (112.10 mg, 1.00 mmol), 2-MeTHF (679 µL), NaNO₂ (207.00 mg, 3.00 mmol), and NaHSO₄ (360.18 mg, 3.00 mmol) were used, to afford **2o** as a yellow solid (128.82 mg, 0.82 mmol, 82%).

**^1^H NMR (600 MHz, CDCl₃):** *δ* 10.35 (s, 1H), 7.81 (dd, *J =* 9.1, 3.1 Hz, 1H), 7.36 (ddd, *J =* 9.4, 7.4, 3.1 Hz, 1H), 7.16 (dd, *J =* 9.3, 4.6 Hz, 1H).

**^13^C NMR (151 MHz, CDCl₃):** *δ* 155.0 (d, *J =* 243.8 Hz), 151.7 (d, *J =* 2.1 Hz), 133.0, 125.8 (d, *J =* 23.9 Hz), 121.4 (d, *J =* 7.3 Hz), 110.8 (d, *J =* 27.1 Hz).

**^19^F NMR (565 MHz, CDCl₃):** *δ* -120.58.

The spectroscopic data closely match the ones previously reported in the literature.^[28]^

### 4-chloro-2-nitrophenol **2p**

The title compound was synthesized according to the general procedure A stated above 4-chlorophenol (128.56 mg, 1.00 mmol), 2-MeTHF (696 µL), NaNO₂ (207.00 mg, 3.00 mmol), and NaHSO₄ (360.18 mg, 3.00 mmol) were used, to afford **2p** as a yellow solid (156.20 mg, 0.90 mmol, 90%).

**^1^H NMR (600 MHz, CDCl₃):** *δ* 8.08 (d, *J =* 2.6 Hz, 1H), 7.53 (dd, *J =* 9.0, 2.6 Hz, 1H), 7.12 (d, *J =* 9.0 Hz, 1H).

**^13^C NMR (151 MHz, CDCl₃):** *δ* 153.8, 137.7, 133.7, 125.3, 124.4, 121.6.

The spectroscopic data closely match the ones previously reported in the literature.^[28]^

### 5-fluoro-2-nitrophenol **2q′**

The title compound was synthesized according to the general procedure A stated above 3-fluorophenol (112.10 mg, 1.00 mmol), 2-MeTHF (679 µL), NaNO₂ (207.00 mg, 3.00 mmol), and NaHSO₄ (360.18 mg, 3.00 mmol) were used, to afford **2q′** as a yellow solid (59.70 mg, 0.38 mmol, 38%).

**^1^H NMR (600 MHz, CDCl₃):** *δ* 10.79 (s, 1H), 8.15 (dd, *J =* 9.4, 5.8 Hz, 1H), 6.83 (dd, *J =* 9.4, 2.7 Hz, 1H), 6.72 (ddd, *J =* 9.4, 7.3, 2.7 Hz, 1H).

**^13^C NMR (151 MHz, CDCl₃):** *δ* 167.9 (d, *J =* 260.2 Hz), 157.5 (d, *J =* 15.2 Hz), 130.8, 127.9 (d, *J =* 12.1 Hz), 109.0 (d, *J =* 24.3 Hz), 106.4 (d, *J =* 25.8 Hz).

**^19^F NMR (565 MHz, CDCl₃):** *δ* -96.32.

The spectroscopic data closely match the ones previously reported in the literature.^[29]^

### 3-fluoro-4-nitrophenol **2q′′**

The title compound was synthesized according to the general procedure A stated above 3-fluorophenol (112.10 mg, 1.00 mmol), 2-MeTHF (679 µL), NaNO₂ (207.00 mg, 3.00 mmol), and NaHSO₄ (360.18 mg, 3.00 mmol) were used, to afford **2q′′** as a yellow solid (29.85 mg, 0.19 mmol, 19%).

**^1^H NMR (600 MHz, CDCl₃):** *δ* 8.07 (dd, *J =* 8.6, 4.7 Hz, 1H), 6.79 – 6.69 (m, 2H), 6.18 (s, 1H).

**^13^C NMR (151 MHz, CDCl₃):** *δ* 162.3 (d, *J =* 11.9 Hz), 157.7 (d, *J =* 265.9 Hz), 131.0, 128.5 (d, *J =* 1.9 Hz), 111.9 (d, *J =* 3.1 Hz), 105.3 (d, *J =* 23.8 Hz).

**^19^F NMR (565 MHz, CDCl₃)**: *δ* -112.56.

HRMS: calculated for C₆H₃FNO₃⁻: 156.0097 [M−H]⁻; found: 156.0092.

### 3-fluoro-2-nitrophenol **2q′′′**

The title compound was synthesized according to the general procedure A stated above 3-fluorophenol (112.10 mg, 1.00 mmol), 2-MeTHF (679 µL), NaNO₂ (207.00 mg, 3.00 mmol), and NaHSO₄ (360.18 mg, 3.00 mmol) were used, to afford **2q′′′** as a yellow solid (12.57 mg, 0.08 mmol, 8%).

**^1^H NMR (600 MHz, CDCl₃):** *δ* 10.42 (s, 1H), 7.48 (td, *J =* 8.5, 5.5 Hz, 1H), 6.96 (d, *J =* 8.5 Hz, 1H), 6.81 – 6.72 (m, 1H).

**^13^C NMR (151 MHz, CDCl₃):** *δ* 157.0 (d, *J =* 267.0 Hz), 156.0, 136.4 (d, *J =* 11.7 Hz), 125.7, 115.2 (d, *J =* 3.9 Hz), 108.6 (d, *J =* 21.5 Hz).

**^19^F NMR (565 MHz, CDCl₃):** *δ* -115.17.

HRMS: calculated for C₆H₃FNO₃⁻: 156.0097 [M−H]⁻; found: 156.0091.

### 4-methoxy-2-nitrophenol **2r**

The title compound was synthesized according to the general procedure A stated above 4-methoxyphenol (124.14 mg, 1.00 mmol), 2-MeTHF (691 µL), NaNO₂ (207.00 mg, 3.00 mmol), and NaHSO₄ (360.18 mg, 3.00 mmol) were used, to afford **2r** as a yellow solid (143.77 mg, 0.85 mmol, 85%).

**^1^H NMR (600 MHz, CDCl₃):** *δ* 10.33 (s, 1H), 7.50 (d, *J =* 3.1 Hz, 1H), 7.22 (dd, *J =* 9.2, 3.1 Hz, 1H), 7.09 (d, *J =* 9.2 Hz, 1H), 3.82 (s, 3H).

**^13^C NMR (151 MHz, CDCl₃):** *δ* 152.8, 150.2, 133.2, 127.5, 121.4, 105.9, 56.2.

The spectroscopic data closely match the ones previously reported in the literature.^[30]^

### 2-methoxy-6-nitrophenol **2s′**

The title compound was synthesized according to the general procedure A stated above 2-methoxyphenol (124.14 mg, 1.00 mmol), 2-MeTHF (691 µL), NaNO₂ (207.00 mg, 3.00 mmol), and NaHSO₄ (360.18 mg, 3.00 mmol) were used, to afford ***2s′*** as a yellow solid (79.50 mg, 0.47 mmol, 47%).

**^1^H NMR (600 MHz, CDCl₃):** *δ* 10.75 (s, 1H), 7.69 (dd, *J =* 8.8, 1.4 Hz, 1H), 7.16 – 7.09 (m, 1H), 6.91 (m, 1H), 3.95 (s, 3H).

**^13^C NMR (151 MHz, CDCl₃):** *δ* 150.0, 146.3, 134.1, 118.8, 117.7, 116.1, 56.7.

The spectroscopic data closely match the ones previously reported in the literature.^[31]^

### 2-methoxy-4-nitrophenol **2s′′**

The title compound was synthesized according to the general procedure A stated above 2-methoxyphenol (124.14 mg, 1.00 mmol), 2-MeTHF (691 µL), NaNO₂ (207.00 mg, 3.00 mmol), and NaHSO₄ (360.18 mg, 3.00 mmol) were used, to afford **2s′′** as a yellow solid (52.43 mg, 0.31 mmol, 31%).

**^1^H NMR (600 MHz, CDCl₃):** *δ* 7.95 – 7.84 (m, 1H), 7.77 (s, 1H), 6.98 (d, *J =* 8.8 Hz, 1H), 6.22 (s, 1H), 4.00 (s, 3H).

**^13^C NMR (151 MHz, CDCl₃):** *δ* 151.8, 146.3, 141.3, 118.8, 114.1, 106.5, 56.6.

The spectroscopic data closely match the ones previously reported in the literature.^[32]^

### 4-(methylthio)-2-nitrophenol **2t**

The title compound was synthesized according to the general procedure A stated above 4-methylthiophenol (140.20 mg, 1.00 mmol), 2-MeTHF (707 µL), NaNO₂ (207.00 mg, 3.00 mmol), and NaHSO₄ (360.18 mg, 3.00 mmol) were used, to afford **2t** as a yellow solid (83.34 mg, 0.45 mmol, 45%).

**^1^H NMR (600 MHz, CDCl₃):** *δ* 10.48 (s, 1H), 7.96 (d, *J =* 2.4 Hz, 1H), 7.51 (dd, *J =* 8.8, 2.4 Hz, 1H), 7.11 (d, *J =* 8.8 Hz, 1H), 2.50 (s, 3H).

**^13^C NMR (151 MHz, CDCl₃):** *δ* 153.5, 137.6, 134.0, 130.6, 122.8, 120.8, 17.1.

The spectroscopic data closely match the ones previously reported in the literature.^[33]^

### 4-chloro-2-methoxy-6-nitrophenol **2u**

The title compound was synthesized according to the general procedure A stated above 4-chloro-2-methoxyphenol (158.58 mg, 1.00 mmol), 2-MeTHF (726 µL), NaNO₂ (207.00 mg, 3.00 mmol), and NaHSO₄ (360.18 mg, 3.00 mmol) were used, to afford **2u** as a yellow solid (156.76 mg, 0.77 mmol, 77%).

**^1^H NMR (600 MHz, CDCl₃):** *δ* 10.65 (s, 1H), 7.68 (d, *J =* 2.4 Hz, 1H), 7.07 (d, *J =* 2.4 Hz, 1H), 3.94 (s, 3H).

**^13^C NMR (151 MHz, CDCl₃):** *δ* 150.8, 145.5, 133.8, 124.6, 118.3, 115.2, 57.1.

The spectroscopic data closely match the ones previously reported in the literature.^[34]^

### 4-(benzyloxy)-2-nitrophenol **2v**

The title compound was synthesized according to the general procedure A stated above 4-benzyloxyphenol (200.24 mg, 1.00 mmol), 2-MeTHF (768 µL), NaNO₂ (207.00 mg, 3.00 mmol), and NaHSO₄ (360.18 mg, 3.00 mmol) were used, to afford **2v** as a yellow solid (176.57 mg, 0.72 mmol, 72%).

**^1^H NMR (600 MHz, CDCl₃):** *δ* 10.24 (s, 1H), 7.36 – 7.30 (m, 4H), 7.29 – 7.24 (m, 1H), 7.20 (d, *J =* 9.2 Hz, 1H), 7.00 (d, *J =* 9.2 Hz, 1H), 4.97 (s, 2H).

**^13^C NMR (151 MHz, CDCl₃):** *δ* 151.8, 150.3, 136.0, 133.1, 128.9, 128.5, 127.9, 127.7, 121.0, 107.4, 71.1.

The spectroscopic data closely match the ones previously reported in the literature.^[35]^

### 2-methoxy-4-methyl-6-nitrophenol **2w**

The title compound was synthesized according to the general procedure A stated above 2-methoxy-4-methylphenol (138.17 mg, 1.00 mmol), 2-MeTHF (705 µL), NaNO₂ (207.00 mg, 3.00 mmol), and NaHSO₄ (360.18 mg, 3.00 mmol) were used, to afford **2w** as a yellow solid (153.85 mg, 0.84 mmol, 84%).

**^1^H NMR (600 MHz, CDCl₃):** *δ* 10.57 (s, 1H), 7.44 (d, *J =* 2.0 Hz, 1H), 6.93 (d, *J =* 2.0 Hz, 1H), 3.90 (s, 3H), 2.31 (s, 3H).

**^13^C NMR (151 MHz, CDCl₃):** *δ* 149.7, 144.4, 133.7, 129.0, 119.4, 115.4, 56.7, 21.0.

The spectroscopic data closely match the ones previously reported in the literature.^[36]^

### 1-nitronaphthalen-2-ol **2x**

The title compound was synthesized according to the general procedure A stated above naphthalen-2-ol (144.17 mg, 1.00 mmol), 2-MeTHF (711 µL), NaNO₂ (207.00 mg, 3.00 mmol), and NaHSO₄ (360.18 mg, 3.00 mmol) were used, to afford **2x** as a yellow solid (122.96 mg, 0.65 mmol, 65%).

**^1^H NMR (600 MHz, CDCl₃):** *δ* 12.18 (s, 1H), 8.93 (dd, *J =* 8.8, 1.0 Hz, 1H), 8.01 (d, *J =* 9.0 Hz, 1H), 7.85 – 7.81 (m, 1H), 7.74 (ddd, *J =* 8.7, 7.0, 1.5 Hz, 1H), 7.51 (ddd, *J =* 8.0, 7.0, 1.1 Hz, 1H), 7.26 (d, *J =* 9.1 Hz, 1H).

**^13^C NMR (151 MHz, CDCl₃):** *δ* 159.0, 139.4, 131.1, 129.6, 129.5, 128.9, 127.1, 125.8, 123.4, 119.5.

The spectroscopic data closely match the ones previously reported in the literature.^[37]^

### 2-nitronaphthalen-1-ol **2y**

The title compound was synthesized according to the general procedure A stated above naphthalen-1-ol (144.17 mg, 1.00 mmol), 2-MeTHF (711 µL), NaNO₂ (207.00 mg, 3.00 mmol), and NaHSO₄ (360.18 mg, 3.00 mmol) were used, to afford **2y** as a yellow solid (104.04 mg, 0.55 mmol, 55%).

**^1^H NMR (600 MHz, CDCl₃):** *δ* 12.24 (s, 1H), 8.52 (d, *J =* 8.4 Hz, 1H), 8.02 (d, *J =* 9.3 Hz, 1H), 7.82 (d, *J =* 8.4 Hz, 1H), 7.73 (t, *J =* 7.8 Hz, 1H), 7.63 (t, *J =* 7.8 Hz, 1H), 7.35 (d, *J =* 9.3 Hz, 1H).

**^13^C NMR (151 MHz, CDCl₃):** *δ* 155.8, 137.6, 131.5, 128.0, 127.2, 125.2, 120.3, 119.5.

The spectroscopic data closely match the ones previously reported in the literature.^[38]^

### 7-bromo-1-nitronaphthalen-2-ol **2z**

The title compound was synthesized according to the general procedure A stated above. 7-bromonaphthalen-2-ol (223.07 mg, 1.00 mmol), 2-MeTHF (790 µL), NaNO₂ (207.00 mg, 3.00 mmol), and NaHSO₄ (360.18 mg, 3.00 mmol) were used, to afford **2z** as a pale-yellow solid (115.27 mg, 0.43 mmol, 43%).

**^1^H NMR (600 MHz, CDCl₃):** *δ* 12.23 (s, 1H), 9.15 (d, *J =* 1.8 Hz, 1H), 7.96 (d, *J =* 9.1 Hz, 1H), 7.67 (d, *J =* 8.5 Hz, 1H), 7.61 (dd, *J =* 8.5, 1.8 Hz, 1H), 7.27 (d, *J =* 9.1 Hz, 1H).

**^13^C NMR (151 MHz, CDCl₃):** *δ* 159.5, 139.0, 130.7, 129.3, 128.0, 127.4, 127.2, 126.5, 126.0, 120.0.

**HRMS:** calculated for C₁₀H₅BrNO₃⁻: 265.9453 [M−H]⁻; found: 265.9443.

### (((9H-fluoren-9-yl)methoxy)carbonyl)-3-nitro-L-tyrosine **2aa**

The title compound was synthesized according to the general procedure A stated above. (((9H-fluoren-9-yl)methoxy)carbonyl)tyrosine (403.43 mg, 1.00 mmol), 2-MeTHF (970 µL), NaNO₂ (207.00 mg, 3.00 mmol), and NaHSO₄ (360.18 mg, 3.00 mmol) were used, to afford **2aa** as a yellow solid (313.90 mg, 0.70 mmol, 70%).

**^1^H NMR (600 MHz, DMSO-d₆):** *δ* 10.79 (s, 1H), 7.87 (d, *J =* 7.6 Hz, 2H), 7.83 (d, *J =* 2.2 Hz, 1H), 7.75 (d, *J =* 8.6 Hz, 1H), 7.61 (dd, *J =* 7.5, 5.3 Hz, 2H), 7.46 (dd, *J =* 8.6, 2.2 Hz, 1H), 7.40 (t, *J =* 7.5 Hz, 2H), 7.28 (m, 2H), 7.04 (d, *J =* 8.6 Hz, 1H), 4.22 – 4.14 (m, 4H), 3.07 (dd, *J =* 13.9, 4.4 Hz, 1H), 2.84 (dd, *J =* 13.9, 10.7 Hz, 1H).

**^13^C NMR (151 MHz, DMSO-d₆):** *δ* 173.0, 155.9, 150.8, 143.7, 143.6, 140.6, 140.6, 136.2, 136.1, 129.1, 127.6, 127.0, 125.4, 125.2, 125.1, 120.1, 118.9, 65.6, 55.2, 46.5, 35.0.

The spectroscopic data closely match the ones previously reported in the literature.^[39]^

### *(*8R,9S,13S,14S)-13-methyl-2-nitro-7,8,9,11,12,13,14,15,16,17-decahydro-6H-cyclopenta[a]phenanthrene-3,17-diol **2ab**

The title compound was synthesized according to the general procedure A stated above. β-Estradiol (272.39 mg, 1.00 mmol), 2-MeTHF (839 µL), NaNO₂ (207.00 mg, 3.00 mmol), and NaHSO₄ (360.18 mg, 3.00 mmol) were used, to afford **2ab** as a yellow solid (199.95 mg, 0.63 mmol, 63%).

**^1^H NMR (600 MHz, CDCl₃):** *δ* 10.41 (s, 1H), 7.97 (s, 1H), 6.85 (s, 1H), 5.35 (t, *J =* 8.8 Hz, 1H), 2.97 – 2.87 (m, 2H), 2.35 – 2.21 (m, 3H), 1.96 – 1.75 (m, 4H), 1.54 – 1.41 (m, 6H), 0.79 (s, 3H).

**^13^C NMR (151 MHz, CDCl₃):** *δ* 152.8, 149.0, 133.4, 131.7, 121.5, 118.9, 81.6, 50.2, 43.4, 43.3, 37.9, 36.3, 29.7, 27.2, 26.5, 25.8, 23.2, 11.9.

The spectroscopic data closely match the ones previously reported in the literature.^[40]^

### (8R,9S,13S,14S)-3-hydroxy-13-methyl-2-nitro-6,7,8,9,11,12,13,14,15,16-decahydro-17H-cyclopenta[a]phenanthren-17-one **2ac**

The title compound was synthesized according to the general procedure A stated above. Estrone (270.38 mg, 1.00 mmol), 2-MeTHF (837 µL), NaNO₂ (207.00 mg, 3.00 mmol), and NaHSO₄ (360.18 mg, 3.00 mmol) were used, to afford **2ac** as a yellow solid (211.30 mg, 0.67 mmol, 67%).

**^1^H NMR (600 MHz, CDCl₃)**: *δ* 10.41 (s, 1H), 7.98 (s, 1H), 6.87 (s, 1H), 3.02 – 2.87 (m, 2H), 2.52 (ddd, *J =* 19.1, 8.9, 1.1 Hz, 1H), 2.47 – 2.40 (m, 1H), 2.26 – 2.20 (m, 1H), 2.19 – 2.12 (m, 1H), 2.11 – 1.98 (m, 3H), 1.73 – 1.33 (m, 6H), 0.92 (s, 3H).

**^13^C NMR (151 MHz, CDCl₃)**: *δ* 220.3, 153.0, 148.9, 133.2, 131.9, 121.7, 119.1, 50.5, 47.9, 43.6, 37.9, 35.9, 31.4, 29.8, 26.0, 25.8, 21.7, 13.9.

The spectroscopic data closely match the ones previously reported in the literature.^[41]^

## Products 4a, 4e–4u

### 2,4-dibromo-6-nitrophenol **4a′**

The title compound was synthesized according to the general procedure B stated above. 2,4,6-tribromophenol (330.80 mg, 1.00 mmol), 2-MeTHF (898 µL), NaNO₂ (207.00 mg, 3.00 mmol), and NaHSO₄ (360.18 mg, 3.00 mmol) were used, to afford **4a′** as a yellow solid (139.54 mg, 0.47 mmol, 47%).

**^1^H NMR (600 MHz, CDCl₃):** *δ* 11.04 (s, 1H), 8.24 (d, *J =* 2.4 Hz, 1H), 7.99 (d, *J =* 2.4 Hz, 1H).

**^13^C NMR (151 MHz, CDCl₃):** *δ* 151.6, 143.1, 134.6, 126.9, 114.7, 111.7.

The spectroscopic data closely match the ones previously reported in the literature.^[42]^

### 2,6-dibromo-4-nitrophenol **4a′′**

The title compound was synthesized according to the general procedure B stated above. 2,4,6-tribromophenol (330.80 mg, 1.00 mmol), 2-MeTHF (898 µL), NaNO₂ (207.00 mg, 3.00 mmol), and NaHSO₄ (360.18 mg, 3.00 mmol) were used, to afford **4a′′** as a yellow solid (136.57 mg, 0.46 mmol, 46%).

**^1^H NMR (600 MHz, CDCl₃):** *δ* 8.41 (s, 2H), 6.53 (s, 1H).

**^13^C NMR (151 MHz, CDCl₃):** *δ* 155.1, 141.7, 128.0, 109.8.

The spectroscopic data closely match the ones previously reported in the literature.^[43]^

### 4-bromo-2,3-dimethyl-6-nitrophenol **4e′**

The title compound was synthesized according to the general procedure B stated above. 4,6-dibromo-2,3-dimethylphenol (279.96 mg, 1.00 mmol), 2-MeTHF (847 µL), NaNO₂ (207.00 mg, 3.00 mmol), and NaHSO₄ (360.18 mg, 3.00 mmol) were used, to afford **4e′** as a yellow solid (86.12 mg, 0.35 mmol, 35%).

**^1^H NMR (600 MHz, CDCl₃):** *δ* 10.91 (s, 1H), 8.18 (s, 1H), 2.44 (s, 3H), 2.31 (s, 3H).

**^13^C NMR (151 MHz, CDCl₃):** *δ* 152.3, 146.9, 131.8, 129.0, 124.9, 115.0, 20.8, 13.0.

**HRMS:** calculated for C₈H₇BrNO₃⁻: 243.9609 [M−H]⁻; found: 243.9599.

### 6-bromo-2,3-dimethyl-4-nitrophenol **4e′′**

The title compound was synthesized according to the general procedure B stated above. 4,6-dibromo-2,3-dimethylphenol (279.96 mg, 1.00 mmol), 2-MeTHF (847 µL), NaNO₂ (207.00 mg, 3.00 mmol), and NaHSO₄ (360.18 mg, 3.00 mmol) were used, to afford **4e′′** as a yellow solid (86.12 mg, 0.35 mmol, 35%).

**^1^H NMR (600 MHz, CDCl₃):** *δ* 11.31 (s, 1H), 8.61 (s, 1H), 2.54 (s, 3H), 2.37 (s, 3H).

**^13^C NMR (151 MHz, CDCl₃):** δ 155.4, 141.5, 130.7, 130.5, 125.2, 119.6, 17.1, 12.6.

**HRMS**: calculated for C₈H₇BrNO₃⁻: 243.9609 [M−H]⁻; found: 243.9594.

### 2-bromo-4-methyl-6-nitrophenol **4f**

The title compound was synthesized according to the general procedure B stated above. 2,6-dibromo-4-methylphenol (265.93 mg, 1.00 mmol), 2-MeTHF (833 µL), NaNO₂ (207.00 mg, 3.00 mmol), and NaHSO₄ (360.18 mg, 3.00 mmol) were used, to afford **4f** as a yellow solid (197.23 mg, 0.85 mmol, 85%).

**^1^H NMR (600 MHz, CDCl₃):** *δ* 10.96 (s, 1H), 7.89 (d, *J =* 2.2 Hz, 1H), 7.70 (d, *J =* 2.2 Hz, 1H), 2.35 (s, 3H).

**^13^C NMR (151 MHz, CDCl₃):** *δ* 150.2, 141.9, 134.0, 130.7, 124.2, 112.9, 20.2.

The spectroscopic data closely match the ones previously reported in the literature.^[42]^

### 4-methyl-2,6-dinitrophenol **4g**

The title compound was synthesized according to the general procedure B stated above. 2,6-dibromo-4-methylphenol (265.93 mg, 1.00 mmol), 2-MeTHF (833 µL), NaNO₂ (207.00 mg, 3.00 mmol), and NaHSO₄ (360.18 mg, 3.00 mmol) were used, to afford **4g** as a yellow solid (19.81 mg, 0.10 mmol, 10%).

**^1^H NMR (600 MHz, CDCl₃):** *δ* 11.27 (s, 1H), 8.14 (s, 2H), 2.45 (s, 3H).

**^13^C NMR (151 MHz, CDCl₃):** *δ* 147.5, 137.2, 131.8, 129.5, 20.4.

The spectroscopic data closely match the ones previously reported in the literature.^[41]^

### 4,6-dibromo-3-methyl-2-nitrophenol **4h′**

The title compound was synthesized according to the general procedure B stated above. 2,4,6-tribromo-3-methylphenol (344.83 mg, 1.00 mmol), 2-MeTHF (912 µL), NaNO₂ (207.00 mg, 3.00 mmol), and NaHSO₄ (360.18 mg, 3.00 mmol) were used, to afford **4h′** as a yellow solid (217.65 mg, 0.70 mmol, 70%).

**^1^H NMR (600 MHz, CDCl₃):** *δ* 11.18 (s, 1H), 8.33 (s, 1H), 2.68 (s, 3H).

**^13^C NMR (151 MHz, CDCl₃):** *δ* 151.5, 148.7, 132.4, 126.8, 116.4, 114.5, 25.3.

HRMS: calculated for C₇H₄Br₂NO₃⁻: 307.8558 [M−H]⁻; found: 307.8550.

### 2,6-dibromo-3-methyl-4-nitrophenol **4h′′**

The title compound was synthesized according to the general procedure B stated above. 2,4,6-tribromo-3-methylphenol (344.83 mg, 1.00 mmol), 2-MeTHF (912 µL), NaNO₂ (207.00 mg, 3.00 mmol), and NaHSO₄ (360.18 mg, 3.00 mmol) were used, to afford **4h′′** as a yellow solid (62.19 mg, 0.20 mmol, 20%).

**^1^H NMR (600 MHz, CDCl₃)**: *δ* 8.14 (s, 1H), 6.47 (s, 1H), 2.64 (s, 3H).

**^13^C NMR (151 MHz, CDCl₃):** *δ* 153.4, 144.1, 134.7, 128.5, 114.9, 106.5, 20.6.

HRMS: calculated for C₇H₄Br₂NO₃⁻: 307.8558 [M−H]⁻; found: 307.8546.

### 4-bromo-2-methyl-6-nitrophenol **4i′**

The title compound was synthesized according to the general procedure B stated above. 4,6-dibromo-2-methylphenol (265.93 mg, 1.00 mmol), 2-MeTHF (833 µL), NaNO₂ (207.00 mg, 3.00 mmol), and NaHSO₄ (360.18 mg, 3.00 mmol) were used, to afford **4i′** as a yellow solid (118.34 mg, 0.51 mmol, 51%).

**^1^H NMR (600 MHz, CDCl₃):** *δ* 10.81 (s, 1H), 8.09 (d, *J =* 2.4 Hz, 1H), 7.58 – 7.52 (m, 1H), 2.32 (s, 3H).

**^13^C NMR (151 MHz, CDCl₃):** *δ* 152.9, 140.7, 133.9, 131.9, 124.9, 111.1, 15.9.

The spectroscopic data closely match the ones previously reported in the literature.^[44]^

### 2-bromo-6-methyl-4-nitrophenol **4i′′**

The title compound was synthesized according to the general procedure B stated above. 2,4-dibromo-6-methylphenol (265.93 mg, 1.00 mmol), 2-MeTHF (833 µL), NaNO₂ (207.00 mg, 3.00 mmol), and NaHSO₄ (360.18 mg, 3.00 mmol) were used, to afford **4i′′** as a yellow solid (69.61 mg, 0.30 mmol, 30%).

**^1^H NMR (600 MHz, CDCl₃):** *δ* 8.27 (d, *J =* 2.6 Hz, 1H), 8.02 (d, *J =* 2.6 Hz, 1H), 6.20 (s, 1H), 2.38 (s, 3H).

**^13^C NMR (151 MHz, CDCl₃):** *δ* 155.9, 141.1, 126.5, 125.9, 125.6, 109.6, 16.8.

The spectroscopic data closely match the ones previously reported in the literature.^[45]^

### 2-bromo-4-fluoro-6-nitrophenol **4j**

The title compound was synthesized according to the general procedure B stated above. 2,6-dibromo-4-fluorophenol (269.90 mg, 1.00 mmol), 2-MeTHF (837 µL), NaNO₂ (207.00 mg, 3.00 mmol), and NaHSO₄ (360.18 mg, 3.00 mmol) were used, to afford **4j** as a yellow solid (212.40 mg, 0.90 mmol, 90%).

**^1^H NMR (600 MHz, CDCl₃):** *δ* 10.89 (s, 1H), 7.84 (dd, *J =* 8.0, 3.1 Hz, 1H), 7.68 (dd, *J =* 7.1, 3.1 Hz, 1H).

**^13^C NMR (151 MHz, CDCl₃):** *δ* 154.2 (d, *J =* 247.1 Hz), 149.2 (d, *J =* 2.4 Hz), 133.4 (d, *J =* 9.1 Hz), 129.1 (d, *J =* 26.0 Hz), 114.1 (d, *J =* 9.3 Hz), 110.6 (d, *J =* 27.0 Hz).

**^19^F NMR (565 MHz, CDCl₃):** *δ* -119.41.

HRMS: calculated for C₆H₂BrFNO₃⁻: 233.9208 [M−H]⁻; found: 233.9198.

### 4-bromo-2-fluoro-6-nitrophenol **4k′**

The title compound was synthesized according to the general procedure B stated above. 4,6-dibromo-2-fluorophenol (269.90 mg, 1.00 mmol), 2-MeTHF (837 µL), NaNO₂ (207.00 mg, 3.00 mmol), and NaHSO₄ (360.18 mg, 3.00 mmol) were used, to afford **4k′** as a yellow solid (84.96 mg, 0.36 mmol, 36%).

**^1^H NMR (600 MHz, CDCl₃):** *δ* 10.38 (s, 1H), 8.08 (t, *J =* 2.2 Hz, 1H), 7.57 (dd, *J =* 9.2, 2.2 Hz, 1H).

**^13^C NMR (151 MHz, CDCl₃):** *δ* 152.4 (d, *J =* 256.4 Hz), 144.3 (d, *J =* 15.0 Hz), 135.1, 126.6 (d, *J =* 20.5 Hz), 122.8 (d, *J =* 4.0 Hz), 110.1 (d, *J =* 8.5 Hz).

**^19^F NMR (565 MHz, CDCl₃):** *δ* -128.3.

The spectroscopic data closely match the ones previously reported in the literature.^[46]^

### 2-bromo-6-fluoro-4-nitrophenol **4k′′**

The title compound was synthesized according to the general procedure B stated above. 2,4-dibromo-6-fluorophenol (269.90 mg, 1.00 mmol), 2-MeTHF (837 µL), NaNO₂ (207.00 mg, 3.00 mmol), and NaHSO₄ (360.18 mg, 3.00 mmol) were used, to afford **4k′′** as a yellow solid (125.08 mg, 0.53 mmol, 53%).

**^1^H NMR (600 MHz, CDCl₃):** *δ* 8.26 (dd, *J =* 2.8, 1.4 Hz, 1H), 8.00 – 7.94 (m, 1H).

**^13^C NMR (151 MHz, CDCl₃):** *δ* 150.1 (d, *J =* 248.7 Hz), 148.8 (d, *J =* 15.5 Hz), 140.2, 124.6 (d, *J =* 2.9 Hz), 111.7 (d, *J =* 23.7 Hz), 111.2 (d, *J =* 3.1 Hz).

**^19^F NMR (565 MHz, CDCl₃):** *δ* -129.3.

The spectroscopic data closely match the ones previously reported in the literature.^[47]^

### 4-bromo-2-chloro-6-nitrophenol **4l′**

The title compound was synthesized according to the general procedure B stated above. 4,6-dibromo-2-chlorophenol (286.35 mg, 1.00 mmol), 2-MeTHF (854 µL), NaNO₂ (207.00 mg, 3.00 mmol), and NaHSO₄ (360.18 mg, 3.00 mmol) were used, to afford **4l′** as a yellow solid (121.16 mg, 0.48 mmol, 48%).

**^1^H NMR (600 MHz, CDCl₃):** *δ* 10.93 (s, 1H), 8.20 (d, *J =* 2.4 Hz, 1H), 7.82 (d, *J =* 2.4 Hz, 1H).

**^13^C NMR (151 MHz, CDCl₃):** *δ* 150.8, 140.1, 134.8, 126.2, 126.1, 110.9.

The spectroscopic data closely match the ones previously reported in the literature.^[42]^

### 2-bromo-6-chloro-4-nitrophenol **4l′′**

The title compound was synthesized according to the general procedure B stated above. 2,4-dibromo-6-chlorophenol (286.35 mg, 1.00 mmol), 2-MeTHF (854 µL), NaNO₂ (207.00 mg, 3.00 mmol), and NaHSO₄ (360.18 mg, 3.00 mmol) were used, to afford **4l′′** as a yellow solid (118.64 mg, 0.47 mmol, 47%).

**^1^H NMR (600 MHz, CDCl₃):** *δ* 8.37 (d, *J =* 2.6 Hz, 1H), 8.27 (d, *J =* 2.6 Hz, 1H), 6.55 (s, 1H).

**^13^C NMR (151 MHz, CDCl₃):** *δ* 154.3, 141.3, 127.4, 125.0, 121.2, 110.2.

The spectroscopic data closely match the ones previously reported in the literature.^[48]^

### 2,4-dichloro-6-nitrophenol **4m**

The title compound was synthesized according to the general procedure B stated above. 2,4-dichloro-6-bromophenol (241.89 mg, 1.00 mmol), 2-MeTHF (809 µL), NaNO₂ (207.00 mg, 3.00 mmol), and NaHSO₄ (360.18 mg, 3.00 mmol) were used, to afford **4m** as a yellow solid (178.87 mg, 0.86 mmol, 86%).

**^1^H NMR (600 MHz, CDCl₃):** *δ* 10.91 (s, 1H), 8.06 (d, *J =* 2.6 Hz, 1H), 7.70 (d, *J =* 2.6 Hz, 1H).

**^13^C NMR (151 MHz, CDCl₃):** *δ* 150.3, 137.3, 134.2, 125.8, 124.7, 123.0.

The spectroscopic data closely match the ones previously reported in the literature.^[49]^

### 2,4-diiodo-6-nitrophenol **4n′**

The title compound was synthesized according to the general procedure B stated above. 2,4,6-triiodophenol (471.80 mg, 1.00 mmol), 2-MeTHF (1039 µL), NaNO₂ (207.00 mg, 3.00 mmol), and NaHSO₄ (360.18 mg, 3.00 mmol) were used, to afford **4n′** as a yellow solid (191.54 mg, 0.49 mmol, 49%).

**^1^H NMR (600 MHz, CDCl₃):** *δ* 11.26 (s, 1H), 8.40 (d, *J =* 2.0 Hz, 1H), 8.33 (d, *J =* 2.0 Hz, 1H).

**^13^C NMR (151 MHz, CDCl₃):** *δ* 154.2, 154.0, 133.8, 88.7, 81.3.

The spectroscopic data closely match the ones previously reported in the literature.^[50]^

### 2,6-diiodo-4-nitrophenol **4n′′**

The title compound was synthesized according to the general procedure B stated above. 2,4,6-triiodophenol (471.80 mg, 1.00 mmol), 2-MeTHF (1039 µL), NaNO₂ (207.00 mg, 3.00 mmol), and NaHSO₄ (360.18 mg, 3.00 mmol) were used, to afford **4n′′** as a yellow solid (191.54 mg, 0.49 mmol, 49%).

**^1^H NMR (600 MHz, CDCl₃):** *δ* 8.59 (s, 2H), 6.37 (s, 1H).

**^13^C NMR (151 MHz, CDCl₃):** *δ* 159.1, 142.3, 134.9, 80.9.

The spectroscopic data closely match the ones previously reported in the literature.^[50]^

### 2,6-di-*tert*-butyl-4-methyl-4-nitrocyclohexa-2,5-dien-1-one **4o**

The title compound was synthesized according to the general procedure B stated above. 2,6-di-*tert*-butyl-4-methylphenol (220.36 mg, 1.00 mmol), 2-MeTHF (788 µL), NaNO₂ (207.00 mg, 3.00 mmol), and NaHSO₄ (360.18 mg, 3.00 mmol) were used, to afford **4o** as a yellow solid (185.75 mg, 0.70 mmol, 70%).

**^1^H NMR (600 MHz, CDCl₃):** *δ* 6.56 (s, 2H), 1.42 (s, 3H), 1.22 (s, 18H).

**^13^C NMR (151 MHz, CDCl₃):** *δ* 186.2, 145.6, 143.3, 67.5, 34.6, 29.5, 28.1.

The spectroscopic data closely match the ones previously reported in the literature.^[51]^

### 2,6-di-*tert*-butyl-4-ethyl-4-nitrocyclohexa-2,5-dien-1-one **4p**

The title compound was synthesized according to the general procedure B stated above. 2,6-di-*tert*-butyl-4-ethylphenol (234.38 mg, 1.00 mmol), 2-MeTHF (802 µL), NaNO₂ (207.00 mg, 3.00 mmol), and NaHSO₄ (360.18 mg, 3.00 mmol) were used, to afford **4p** as a yellow solid (178.80 mg, 0.64 mmol, 64%).

**^1^H NMR (600 MHz, CDCl₃):** *δ* 6.44 (s, 2H), 1.75 (q, *J =* 7.5 Hz, 2H), 1.22 (s, 18H), 0.74 (t, *J =* 7.5 Hz, 3H).

**^13^C NMR (151 MHz, CDCl₃):** *δ* 186.5, 147.2, 142.1, 70.8, 34.8, 33.8, 29.6, 8.4.

HRMS: calculated for C₁₆H₂₆NO₃⁺: 280.1907 [M+H]^+^; found: 280.1912

### Methyl 3-(3,5-di-*tert*-butyl-1-nitro-4-oxocyclohexa-2,5-dien-1-yl)propanoate **4q**

The title compound was synthesized according to the general procedure B stated above. methyl 3-(3,5-di-*tert*-butyl-4-hydroxyphenyl)propanoate (292.42 mg, 1.00 mmol), 2-MeTHF (860 µL), NaNO₂ (207.00 mg, 3.00 mmol), and NaHSO₄ (360.18 mg, 3.00 mmol) were used, to afford **4q** as a yellow solid (111.35 mg, 0.33 mmol, 33%).

**^1^H NMR (600 MHz, CDCl₃):** *δ* 6.46 (s, 2H), 3.65 (s, 3H), 2.24 (m, 2H), 2.06 – 2.02 (m, 2H), 1.20 (s, 18H).

**^13^C NMR (151 MHz, CDCl₃):** *δ* 186.1, 173.6, 147.3, 141.4, 69.4, 51.9, 35.6, 34.8, 29.5, 29.0.

HRMS: calculated for C₁₈H₂₆NO₅⁻: 336.1816 [M-H]^-^; found: 336.1802.

### 2,6-dichlorocyclohexa-2,5-diene-1,4-dione **4r**

The title compound was synthesized according to the general procedure B stated above. 2,4,6-trichlorophenol (197.45 mg, 1.00 mmol), 2-MeTHF (765 µL), NaNO₂ (207.00 mg, 3.00 mmol), and NaHSO₄ (360.18 mg, 3.00 mmol) were used, to afford **4r** as a yellow solid (127.43 mg, 0.72 mmol, 72%).

**^1^H NMR (600 MHz, CDCl₃):** *δ* 7.03 (s, 2H).

**^13^C NMR (151 MHz, CDCl₃):** *δ* 182.6, 172.8, 143.7, 133.9.

The spectroscopic data closely match the ones previously reported in the literature.^[52]^

### 3,3′,5,5′-tetramethyl-[1,1′-bi(cyclohexylidene)]-2,2′,5,5′-tetraene-4,4′-dione **4s**

The title compound was synthesized according to the general procedure B stated above. 3,3′,5,5′-tetramethyl-[1,1′-biphenyl]-4,4′-diol (242.32 mg, 1.00 mmol), 2-MeTHF (809 µL), NaNO₂ (207.00 mg, 3.00 mmol), and NaHSO₄ (360.18 mg, 3.00 mmol) were used, to afford **4s** as a yellow solid (132.17 mg, 0.55 mmol, 55%).

**^1^H NMR (600 MHz, CDCl₃):** *δ* 7.72 (s, 4H), 2.16 (s, 12H).

**^13^C NMR (151 MHz, CDCl₃):** *δ* 187.4, 139.1, 135.8, 129.8, 17.3.

The spectroscopic data closely match the ones previously reported in the literature.^[53]^

### 3,3′,5,5′-tetra-*tert*-butyl-[1,1′-bi(cyclohexylidene)]-2,2′,5,5′-tetraene-4,4′-dione **4t**

The title compound was synthesized according to the general procedure B stated above. 4-bromo-2,6-di-*tert*-butylphenol (285.22 mg, 1.00 mmol), 2-MeTHF (853 µL), NaNO₂ (207.00 mg, 3.00 mmol), and NaHSO₄ (360.18 mg, 3.00 mmol) were used, to afford **4t** as a red solid (20.43 mg, 0.05 mmol, 10% based on the theoretical 0.50 mmol maximum dimer yield from 1.00 mmol substrate ).

**^1^H NMR (600 MHz, CDCl₃):** *δ* 7.71 (s, 4H), 1.36 (s, 36H).

**^13^C NMR (151 MHz, CDCl₃):** *δ* 186.6, 150.6, 136.3, 126.1, 36.1, 29.7.

The spectroscopic data closely match the ones previously reported in the literature.^[54]^

### 2,6-di-*tert*-butylcyclohexa-2,5-diene-1,4-dione **4u**

The title compound was synthesized according to the general procedure B stated above. 2,4,6-tri-*tert*-butylphenol (262.43 mg, 1.00 mmol), 2-MeTHF (829 µL), NaNO₂ (207.00 mg, 3.00 mmol), and NaHSO₄ (360.18 mg, 3.00 mmol) were used, to afford **4u** as a yellow solid (136.59 mg, 0.62 mmol, 62%).

**^1^H NMR (600 MHz, CDCl₃):** *δ* 6.50 (s, 2H), 1.29 (s, 18H).

**^13^C NMR (151 MHz, CDCl₃):** *δ* 189.2, 187.9, 158.0, 130.2, 35.7, 29.5.

The spectroscopic data closely match the ones previously reported in the literature.^[55]^

## NMR spectra

**¹H NMR (600 MHz, CDCl₃)**

**Figure S6**. ¹H NMR spectrum of 2-nitrophenol **(2a′)**

**¹³C NMR (151 MHz, CDCl₃)**

**Figure S7**. ¹³C NMR spectrum of 2-nitrophenol **(2a′)**

**¹H NMR (600 MHz, CDCl₃)**

**Figure S8**. ¹H NMR spectrum of 4-nitrophenol **(2a′′)**

**¹³C NMR (151 MHz, CDCl₃)**

**Figure S9**. ¹³C NMR spectrum of 4-nitrophenol **(2a′′)**

**¹H NMR (600 MHz, CDCl₃)**

**Figure S10**. ¹H NMR spectrum of 2,4-dimethyl-6-nitrophenol **2b**

**¹³C NMR (151 MHz, CDCl₃)**

**Figure S11**. ¹³C NMR spectrum of 2,4-dimethyl-6-nitrophenol **2b**

**¹H NMR (600 MHz, CDCl₃)**

**Figure S12**. ¹H NMR spectrum of 2-(tert-butyl)-4-methyl-6-nitrophenol **2c**

**¹³C NMR (151 MHz, CDCl₃)**

**Figure S13**. ¹³C NMR spectrum of 2-(tert-butyl)-4-methyl-6-nitrophenol **2c**

**¹H NMR (600 MHz, CDCl₃)**

**Figure S14**. ¹H NMR spectrum of 2,6-di-tert-butyl-4-nitrophenol **2d**

**¹³C NMR (151 MHz, CDCl₃)**

**Figure S15**. ¹³C NMR spectrum of 2,6-di-tert-butyl-4-nitrophenol **2d**

**¹H NMR (600 MHz, CDCl₃)**

**Figure S16**. ¹H NMR spectrum of 4-ethyl-2-nitrophenol **2e**

**¹³C NMR (151 MHz, CDCl₃)**

**Figure S17**. ¹³C NMR spectrum of 4-ethyl-2-nitrophenol **2e**

**¹H NMR (600 MHz, CDCl₃)**

**Figure S18**. ¹H NMR spectrum of 2-nitro-4-(tert-pentyl) phenol **2f**

**¹³C NMR (151 MHz, CDCl₃)**

**Figure S19**. ¹³C NMR spectrum of 2-nitro-4-(tert-pentyl) phenol **2f**

**¹H NMR (600 MHz, CDCl₃)**

**Figure S20**. ¹H NMR spectrum of 4-(tert-butyl)-2-nitrophenol **2g**

**¹³C NMR (151 MHz, CDCl₃)**

**Figure S21**. ¹³C NMR spectrum of 4-(tert-butyl)-2-nitrophenol **2g**

**¹H NMR (600 MHz, CDCl₃)**

**Figure S22**. ¹H NMR spectrum of 4-(isopropyl)-2-nitrophenol **2h**

**¹³C NMR (151 MHz, CDCl₃)**

**Figure S23**. ¹³C NMR spectrum of 4-(isopropyl)-2-nitrophenol **2h**

**¹H NMR (600 MHz, CDCl₃)**

**Figure S24**. ¹H NMR spectrum of 4-methyl-2-nitrophenol **2i**

**¹³C NMR (151 MHz, CDCl₃)**

**Figure S25**. ¹³C NMR spectrum of 4-methyl-2-nitrophenol **2i**

**¹H NMR (600 MHz, CDCl₃)**

**Figure S26**. ¹H NMR spectrum of 2-allyl-6-nitrophenol **2j′**

**¹³C NMR (151 MHz, CDCl₃)**

**Figure S27**. ¹³C NMR spectrum of 2-allyl-6-nitrophenol **2j′**

**¹H NMR (600 MHz, CDCl₃)**

**Figure S28**. ¹H NMR spectrum of 2-allyl-4-nitrophenol **2j′′**

**¹³C NMR (151 MHz, CDCl₃)**

**Figure S29**. ¹³C NMR spectrum of 2-allyl-4-nitrophenol **2j′′**

**¹H NMR (600 MHz, CDCl₃)**

**Figure S30**. ¹H NMR spectrum of 2-methyl-6-nitrophenol **2k′**

**¹³C NMR (151 MHz, CDCl₃)**

**Figure S31**. ¹³C NMR spectrum of 2-methyl-6-nitrophenol **2k′**

**¹H NMR (600 MHz, CDCl₃)**

**Figure S32**. ¹H NMR spectrum of 2-methyl-4-nitrophenol **2k′′**

**¹³C NMR (151 MHz, CDCl₃)**

**Figure S33**. ¹³C NMR spectrum of 2-methyl-4-nitrophenol **2k′′**

**¹H NMR (600 MHz, CDCl₃)**

**Figure S34**. ¹H NMR spectrum of 2-isopropyl-6-nitrophenol **2l′**

**¹³C NMR (151 MHz, CDCl₃)**

**Figure S35**. ¹³C NMR spectrum of 2-isopropyl-6-nitrophenol **2l′**

**¹H NMR (600 MHz, CDCl₃)**

**Figure S36**. ¹H NMR spectrum of 2-isopropyl-4-nitrophenol **2l′′**

**¹³C NMR (151 MHz, CDCl₃)**

**Figure S37**. ¹³C NMR spectrum of 2-isopropyl-4-nitrophenol **2l′′**

**¹H NMR (600 MHz, CDCl₃)**

**Figure S38**. ¹H NMR spectrum of 2-ethyl-6-nitrophenol **2m′**

**¹³C NMR (151 MHz, CDCl₃)**

**Figure S39**. ¹³C NMR spectrum of 2-ethyl-6-nitrophenol **2m′**

**¹H NMR (600 MHz, CDCl₃)**

**Figure S40**. ¹H NMR spectrum of 2-ethyl-4-nitrophenol **2m′′**

**¹³C NMR (151 MHz, CDCl₃)**

**Figure S41**. ¹³C NMR spectrum of 2-ethyl-4-nitrophenol **2m′′**

**¹H NMR (600 MHz, CDCl₃)**

**Figure S42**. ¹H NMR spectrum of 2-iodo-6-nitrophenol **2n′**

**¹³C NMR (151 MHz, CDCl₃)**

**Figure S43**. ¹³C NMR spectrum of 2-iodo-6-nitrophenol **2n′**

**¹H NMR (600 MHz, CDCl₃)**

**Figure S44**. ¹H NMR spectrum of 2-iodo-4-nitrophenol **2n′′**

**¹³C NMR (151 MHz, CDCl₃)**

**Figure S45**. ¹³C NMR spectrum of 2-iodo-4-nitrophenol **2n′′**

**¹H NMR (600 MHz, CDCl₃)**

**Figure S46**. ¹H NMR spectrum of 4-fluoro-2-nitrophenol **2o**

**¹³C NMR (151 MHz, CDCl₃)**

**Figure S47**. ¹³C NMR spectrum of 4-fluoro-2-nitrophenol **2o**

**¹⁹F NMR (565 MHz, CDCl₃)**

**Figure S48**. ¹⁹F NMR spectrum of 4-fluoro-2-nitrophenol **2o**

**¹H NMR (600 MHz, CDCl₃)**

**Figure S49**. ¹H NMR spectrum of 4-chloro-2-nitrophenol **2p**

**¹³C NMR (151 MHz, CDCl₃)**

**Figure S50**. ¹³C NMR spectrum of 4-chloro-2-nitrophenol **2p**

**¹H NMR (600 MHz, CDCl₃)**

**Figure S51**. ¹H NMR spectrum of 5-fluoro-2-nitrophenol **2q′**

**¹³C NMR (151 MHz, CDCl₃)**

**Figure S52**. ¹³C NMR spectrum of 5-fluoro-2-nitrophenol **2q′**

**¹⁹F NMR (565 MHz, CDCl₃)**

**Figure S53**. ¹⁹F NMR spectrum of 5-fluoro-2-nitrophenol **2q′**

**¹H NMR (600 MHz, CDCl₃)**

**Figure S54**. ¹H NMR spectrum of 3-fluoro-4-nitrophenol **2q′′**

**¹³C NMR (151 MHz, CDCl₃)**

**Figure S55**. ¹³C NMR spectrum of 3-fluoro-4-nitrophenol **2q′′**

**¹⁹F NMR (565 MHz, CDCl₃)**

**Figure S56**. ¹⁹F NMR spectrum of 3-fluoro-4-nitrophenol **2q′′**

**¹H NMR (600 MHz, CDCl₃)**

**Figure S57**. ¹H NMR spectrum of 3-fluoro-2-nitrophenol **2q′′′**

**¹³C NMR (151 MHz, CDCl₃)**

**Figure S58**. ¹³C NMR spectrum of 3-fluoro-2-nitrophenol **2q′′′**

**¹⁹F NMR (565 MHz, CDCl₃)**

**Figure S59**. ¹⁹F NMR spectrum of 3-fluoro-2-nitrophenol **2q′′′**

**¹H NMR (600 MHz, CDCl₃)**

**Figure S60**. ¹H NMR spectrum of 4-methoxy-2-nitrophenol **2r**

**¹³C NMR (151 MHz, CDCl₃)**

**Figure S61**. ¹³C NMR spectrum of 4-methoxy-2-nitrophenol **2r**

**¹H NMR (600 MHz, CDCl₃)**

**Figure S62**. ¹H NMR spectrum of 2-methoxy-6-nitrophenol **2s′**

**¹³C NMR (151 MHz, CDCl₃)**

**Figure S63**. ¹³C NMR spectrum of 2-methoxy-6-nitrophenol **2s′**

**¹H NMR (600 MHz, CDCl₃)**

**Figure S64**. ¹H NMR spectrum of 2-methoxy-4-nitrophenol **2s′′**

**¹³C NMR (151 MHz, CDCl₃)**

**Figure S65**. ¹³C NMR spectrum of 2-methoxy-4-nitrophenol **2s′′**

**¹H NMR (600 MHz, CDCl₃)**

**Figure S66**. ¹H NMR spectrum of 4-(methylthio)-2-nitrophenol **2t**

**¹³C NMR (151 MHz, CDCl₃)**

**Figure S67**. ¹³C NMR spectrum of 4-(methylthio)-2-nitrophenol **2t**

**¹H NMR (600 MHz, CDCl₃)**

**Figure S68**. ¹H NMR spectrum of 4-chloro-2-methoxy-6-nitrophenol **2u**

**¹³C NMR (151 MHz, CDCl₃)**

**Figure S69**. ¹³C NMR spectrum of 4-chloro-2-methoxy-6-nitrophenol **2u**

**¹H NMR (600 MHz, CDCl₃)**

**Figure S70**. ¹H NMR spectrum of 4-(benzyloxy)-2-nitrophenol **2v**

**¹³C NMR (151 MHz, CDCl₃)**

**Figure S71**. ¹³C NMR spectrum of 4-(benzyloxy)-2-nitrophenol **2v**

**¹H NMR (600 MHz, CDCl₃)**

**Figure S72**. ¹H NMR spectrum of 2-methoxy-4-methyl-6-nitrophenol **2w**

**¹³C NMR (151 MHz, CDCl₃)**

**Figure S73**. ¹³C NMR spectrum of 2-methoxy-4-methyl-6-nitrophenol **2w**

**¹H NMR (600 MHz, CDCl₃)**

**Figure S74**. ¹H NMR spectrum of 1-nitronaphthalen-2-ol **2x**

**¹³C NMR (151 MHz, CDCl₃)**

**Figure S75**. ¹³C NMR spectrum of 1-nitronaphthalen-2-ol **2x**

**¹H NMR (600 MHz, CDCl₃)**

**Figure S76**. ¹H NMR spectrum of 2-nitronaphthalen-1-ol **2y**

**¹³C NMR (151 MHz, CDCl₃)**

**Figure S77**. ¹³C NMR spectrum of 2-nitronaphthalen-1-ol **2y**

**¹H NMR (600 MHz, CDCl₃)**

**Figure S78**. ¹H NMR spectrum of 7-bromo-1-nitronaphthalen-2-ol **2z**

**¹³C NMR (151 MHz, CDCl₃)**

**Figure S79**. ¹³C NMR spectrum of 7-bromo-1-nitronaphthalen-2-ol **2z**

**^1^H NMR (600 MHz, DMSO-*d₆*)**

**Figure S80**. ¹H NMR spectrum of ((9H-fluoren-9-yl)methoxy)carbonyl)-3-nitro-tyrosine **2aa**

**^13^C NMR (151 MHz, DMSO-*d₆*)**

**Figure S81**. ¹³C NMR spectrum of ((9H-fluoren-9-yl)methoxy)carbonyl)-3-nitro-tyrosine **2aa**

**¹H NMR (600 MHz, CDCl₃)**

**Figure S82**. ¹H NMR spectrum of 2-nitro-estradiol **2ab**

**¹³C NMR (151 MHz, CDCl₃)**

**Figure S83**. ¹³C NMR spectrum of 2-nitro-estradiol **2ab**

**¹H NMR (600 MHz, CDCl₃)**

**Figure S84**. ¹H NMR spectrum of 2-nitro-estrone **2ac**

**¹³C NMR (151 MHz, CDCl₃)**

**Figure S85**. ¹³C NMR spectrum of 2-nitro-estrone **2ac**

**¹H NMR (600 MHz, CDCl₃)**

**Figure S86**. ¹H NMR spectrum of 2,4-dibromo-6-nitrophenol **4a′**

**¹³C NMR (151 MHz, CDCl₃)**

**Figure S87**. ¹³C NMR spectrum of 2,4-dibromo-6-nitrophenol **4a′**

**¹H NMR (600 MHz, CDCl₃)**

**Figure S88**. ¹H NMR spectrum of 2,6-dibromo-4-nitrophenol **4a′′**

**¹³C NMR (151 MHz, CDCl₃)**

**Figure S89**. ¹³C NMR spectrum of 2,6-dibromo-4-nitrophenol **4a′′**

**¹H NMR (600 MHz, CDCl₃)**

**Figure S90**. ¹H NMR spectrum of 4-bromo-2,3-dimethyl-6-nitrophenol **4e′**

**¹³C NMR (151 MHz, CDCl₃)**

**Figure S91**. ¹³C NMR spectrum of 4-bromo-2,3-dimethyl-6-nitrophenol **4e′**

**¹H NMR (600 MHz, CDCl₃)**

**Figure S92**. ¹H NMR spectrum of 6-bromo-2,3-dimethyl-4-nitrophenol **4e′′**

**¹³C NMR (151 MHz, CDCl₃)**

**Figure S93**. ¹³C NMR spectrum of 6-bromo-2,3-dimethyl-4-nitrophenol **4e′′**

**¹H NMR (600 MHz, CDCl₃)**

**Figure S94**. ¹H NMR spectrum of 2-bromo-4-methyl-6-nitrophenol **4f**

**¹³C NMR (151 MHz, CDCl₃)**

**Figure S95**. ¹³C NMR spectrum of 2-bromo-4-methyl-6-nitrophenol **4f**

**¹H NMR (600 MHz, CDCl₃)**

**Figure S96**. ¹H NMR spectrum of 4-methyl-2,6-dinitrophenol **4g**

**¹³C NMR (151 MHz, CDCl₃)**

**Figure S97**. ¹³C NMR spectrum of 4-methyl-2,6-dinitrophenol **4g**

**¹H NMR (600 MHz, CDCl₃)**

**Figure S98**. ¹H NMR spectrum of 4,6-dibromo-3-methyl-2-nitrophenol **4h′**

**¹³C NMR (151 MHz, CDCl₃)**

**Figure S99**. ¹³C NMR spectrum of 4,6-dibromo-3-methyl-2-nitrophenol **4h′**

**¹H NMR (600 MHz, CDCl₃)**

**Figure S100**. ¹H NMR spectrum of 2,6-dibromo-3-methyl-4-nitrophenol **4h′′**

**¹³C NMR (151 MHz, CDCl₃)**

**Figure S101**. ¹³C NMR spectrum of 2,6-dibromo-3-methyl-4-nitrophenol **4h′′**

**¹H NMR (600 MHz, CDCl₃)**

**Figure S102**. ¹H NMR spectrum of 4-bromo-2-methyl-6-nitrophenol **4i′**

**¹³C NMR (151 MHz, CDCl₃)**

**Figure S103**. ¹³C NMR spectrum of 4-bromo-2-methyl-6-nitrophenol **4i′**

**¹H NMR (600 MHz, CDCl₃)**

**Figure S104**. ¹H NMR spectrum of 2-bromo-6-methyl-4-nitrophenol **4i′′**

**¹³C NMR (151 MHz, CDCl₃)**

**Figure S105**. ¹³C NMR spectrum of 2-bromo-6-methyl-4-nitrophenol **4i′′**

**¹H NMR (600 MHz, CDCl₃)**

**Figure S106**. ¹H NMR spectrum of 2-bromo-4-fluoro-6-nitrophenol **4j**

**¹³C NMR (151 MHz, CDCl₃)**

**Figure S107**. ¹³C NMR spectrum of 2-bromo-4-fluoro-6-nitrophenol **4j**

**¹⁹F NMR (565 MHz, CDCl₃)**

**Figure S108**. ¹⁹F NMR spectrum of 2-bromo-4-fluoro-6-nitrophenol **4j**

**¹H NMR (600 MHz, CDCl₃)**

**Figure S109**. ¹H NMR spectrum of 4-bromo-2-fluoro-6-nitrophenol **4k′**

**¹³C NMR (151 MHz, CDCl₃)**

**Figure S110**. ¹³C NMR spectrum of 4-bromo-2-fluoro-6-nitrophenol **4k′**

**¹⁹F NMR (565 MHz, CDCl₃)**

**Figure S111**. ¹⁹F NMR spectrum of 4-bromo-2-fluoro-6-nitrophenol **4k′**

**¹H NMR (600 MHz, CDCl₃)**

**Figure S112**. ¹H NMR spectrum of 2-bromo-6-fluoro-4-nitrophenol **4k′′**

**¹³C NMR (151 MHz, CDCl₃)**

**Figure S113**. ¹³C NMR spectrum of 2-bromo-6-fluoro-4-nitrophenol **4k′′**

**¹⁹F NMR (565 MHz, CDCl₃)**

**Figure S114.** ¹⁹F NMR spectrum of 2-bromo-6-fluoro-4-nitrophenol **4k′′**

**¹H NMR (600 MHz, CDCl₃)**

**Figure S115**. ¹H NMR spectrum of 4-bromo-2-chloro-6-nitrophenol **4l′**

**¹³C NMR (151 MHz, CDCl₃)**

**Figure S116**. ¹³C NMR spectrum of 4-bromo-2-chloro-6-nitrophenol **4l′**

**¹H NMR (600 MHz, CDCl₃)**

**Figure S117**. ¹H NMR spectrum of 2-bromo-6-chloro-4-nitrophenol **4l′′**

**¹³C NMR (151 MHz, CDCl₃)**

**Figure S118**. ¹³C NMR spectrum of 2-bromo-6-chloro-4-nitrophenol **4l′′**

**¹H NMR (600 MHz, CDCl₃)**

**Figure S119**. ¹H NMR spectrum of 2,4-dichloro-6-nitrophenol **4m**

**¹³C NMR (151 MHz, CDCl₃)**

**Figure S120**. ¹³C NMR spectrum of 2,4-dichloro-6-nitrophenol **4m**

**¹H NMR (600 MHz, CDCl₃)**

**Figure S121**. ¹H NMR spectrum of 2,4-diiodo-6-nitrophenol **4n′**

**¹³C NMR (151 MHz, CDCl₃)**

**Figure S122**. ¹³C NMR spectrum of 2,4-diiodo-6-nitrophenol **4n′**

**¹H NMR (600 MHz, CDCl₃)**

**Figure S123**. ¹H NMR spectrum of 2,6-diiodo-4-nitrophenol **4n′′**

**¹³C NMR (151 MHz, CDCl₃)**

**Figure S124**. ¹³C NMR spectrum of 2,6-diiodo-4-nitrophenol **4n′′**

**¹H NMR (600 MHz, CDCl₃)**

**Figure S125**. ¹H NMR spectrum of 2,6-di-tert-butyl-4-methyl-4-nitrocyclohexa-2,5-dien-1-one **4o**

**¹³C NMR (151 MHz, CDCl₃)**

**Figure S126**. ¹³C NMR spectrum of 2,6-di-tert-butyl-4-methyl-4-nitrocyclohexa-2,5-dien-1-one **4o**

**¹H NMR (600 MHz, CDCl₃)**

**Figure S127**. ¹H NMR spectrum of 2,6-di-tert-butyl-4-ethyl-4-nitrocyclohexa-2,5-dien-1-one **4p**

**¹³C NMR (151 MHz, CDCl₃)**

**Figure S128**. ¹³C NMR spectrum of 2,6-di-tert-butyl-4-ethyl-4-nitrocyclohexa-2,5-dien-1-one **4p**

**¹H NMR (600 MHz, CDCl₃)**

**Figure S129**. ¹H NMR spectrum of methyl 3-(3,5-di-tert-butyl-1-nitro-4-oxocyclohexa-2,5-dien-1-yl)propanoate **4q**

**¹³C NMR (151 MHz, CDCl₃)**

**Figure S130**. ¹³C NMR spectrum of methyl 3-(3,5-di-tert-butyl-1-nitro-4-oxocyclohexa-2,5-dien-1-yl) propanoate **4q**

**¹H NMR (600 MHz, CDCl₃)**

**Figure S131**. ¹H NMR spectrum of 2,6-dichlorocyclohexa-2,5-diene-1,4-dione **4r**

**¹³C NMR (151 MHz, CDCl₃)**

**Figure S132**. ¹³C NMR spectrum of 2,6-dichlorocyclohexa-2,5-diene-1,4-dione **4r**

**¹H NMR (600 MHz, CDCl₃)**

**Figure S133**. ¹H NMR spectrum of 3,3′,5,5′-tetramethyl-[1,1′-bi(cyclohexylidene)]-2,2′,5,5′-tetraene-4,4′-dione **4s**

**¹³C NMR (151 MHz, CDCl₃)**

**Figure S134**. ¹³C NMR spectrum of 3,3′,5,5′-tetramethyl-[1,1′-bi(cyclohexylidene)]-2,2′,5,5′-tetraene-4,4′-dione **4s**

**¹H NMR (600 MHz, CDCl₃)**

**Figure S135**. ^1^H NMR spectrum of 3,3′,5,5′-tetra-tert-butyl-[1,1′-bi(cyclohexylidene)]-2,2′,5,5′-tetraene-4,4′-dione **4t**

**¹³C NMR (151 MHz, CDCl₃)**

**Figure S136**. ¹³C NMR spectrum of 3,3′,5,5′-tetra-tert-butyl-[1,1′-bi(cyclohexylidene)]-2,2′,5,5′-tetraene-4,4′-dione **4t**

**¹H NMR (600 MHz, CDCl₃)**

**Figure S137**. ¹H NMR spectrum of 2,6-di-tert-butylcyclohexa-2,5-diene-1,4-dione **4u**

**¹³C NMR (151 MHz, CDCl₃)**

**Figure S138**. ¹³C NMR spectrum of 2,6-di-tert-butylcyclohexa-2,5-diene-1,4-dione **4u**

# References

[1]	Bruker AXS Inc., *SAINT, Version 8.40B*, Bruker AXS Inc., Madison, WI, USA, **2016**.

[2]	G. Sheldrick, *SADABS, Version 2016/2*, Bruker AXS Inc., Madison, WI, USA, **2016**.

[3]	G. M. Sheldrick, *Acta Crystallogr.* **2015***, A71*, 3–8*.*

[4]	G. Sheldrick, *Acta Crystallogr.* **2015***, C71*, 3–8.

[5]	O. V. Dolomanov, L. J. Bourhis, R. J. Gildea, J. A. Howard, H. Puschmann, *J. Appl. Crystallogr.* **2009**, *42*, 339-341.

[6]	W. Koch, M. C. Holthausen, *A Chemist's Guide to Density Functional Theory*, Wiley-VCH, Weinheim, **2002**.

[7]	Gaussian 16, rev. C.01, M. J. Frisch, G. W. Trucks, H. B. Schlegel, G. E. Scuseria, M. A. Robb, J. R. Cheeseman, G. Scalmani, V. Barone, G. A. Petersson, H. Nakatsuji, X. Li, M. Caricato, A. V. Marenich, J. Bloino, B. G. Janesko, R. Gomperts, B. Mennucci, H. P. Hratchian, J. V. Ortiz, A. F. Izmaylov, J. L. Sonnenberg, D. Williams-Young, F. Ding, F. Lipparini, F. Egidi, J. Goings, B. Peng, A. Petrone, T. Henderson, D. Ranasinghe, V. G. Zakrzewski, J. Gao, N. Rega, G. Zheng, W. Liang, M. Hada, M. Ehara, K. Toyota, R. Fukuda, J. Hasegawa, M. Ishida, T. Nakajima, Y. Honda, O. Kitao, H. Nakai, T. Vreven, K. Throssell, J. A. Montgomery, Jr., J. E. Peralta, F. Ogliaro, M. J. Bearpark, J. J. Heyd, E. N. Brothers, K. N. Kudin, V. N. Staroverov, T. A. Keith, R. Kobayashi, J. Normand, K. Raghavachari, A. P. Rendell, J. C. Burant, S. S. Iyengar, J. Tomasi, M. Cossi, J. M. Millam, M. Klene, C. Adamo, R. Cammi, J. W. Ochterski, R. L. Martin, K. Morokuma, O. Farkas, J. B. Foresman, and D. J. Fox, Gaussian, Inc., Wallingford CT, **2016.**

[8]	Y. Zhao, D. G. Truhlar, *Theor. Chem. Acc.* **2008**, *120*, 215-241.

[9]	S. Grimme, J. Antony, S. Ehrlich, H. Krieg, *J. Chem. Phys.* **2010**, *132*, 154104.

[10]	F. Weigend, R. Ahlrichs, *Phys. Chem. Chem. Phys.* **2005**, *7*, 3297-3305.

[11]	K. B. Wiberg, *Tetrahedron* **1968**, *24*, 1083-1096.

[12]	A. E. Reed, R. B. Weinstock, F. Weinhold, *J. Chem. Phys.* **1985**, *83*, 735-746.

[13]	R. Dennington, T. A. Keith, J. M. Millam, GaussView 6.0.16 Semichem Inc., Shawnee Mission, KS, USA, **2016**.

[14]	T. Renningholtz, E. R. X. Lim, M. J. James, C. Trujillo, *Org. Biomol. Chem.* **2024**, *22*, 6166-6173.

[15]	M. C. Aragoni, C. Caltagirone, V. Lippolis, E. Podda, A. M. Z. Slawin, J. D. Woollins, A. Pintus, M. Arca, *Inorg. Chem.* **2020**, *59*, 17385-17401.

[16]	Y. Mao, Y. Liu, Y. Hu, L. Wang, S. Zhang, W. Wang, *ACS Catal.* **2018**, *8*, 3016-3020.

[17]	A. Ding, Y. Zhang, Y. Chen, R. Rios, J. Hu, H. Guo, *Tetrahedron Lett.* **2019**, *60*, 660-663.

[18]	M. S. Kumar, K. Rajanna, K. R. Reddy, M. Venkateswarlu, P. Venkanna, *Synth. Commun.* **2013**, *43*, 2672-2677.

[19]	Y. Sun, H. Shi, J. He, K. Yang, D. Yin, D. Xiao, Y. Du, *J. Org. Chem.* **2025**, *90*, 2328-2340.

[20]	H. Brockmann, F. Seela, *Chem. Ber.* **1971**, *104*, 2751-2771.

[21]	J. I. Murray, N. J. Flodén, A. Bauer, N. D. Fessner, D. L. Dunklemann, O. Bob‐Egbe, H. S. Rzepa, T. Bürgi, J. Richardson, A. C. Spivey, *Angew. Chem. Int. Ed.* **2017**, *129*, 5854-5858.

[22]	T. Long, L. Liu, Y. Tao, W. Zhang, J. Quan, J. Zheng, J. D. Hegemann, M. Uesugi, W. Yao, H. Tian, *Angew. Chem. Int. Ed.* **2021**, *60*, 13414-13422.

[23]	W.-P. Li, G. Cheng, S.-Y. Li, C.-Z. Lin, X.-Y. Guan, D.-X. Bing, J. Cao, D. Zhu, Q.-H. Deng, *J. Org. Chem.* **2022**, *87*, 3834-3840.

[24]	M. Wąsińska, A. Korczewska, M. Giurg, J. Skarżewski, *Synth. Commun.* **2015**, *45*, 143-150.

[25]	T. Matsumoto, A. Yamauchi, J. Ishikawa, G.-H. Jin, J. Matsumoto, Y. Fueda, M. Yasuda, *Int. J. Org. Chem.* **2016**, *7*, 1-11.

[26]	J. A. Rivera‐Nevares, J. F. Wyman, D. L. Von Minden, N. Lacy, M. L. Chabinyc, A. V. Fratini, D. A. Macys, *Environ. Toxicol. Chem.* **1995**, *14*, 251-256.

[27]	M. Vojtičková, J. Dobiaš, G. Hanquet, G. Addová, R. Cetin-Atalay, D. C. Yildirim, A. Boháč, *Eur. J. Med. Chem.* **2015**, *103*, 105-122.

[28]	T. Yang, X. Li, S. Deng, X. Qi, H. Cong, H.-G. Cheng, L. Shi, Q. Zhou, L. Zhuang, *JACS Au* **2022**, *2*, 2152-2161.

[29]	C. V. Ramana, R. S. Phatake, Council of Scientific and Industrial Research (CSIR),  Council of Scientific and Industrial Research (CSIR), United States, US 2016/0168114 A1, **2016**.

[30]	R. Azadi, Z. Nazari-Far, *Lett. Org. Chem.* **2015**, *12*, 136-140.

[31]	D. Habibi, M. A. Zolfigol, M. Shiri, A. Sedaghat, *S. Afr. J. Chem.* **2006**, *59*, 93–96.

[32]	D. Thongkum, T. Tuntulani, *Tetrahedron* **2011**, *67*, 8102-8109.

[33]	K. A. Juárez-Ornelas, J. O. C. Jiménez-Halla, T. Kato, C. R. Solorio-Alvarado, K. Maruoka, *Org. Lett.* **2019**, *21*, 1315-1319.

[34]	P. Martin, *Helv. Chim. Acta* **1989**, *72*, 1554-1582.

[35]	S. T. Caldwell, A. G. Cairns, M. Olson, S. Chalmers, M. Sandison, W. Mullen, J. G. McCarron, R. C. Hartley, *Tetrahedron* **2015**, *71*, 9571-9578.

[36]	A. Ghorbani-Choghamarani, H. Goudarziafshar, M. Nikoorazm, S. Yousefi, *Can. J. Chem.* **2009**, *87*, 1144-1147.

[37]	F. Nemati, H. Kiani, Y. S. Hayeniaz, *Synth. Commun.* **2011**, *41*, 2985-2992.

[38]	N. Venkatesham, K. R. Reddy, K. Rajanna, P. Veerasomaiah, *Synth. React. Inorg. Met.-Org. Nano-Met. Chem.* **2014**, *44*, 921-926.

[39]	P. Niederhafner, M. Šafařík, E. Brichtová, J. Šebestík, *Amino Acids* **2016**, *48*, 1087-1098.

[40]	D. Bandyopadhyay, G. Rivera, J. L. Sanchez, J. Rivera, J. C. Granados, A. M. Guerrero, F.-M. Chang, R. K. Dearth, J. D. Short, B. K. Banik, *Eur. J. Med. Chem.* **2014**, *82*, 574-583.

[41]	Y. Zheng, Q.-Q. Hu, Q. Huang, Y. Xie, *Org. Lett.* **2024**, *26*, 3316-3320.

[42]	P. V. Ghorpade, D. A. Pethsangave, S. Some, G. S. Shankarling, *J. Org. Chem.* **2018**, *83*, 7388-7397.

[43]	G.-W. Wang, J. Gao, *Green Chem.* **2012**, *14*, 1125-1131.

[44]	S. P. Blum, C. Nickel, L. Schäffer, T. Karakaya, S. R. Waldvogel, *ChemSusChem* **2021**, *14*, 4936-4940.

[45]	K. Babaoglu, G. Brizgys, J. Cha, X. Chen, H. Guo, R. L. Halcomb, X. Han, R. Huang, H. Liu, R. McFadden, M. L. Mitchell, Y. Qi, P. A. Roethle, L. Xu, H. Yang*, Vol. US20130281433A1* (Ed.: U.S.P.a.T.Office), Gilead Sciences Inc., United States, **2013**.

[46]	Y. Zeng, J. Xiao, L. Shi, Y. Li, Y. Xu, J. Zhou, X. Dong, H. Hou, C. Zhong, G. Cheng, *Eur. J. Med. Chem.* **2025**, *287*, 117293.

[47]	E. Nagy, C. Vincent, T. N. P. Tran, V. Nair, P. Jones, L. M. Mustachio*, Vol. WO2026039659A1*, University of Texas at Austin, WIPO (PCT), **2026**.

[48]	H. Y. Choi, D. Y. Chi, *J. Am. Chem. Soc.* **2001**, *123*, 9202-9203.

[49]	A. Golounin, V. Sokolenko, O. Zakharova, E. Shor, M. Tovbis, *Russ. J. Appl. Chem.* **2007**, *80*, 887-890.

[50]	Y. Kiran, T. Konakahara, N. Sakai, *Synthesis* **2008**, *2008*, 2327-2332.

[51]	J. Chýlková, O. Machalický, M. Tomášková, R. Šelešovská, T. Navrátil, *Anal. Lett.* **2016**, *49*, 92-106.

[52]	F. Sprang, J. D. Herszman, S. R. Waldvogel, *Green Chem.* **2022**, *24*, 5116-5124.

[53]	S. Néron, M. Morency, L. Chen, T. Maris, D. Rochefort, R. Iftimie, J. D. Wuest, *J. Org. Chem.* **2022**, *87*, 7673-7695.

[54]	S. Gupta, D. C. Kakkarakkal, S. C. E. Stieber, S. Kundu, *Eur. J. Inorg. Chem.* **2025**, *28*, e202500113.

[55]	M. Konwar, T. Das, A. Das, *Chem. Eur. J.* **2025**, *31*, e202403135.
